# Systematic Studies on the Protocol and Criteria for Selecting a Covalent Docking Tool

**DOI:** 10.3390/molecules24112183

**Published:** 2019-06-10

**Authors:** Chang Wen, Xin Yan, Qiong Gu, Jiewen Du, Di Wu, Yutong Lu, Huihao Zhou, Jun Xu

**Affiliations:** 1 Research Center for Drug Discovery, School of Pharmaceutical Sciences, Sun Yat-Sen University, 132 East Circle at University City, Guangzhou 510006, China; wench7@mail2.sysu.edu.cn (C.W.); yanx28@mail.sysu.edu.cn (X.Y.); guqiong@mail.sysu.edu.cn (Q.G.); jiewen.du@xtalpi.com (J.D.); 2 National Supercomputer Center in Guangzhou & School of Data and Computer Science, Sun Yat-Sen University, 132 East Circle at University City, Guangzhou 510006, China; wudi27@mail.sysu.edu.cn (D.W.); yutong.lu@nscc-gz.cn (Y.L.)

**Keywords:** drug design, chemoinformatics, molecular modeling, covalent docking, protein binding

## Abstract

With the resurgence of drugs with covalent binding mechanisms, much attention has been paid to docking methods for the discovery of targeted covalent inhibitors. The existence of many available covalent docking tools has inspired development of a systematic and objective procedure and criteria with which to evaluate these programs. In order to find a tool appropriate to studies of a covalently binding system, protocols and criteria are proposed for protein–ligand covalent docking studies. This paper consists of three sections: (1) curating a standard data set to evaluate covalent docking tools objectively; (2) establishing criteria to measure the performance of a tool applied for docking ligands into a complex system; and (3) creating a protocol to evaluate and select covalent binding tools. The protocols were applied to evaluate four covalent docking tools (MOE, GOLD, CovDock, and ICM-Pro) and parameters affecting covalent docking performance were investigated.

## 1. Introduction

Covalent inhibitors have a reactive functional group, sometimes known as a warhead, which can form a chemical bond with a target. In the past decade, there has been a growing interest in the design of drugs that can form a covalent bond with drug targets [1]. Nearly 30% of the marketed drugs targeting enzymes are known to act by covalent addition, and targeted covalent inhibitors (TCIs) are becoming important [2]. Drugs such as Boceprevir [3], which operates by nucleophilic addition, Osimertinib [4] (Michael addition), and Clopidogrel [5] (disulfide formation) have been approved by the Food and Drug Administration (FDA). The pharmacological advantages of covalent inhibitors are being extensively studied, and it has been shown that covalent inhibitors can achieve longer drug residence times than non-covalent inhibitors [6] and improve target selectivity [7,8]. New methods for TCI design have been reported [9,10,11,12,13,14] and there is an increasing demand for virtual screening of TCIs [15,16,17,18]. Currently, many covalent docking tools are available, and an online comparative evaluation of covalent docking tools is available. Keserű and colleagues have highlighted the key factors influencing the docking performance of the tools that have been investigated and they give guidelines for selecting the optimal combination of warheads, ligands, and tools for different systems [19].

It is worth mentioning a special form of covalent bonds: metal coordination bonds. Most metalloligands not only interact with biomolecules by binding to the therapeutic targets but also influence transportation in the blood or cell in which they are engaged. Medicinal inorganic chemistry has been studied for years [20] and the computational challenge for predicting possible metal-mediated binding with protein-ligand docking methods is how to cope with the formation of one or more coordination bonds [21]. There are some explorative works to tackle the limitations of the protein-ligand docking methods [22,23], and they did well in simulating the covalent bonds between a protein and a metal species; our work focuses on common covalent ligands.

In this paper, we examine studies of the protocols and criteria used to select a covalent docking tool. Compared with non-covalent docking, the covalent docking process is sophisticated, and to evaluate these docking tools, we focus on the following issues:

(1) classification of warhead chemotypes and the targets in receptors,
(2) provision of a standard unbiased benchmark data set to test covalent docking tools objectively, and
(3) elaboration of criteria used to measure the performance of covalent docking tools used in a warhead-receptor complex.

Our goals are (a) to provide a general protocol to evaluate existing covalent docking tools and evaluate new tools in the future; (b) to provide benchmark data for covalent docking evaluation (BCDE); and (c) to elaborate criteria enabling measurement of the performance of covalent docking tools.

This research involved 330 covalent complexes, original covalent ligand files, and the reaction files, which show the formation of covalent bonds between warhead atoms and reactive residues. The protocols have been tested with commonly used software packages (MOE, GOLD, CovDock, and ICM-Pro). More and more covalent docking tools are becoming available, such as DOCKTITE [24], AutoDock [25], Dockovalent [26], and CovalentDock Cloud [27]. Instead of regularly evaluating the docking tools [28,29,30], we developed this protocol that may be useful for selecting a covalent docking tool for a specific target. The challenges for covalent docking tools have been previously summarized [31,32].

## 2. Results

### 2.1. Covalent Binding Reaction Classification

Based on the combinations of covalent binding reaction types (nucleophilic-addition/substitution/ring-opening/disulfide formation) and binding anchor types (cysteine/serine), covalent binding reactions can be divided into seven classes, as shown in Table 1. The warheads are divided into 15 classes belonging to corresponding reaction types. Each warhead has one or more cores, which are used to covalently connect ligands and receptors.

The full list of Protein Data Bank (PDB, http://www.rcsb.org/) accession codes of the BCDE data set and additional docking information are available in the Supplementary Information, Table S1.

### 2.2. Evaluation of Covalent Docking Tools

**Figure 1:**
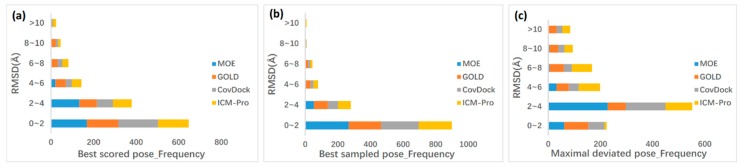
Stacked bar chart of docking performance over all 330 reference binding modes. The frequency of all RMSDs from the crystal ligand among best scored pose (**a**), best sampled pose (**b**), and maximal deviated pose (**c**).

The top-ranked binding mode (Best Scored Pose) and the conformation with minimum root-mean-square deviation (RMSD) values with crystal pose among all docked ligand poses (Best Sampled Pose) were used to evaluate the docking tools. The Best Sampled Pose might be a metric with little difference between tested tools because the sampling method is more developed than with the scoring function. This work was conducted based on the fact that covalent bonds are predefined and most scoring functions typically neglect the entire contribution from covalent bond formation. Specifically, we are interested in the maximal deviated pose for a comprehensive inspection of the evaluation. This measure has been verified to confirm the quality of the produced docking predictions [33,34]. For each of the 330 binding modes retained in the benchmark data set, the RMSD of three poses from the reference binding mode was determined (Figure 1), which reveals that all docking tools were able to find a near-native pose (RMSD at most 2.0 Å) for 144~187 of the 330 best scored poses and 203~267 of the 330 best sampled poses. In terms of the maximal deviated pose, ICM-Pro has more frequencies among the large region (RMSD > 2.0 Å) compared with other tools. The median and maximum RMSD distributions with RMSDs of 5.38 Å and 14.20 Å, respectively, show a distinct difference with the two poses. All simulations demonstrate that the employed docking tools generated qualified data to provide a reasonable test scenario for our work.

All docking RMSD values are listed in Table S1 in the Supplementary Information, and computational costs in terms of docking time as well as disk space are listed in Figure S1. The median RMSD calculations for every protein are listed in Figures S2 and S3. Three criteria (precision, generality, and robustness) were introduced to evaluate a covalent docking tool for a given target. More discussions on “precision, generality, and robustness” can also be found in Section 4.2.

- Precision. *P*(*i*,*x*), the precision for the *i*th docking tool (*i* ∈ MOE, GOLD, CovDock, ICM-Pro) using the *x*th RMSD measurement *x* ∈ (Best Scored Pose, Best Sampled Pose), measured for each covalent docking tool demonstrates different docking performances for different reaction classes and warheads. *P*(*i*,*x*) can be calculated with formula (1) as follows:
  (1) $$P\left(i,x\right)=Median_{j=1}^{m}\left(Median_{k}^{n}\left(RMSD_{i,x,j,k}\right)\right)$$
  where m is the total number of warhead types or the total number of receptor types, and n is the total number of co-crystal structures for a given warhead type, or a given receptor type. RMSD *i*,*x*,*j*,*k* is the RMSD value between the crystallographic ligand and the docked ligand pose produced by the *i*th docking tool with *x*th measurement for the *k*th co-crystal structure of the *j*th warhead type (the *j*th receptor type). Thus, a lower *P*(*i*,*x*) is better.

As shown in Table 2, the heat maps (red = worst and green = best) show the RMSD medians of the top-ranked poses. With nitrile (Cys), for example, GOLD seems better than other tools with both precision criteria. Using the medians as overall docking precision measures that cross the reaction classes (the last line in Table 2), MOE is better than other tools in terms of the Best Scored Pose (Median RMSD = 1.94 Å), and CovDock seems better than other tools in terms of the Best Sampled Pose (Median RMSD = 1.33 Å). We recognize that some warheads, such as alkyne, guanyl, nitrile (Ser), phosphonyl, and lactone appear no more than three times in the current RCSB PDB database. The evaluation protocol reported here can be repeated in the future when more instances are reported in the RCSB PDB database, or more covalent docking tools must be evaluated.

Table 3 demonstrates the relationships between the covalent tools and receptor types. With hydrolase, for example, CovDock seems better than other tools with both precision criteria. Using the medians as overall docking precision measures that cross the reaction classes (the last line at Table 3), CovDock is better than other tools in terms of the Best Scored Pose (Median RMSD = 1.71 Å); and GOLD seems better than other tools in terms of the Best Sampled Pose (Median RMSD = 1.17 Å).

**Figure 2:**
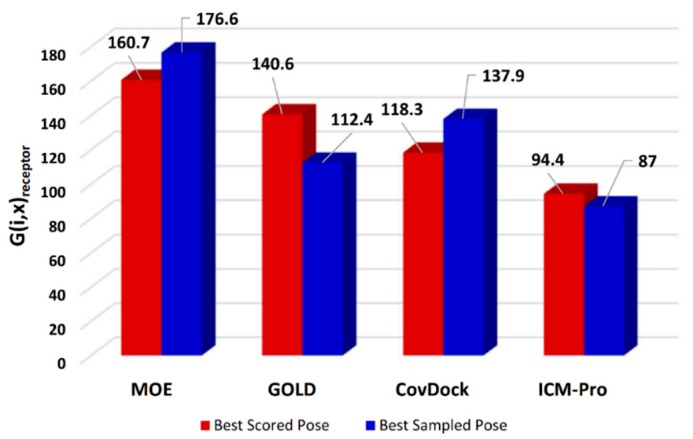
The generalities for the four docking tools based on receptor types.

Generality. Generality is measured for the overall precision across all types of receptors or warheads for a given tool. The G (*i*,*x*), generality of the *i*th docking tool with the *x*th RMSD measurement can be calculated using the following formulas:

(2) $$G{\left(i,x\right)}_{receptor\;type}=\overset{m_{1}}{\underset{j=1}{\sum }}{\left(\frac{\sum_{l=1}^{n}{\left(k_{i,j,l,x}\right)}_{\;}}{N_{i,j}}\right)}_{i}\times 100$$

(3) $$G{\left(i,x\right)}_{warhead\;type}=\overset{m_{2}}{\underset{j=1}{\sum }}{\left(\frac{\sum_{l=1}^{n}{\left(k_{i,j,l,x}\right)}_{\;}}{N_{i,j}}\right)}_{i}\times 100$$

where *G*(*i*,*x*) is the generality score for the *i*th docking tool using the *x*th RMSD measurement *x* ∈ (Best Scored Pose, Best Sampled Pose); *i* is for the *i*th docking tool (in this study, *i* ∈ MOE, GOLD, CovDock, ICM-Pro); *n* is the number of uniportIDs or warhead-cores; *m*_1_ is the number of receptor types tested; *m*_2_ is the number of the warhead types tested; *N_j_* is the number of total co-crystal structures for a given receptor type or a given warhead type used to evaluate the *i*th docking tool; and *k*_*i*,*j*,*l*,*x*_ is the number of the best median RMSD values among all the docking tools for the *i*th tool, the *j*th receptor type (or the *j*th warhead type), and the *l*th uniportID (or the *l*th warhead core-structure), measured with the *x*th method. The detailed calculations are listed in Figures S1 and S2 and Tables S2–S9. Thus, a higher G (*i*,*x*) is better. The G (*i*,*x*) for the four docking tools based on receptor types are shown in Figure 2.

**Figure 3:**
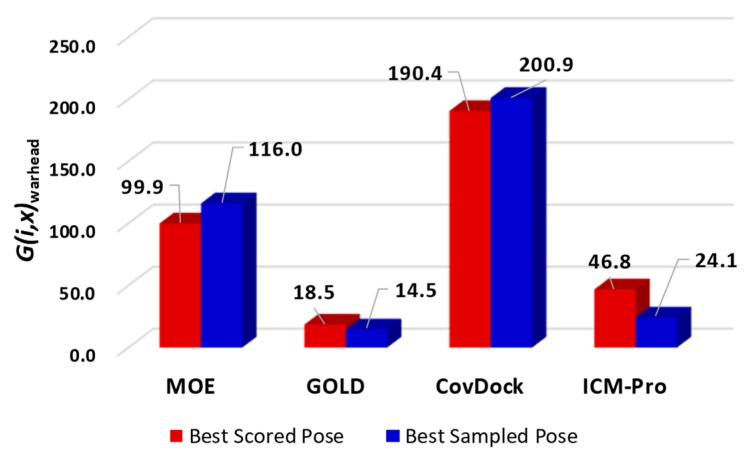
The generalities for the four docking tools based on warhead types.

In terms of the overall generalities across all types of receptors for covalent docking tools, MOE appears to deliver a superior performance. However, the generality analyses based on warhead types show that Schrödinger’s CovDock has a significantly better performance (Figure 3).

Mann-Whitney U-test results show that the above analyses are statistically significant (*p* < 0.05). The detailed U-test results are listed in SI Tables S10 and S11. As shown in Table S8, the results of the analyses using Best Sampled Pose are not statistically significant. Therefore, we suggest using the Best Scored Pose method to evaluate docking tools.

Combining the results in Figure 2 and Figure 3, we observed that MOE consistently produces superior docking results. However, if the generalities are based on warhead types, Schrödinger’s CovDock is superior to other docking tools.

**Figure 4:**
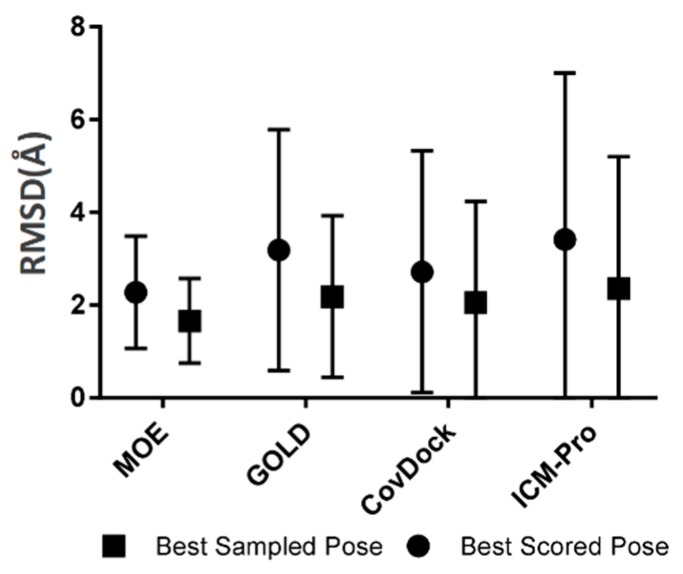
The robustness analysis results for the four docking tools. The details are listed in Table S12.

- **Robustness**. The robustness of a covalent docking tool is measured by the standard deviation of all RMSDs between native ligand poses and the docked poses covering all tested covalent binding complexes in the BCDE data set. The robustness analysis results for the four docking tools are depicted in Figure 4.

Based on Figure 4, we observed that ICM-Pro has the largest RMSD standard deviation, i.e., the lowest robustness, and MOE has the narrowest RMSD standard deviation, or the most robust character.

This study found that the overall performances of CovDock and MOE are similar, and the accuracy of docking is slightly better than obtained with GLOD and ICM-Pro, the other two tools with similar performances. The precise values vary depending on whether the subject is a receptor or a ligand. We found that different docking tools have different abilities when dealing with different targets and different warheads. In the best scored pose and the best sampled pose, the generality of CovDock is 13, that is, when processing 13 kinds of receptors, it is possible to obtain a lower RMSD than the other three tools, and these 13 kinds account for 41% of the entire effective target set. GOLD differs greatly for different targets, and gives better performance than other software on the three targets of Cathepsin S (CATS), Replicase polyprotein lab (REP), and Penicillin-binding protein A (PBPA) even though it performs slightly more poorly on the complete target set.

### 2.3. Profiling Covalent Docking Tools

**Figure 5:**
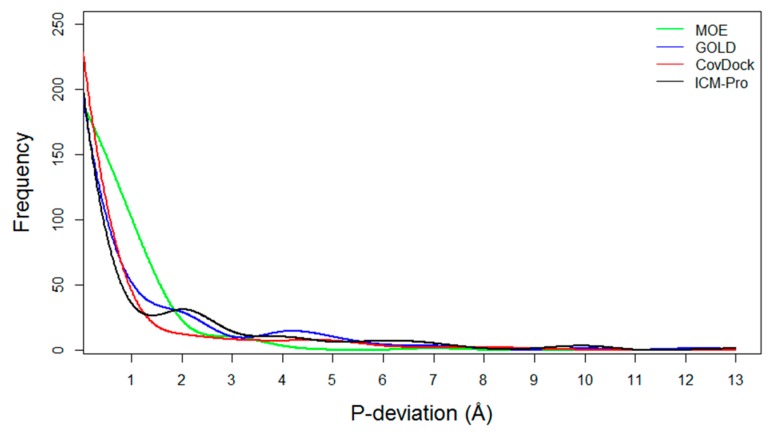
The P-deviation distributions for all docking tools.

**Figure 6:**
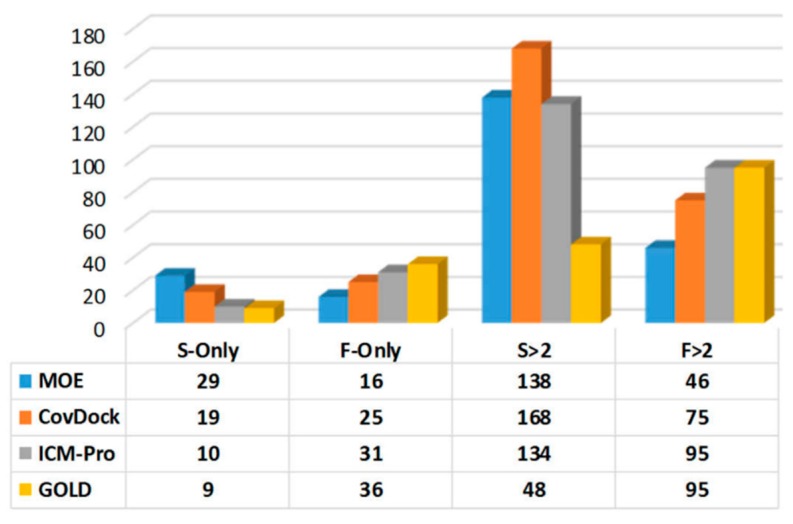
The profiles for the four docking tools.

In order to comprehensively estimate the capacity to produce a correct covalent binding pose for a docking tool, we investigated the following parameters:

- **Pose deviation and success and failure counts**: For a co-crystal structure, a docking tool can generate two RMSD values, Best Scored Pose, and Best Sampled Pose, denoted S_1_ and S_2_, respectively. Also, (S_1_–S_2_) is denoted as P-deviation, representing the difference between the two measurements of docking accuracies. For a given docking tool, if (S_1_ ≤ 2.0 Å) then it is considered as a success count (S-count); If (S_1_ > 2.0 Å) and (S_2_ > 2.0 Å) then it is considered as a failure count (F-count). The P-deviations, S-counts, and F-counts for the four evaluated docking tools have been computed and are listed in Table S13.
- **P-deviation distributions**. These distributions for all docking tools can be used to measure docking error ranges of the tools as shown in Figure 5 (the data are listed in Table S14). MOE (green) and CovDock (red) are closer to Gaussian distribution. GOLD (blue) and ICM-Pro (black) have two significant waves around 2Å and 4.5 Å suggesting that these tools have extraordinary errors around the 2Å and 4.5 Å ranges.
- **Parameters with which to profile a docking tool**. While analyzing the docking results, we revealed four parameters that can characterize a docking tool: (a) S-Only count—the number of cases in which only one docking tool predicted the successful poses, (b) F-Only count—the number of cases in which only one docking tool predicted the failing poses, (c) S > 2—the number of cases in which at least two docking tools predicted the successful poses, and (d) F > 2—the number of cases in which at least two docking tools predicted the failing poses. These parameters were derived from Table S13, and are listed in Table S15. The profiles for the four docking tools are depicted in Figure 6.

In summary, there is a greater possibility for MOE to consistently produce precise and robust covalent docking results for many targets. However, CovDock can be better than MOE if the generality is for various warhead types, although its robustness is not as good as that of MOE. Based on the profiling analytic results in Figure 6, it can be concluded that MOE can predict the most correct binding poses while other tools fail, and can predict the least number of failing poses while other tools can correctly predict more failing poses. Both CovDock and MOE can predict the most correct binding poses while at least one other tool can do just as well (the S > 2 column in Figure 6), although CovDock is significantly better than MOE. MOE, again, predicts the least failure poses while other tools perform equally as poorly (the F > 2 column in Figure 6).

These studies are based on a test set of existing data, assessed on 20 December 2017, and current versions of the docking tools. We believe the observations may change over time along with the technology improvements and the increasing size of the BCDE data set. We will update the BCDE database when possible.

## 3. Discussion

Keserű and colleagues’ paper is for comparatively evaluating covalent docking tools. Our work, however, focuses on proposing a protocol to select a suitable covalent docking tool for a specific target. Our intention is not to generally rank existing covalent docking tools, because each docking tool can be more favorable to a set of specific targets. It is commonly accepted that there is no docking tool that is superior to the other tools for all protein targets.

The parameters for performance measures in Table 2 and Table 3 and Figure 4, Figure 5 and Figure 6, and Figure 8 were only to demonstrate the process for selecting a covalent docking tool with a specific data set, not for generally ranking the docking tools.

### 3.1. Case Studies of Abnormal Docking Results

Usually, the methods of Best Scored Pose and Bets Sampled Pose produce consistent results, however, they can produce extraordinarily different results. Here, we discuss two case studies.

#### 3.1.1. Case Study 1

**Figure 7:**
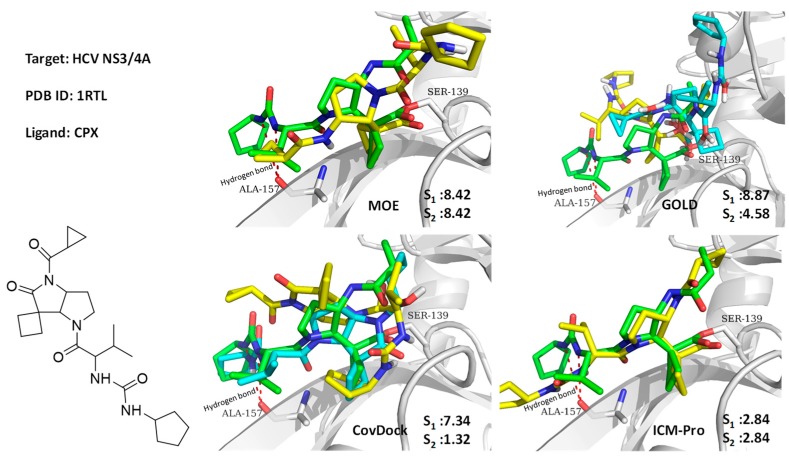
Ligand CPX covalently docked into NS3/4A protease (1RTL) with the four docking tools. Yellow: Best Scored Pose; Cyan: Best Sampled Pose; Green: Native Ligand Pose. S_1_ is the RMSD between the native pose and the Best Score Pose; S_2_ is the RMSD between the native pose and the Best Sampled Pose.

A hepatitis C virus NS3/4A protease co-crystal structure (PDB code: 1RTL) [35] has a ligand covalently bound at Ser139. The four docking tools produced poor docking results, with the exception of CovDock with its Best Sampled Pose method (1.32Å). We found that only CovDock generated the ligand conformation that was closest to the crystallographic ligand pose. This pose helped two urea NHs of the ligand to form two hydrogen bonds at Ala157, which is critical for the ligand’s pyrrolidine-5, 5-trans-lactam to open for covalent binding with Ser139. As shown in Figure 7, only CovDock recognized this near-native conformation in Best Scored Pose. As indicated in Figure 7, only CovDock generated a Best Scored Pose, in which the urea NHs are close to Ala157, and maintained the correct pose to bind covalently to Ser139. Other poses generated by MOE, GOLD, and ICM-Pro failed to keep the urea group close to Ala157 and form the critical hydrogen bonds. This case study confirms that one should consider both *Best Score Pose* and Best Sampled Pose measurements while evaluating the docking tools. In this case, even the *Best Score Pose* measurement failed, but the correct answer can still be reached by checking the Best Sampled Pose measurement.

#### 3.1.2. Case Study 2

**Figure 8:**
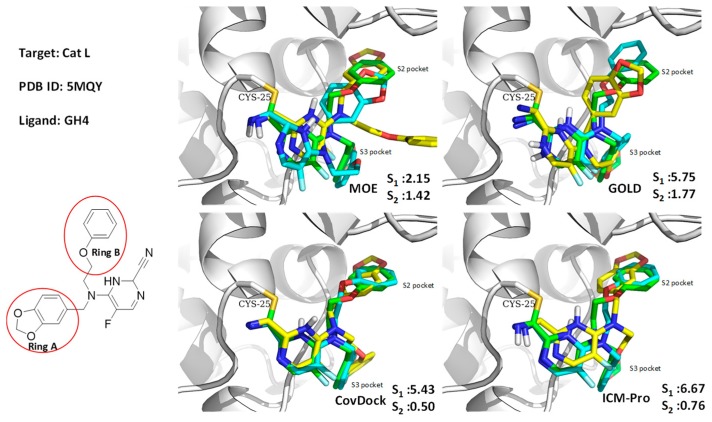
GH4 covalently docked into Cathepsin L (5MQY) with the four docking tools. Yellow: Best Scored Pose; Cyan: Best Sampled Pose; Green: Native Ligand Pose. S_1_ is the RMSD between the native pose and the Best Score Pose; S_2_ is the RMSD between the native pose and the Best Sampled Pose.

As shown in Figure 8, GOLD, CovDock, and ICM-Pro have significant deviations between Best Scored Pose and Best Sampled Pose measurements when docking GH4, the ligand of Cathepsin L, into the co-crystal structure (PDB code: 5MQY) [36]. They all produce correct binding poses using the Best Sampled Pose measurement but failed with the Best Scored Pose measurement. MOE, however, was able to generate correct poses with both measurements. Further analysis indicated that the failed poses were due to the two rings (rings A and B) at the tertiary of the fluoropyrimidine nitrile that exchange the sub-binding pockets. The corrected docking poses should be ring-A for S3, and ring-B for S2. Only MOE docked the rings into the correct sub-binding pockets with both measurements. Although the favorable interactions of the ring-B in the aromatic cleft of the S2 pocket were identified in the docking position, the pose was not selected as the highest scoring conformation due to the potential hydrophobic collapse. Earlier results in the Cambridge Structural Database (CSD) showed that the ring-B attached through the unsubstituted propyl linker accounted for 27% of the collapsed conformation in all the crystal structures with this motif. As the ligand needs to bind to hCatL in an extended conformation, an energy penalty is involved in disrupting the collapsed conformation.

### 3.2. Binding Pocket Features and Docking Performance

Specific structural features of a binding site, such as the location of an anchor residue, binding pocket size, and the flexibility of the binding pocket can be important for covalent docking. For example, there are 61 covalent kinase inhibitors (CKIs) in the BCDE data set (see Table S16).

**Figure 9:**
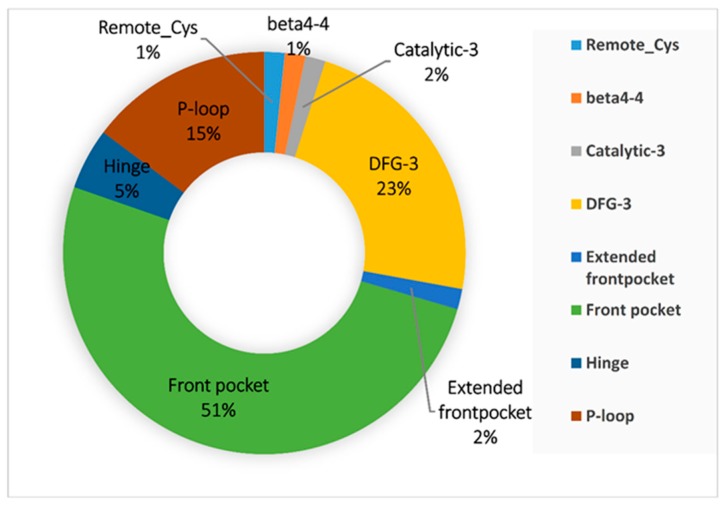
The distribution of kinase domains in the data set.

Cysteine is an anchor residue for kinases. This covalent anchor can be found in as many as eight domains: Remote-Cys, Beta4-4, Catalytic-3, Hinge, DFG-3, Extended front pocket, Front pocket, and P-loop. The possibility of the anchor residing at one particular domain, however, can vary. Based on our BCDE data set, the distribution of cysteine across kinase domains is depicted in Figure 9. The top three possible domains in which the anchor resides are Front pocket (31), DFG-3 (14), and P-loop (9). These domains offer greater possibilities for a cysteine to be used for covalent inhibitor design [37].

**Figure 10:**
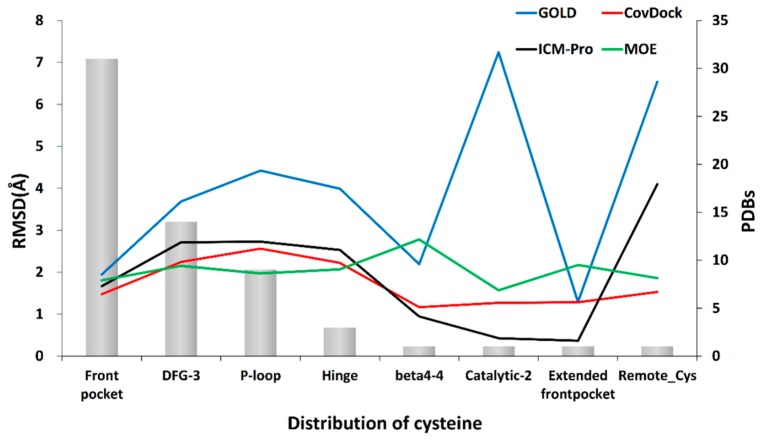
Impact of the position of reactive cysteine residue on the docking performance.

The front pocket is exposed to a polar environment suitable for the reaction and provides sufficient open space for the movement of the nucleophilic group. In contrast, the hinge region is not easily accessed by an electrophilic group of a ligand due to steric effects. The ligand has difficulty in accessing the thiol group of the cysteine, and it is hard for a covalent reaction to take place. Our results demonstrate that a docking tool can have better performance in the front pocket than in the hinge area (Figure 10). This observation is consistent with the work reported by Zhao et al. [38].

The cysteine near the DFG-3 domain is close to the polar area, while in the P-loop, it is the β carbon of the side chain and the thiol of cysteine that mainly contribute to the hydrophobic interaction and then help with the covalent attachment to a ligand. All docking tools have poorer performances in DFG, P-loop, and hinge domains. All tools have unstable performances at other domains, and this may be due to the lack of enough co-crystal structures for the evaluation.

**Figure 11:**
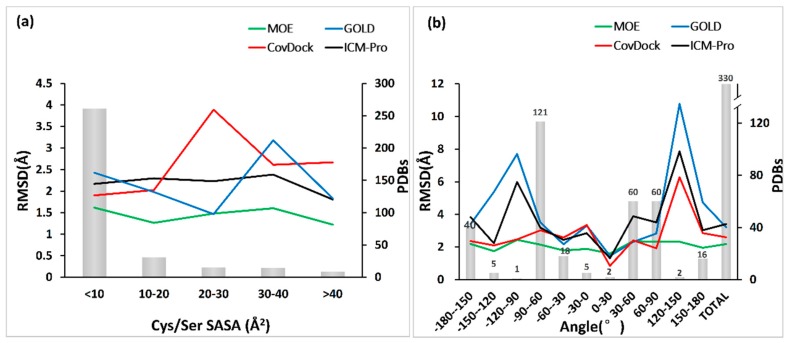
The relations of the docking accuracy and the solvent accessible surface area (SASA) or the dihedral angle. (**a**) Relation of the SASA of reactive residue and docking accuracy. SASA was calculated with GETAREA [40] (http://curie.utmb.edu/getarea.html). (**b**) The relation of the dihedral angle and the docking accuracy. The dihedral angle of the reactive residue was calculated with R package Bio3D [41].

The flexibility of the domain hosting an anchor residue, in this case, cysteine or serine, affects the docking performance. The flexibility can be measured by the solvent accessible surface area (SASA) and the dihedral angle of N–CA–CB–SG/OG [39]. The relations of the docking accuracy (measured in Best Scored Pose) and SASA and the dihedral angle are depicted in Figure 11a,b.

From Figure 11a, we can observe that all docking tools can produce the ligand poses within acceptable accuracies (RMSD ranging from 2.5 to 1.5 Å) when SASA is less than 10 Å^2^, meaning the anchor residue is less flexible. However, the performances of docking tools will behave differently when the flexibility of the anchor residue increases. The performances of CovDock and GOLD fluctuate significantly, but MOE and ICM-Pro are not significantly disturbed by the SASA increase.

In contrast, the dihedral angle can change the performance of all the docking tools except MOE, as shown in Figure 11b. Interestingly, both MOE and CovDock perform well at −180°–150°. All tools have poor results at −120°–90° and 120°–150°, which may be due to the absence of evaluation data.

## 4. Materials and Methods

### 4.1. Benchmark Data for Covalent Docking Evaluation (BCDE)

The data curation process used for BCDE was as follows: (1) 191 UniportIDs for covalent binding targets were derived from the open literature and the cBindingDB database [42], (2) the UniportIDs were used to query the RCSB PDB database, resulting in 6,584 PDB entries on 20 December 2017; (3) these PDB entries were filtered, and only co-crystal complexes with resolutions <3.0 Å were retained; (4) these co-crystal complexes were then examined for ligands non-covalently bound to their protein targets, and buffers, components, cofactors, metals, and saccharides were excluded. As a result, the number of remaining co-crystal structures was 2,235, and these were further processed using the following filters:

- DNA complexes excluded
- proteins that have covalent bindings with ligands at cysteine and serine residues included
- ligand and receptor connected with a single bond included
- co-factors within 8 Å to the ligand excluded
- ligands with at least 5 non-hydrogen atoms and less than 30 rotatable bonds were included

**Figure 12:**
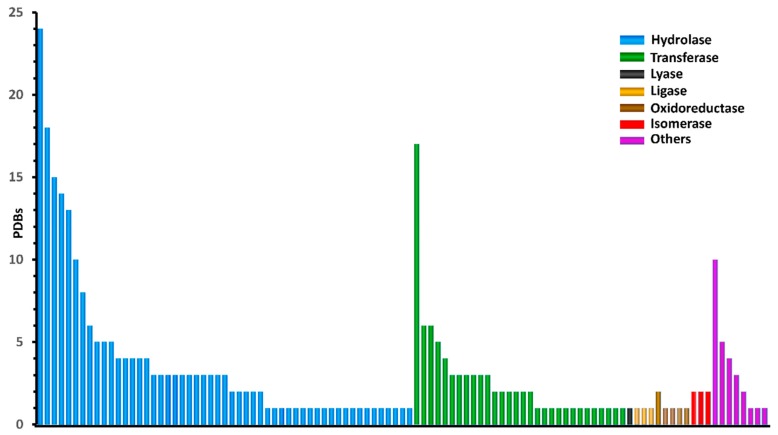
The distribution of protein types in the BCDE data set. The proteins are grouped by EC (Enzyme Commission) classification codes. All the uniprotIDs for the 330 proteins are listed in Table S1.

The BCDE data set obtained according to these criteria consisted of 330 qualified protein–ligand complexes, which contain diverse warhead species and binding geometries. The 330 structurally unique ligands covalently targeted 104 proteins (Figure 12).

To ensure the BCDE data quality, we inspected every co-crystal structure complex with Discovery Studio 4.5. For non-monomer complexes, we retained only those with one chain with covalent linkages. Water molecules, metals, co-factors, and crystallization agents were removed from target structures. In order to prevent any bias from the use of coordinates present in the protein–ligand X-ray crystal structures, all the ligands were derived from the complex and restored to the structure before the covalent bond was formed (this is a re-docking process). The ligands were saved in different files in MOL/MOL2/SDF formats. To make a covalent docking, this tool objectively docks the ligands back to the targets, and the original coordinates of the ligands are zeroed. The entire data set is available at http://www.rcdd.org.cn/cbinderdb/.

The programs with functionalities that could support covalent docking are restricted to sampling ligand conformations under the constraint provided by a predefined bond between a ligand and a protein. Since the warhead chemistry associated with the covalent ligands involves different reaction energetics and product geometries, the chemical moieties of the ligands in the BCDE data were individually inspected and corrected when necessary.

### 4.2. Criteria for Evaluating Covalent Binding Tools

Criteria were established to evaluate the docking performance of covalent docking tools, and three aspects of each docking tool, “precision”, “generality”, and “robustness” were considered. Each metric here corresponds to a typical practical application of docking tools, e.g., reproducing co-crystallized conformation with precision, or covering targets and reactions as much as possible. The concept of each metric is explained below.

- **Precision**. This refers to the capacity of a covalent docking tool to reproduce the experimental binding mode, which is represented by the RMSD value of a calculated binding conformation and experimental data. The prediction accuracy was expressed in terms of the RMSD values of the ligand heavy atoms from the conformation co-crystallized in the PDB entry.
- **Generality**. This refers to the capacity of a covalent docking tool to encompass different covalent binding systems. It is a measure of the overall precision across protein targets and reactions of all kinds. We marked the minimum RMSD among these tools for a single object then counted the number of marks in each tool. The goal was to find a covalent docking tool that is sufficiently general to be able to cope with most covalent systems.
- **Robustness**. This refers to the consistency of a covalent docking tool with the same docking process. Notwithstanding different structures of the same target or different targets of the same family or different families, there should be no unreasonable errors in the docking results. We calculated the average and deviation of the RMSD given by each docking tool in the whole data set.

### 4.3. Protocol for Selecting Tools

**Figure 13:**
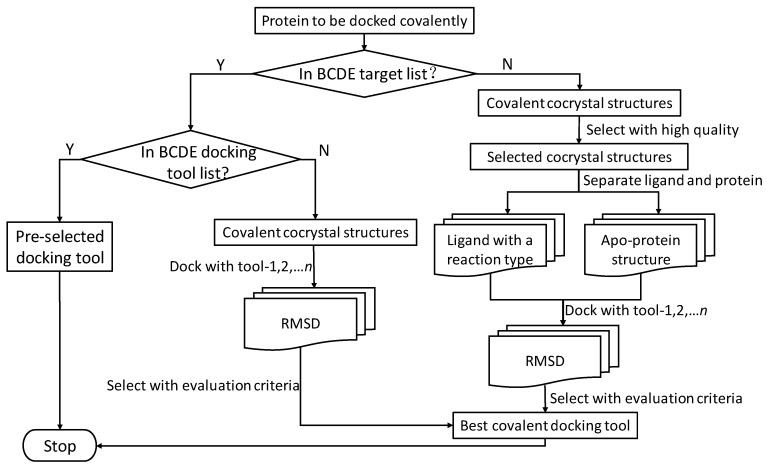
Flowchart for selecting covalent docking tools.

There should be an instructive protocol to select the most appropriate covalent docking tool. We created a flow chart (Figure 13) that helps researchers choose tools. First, it was necessary to generate our own standard data set. To do this, we had to collect all PDB files for our own covalent system, dispose of proteins and ligands with specialized software and, if necessary, prepare a defined reaction file according to the warhead reaction. Second, we examined the docking with candidate covalent docking tools. Finally, we compared and evaluated the results using measurement criteria and selected the most appropriate tool for subsequent research. This is a guide to selection of covalent docking tools.

### 4.4. Docking Programs and Scoring Functions

In this study, we utilized the docking programs MOE, GOLD, Schrodinger, and ICM-Pro for an evaluation of their ability to reproduce the co-crystallized covalent binding mode. In addition to GOLD’s implemented scoring functions (GoldScore), it has been optimized for the prediction of binding positions, which is consistent with our purpose; the other systems use a default scoring function.

MOE version 2016.08 was licensed from Chemical Computing Group ULC. The DOCK module of MOE (Molecular Operating Environment) [43] achieves conformational sampling by Placement methodology. The Placement phase of Dock generates poses from ligand conformations. Specifically, poses are generated by the reaction or transformation methodology of the Combinatorial Library. We customized reactions for those not included in the predefined reaction library using MarvinSketch [44]. The complex PDB file, which represents the receptor, was prepared using default parameters. The SDF file can help users to repair the pre-reaction structure of the ligand. The GBVI/WSA ΔG is a force field-based scoring function [45] that estimates the free energy of binding of the ligand from a given pose. Ligand poses were ranked according their binding free energy.

GOLD (Genetic Optimization for Ligand Docking) version 1.8.2 was licensed from Cambridge Crystallographic Data Centre, Cambridge, UK. It is a genetic algorithm for docking flexible ligands into protein binding sites. It is one of the first and most widely applied programs that uses docking with a preformed bond [46,47]. The program assumes that there is just one atom linking the ligand to the protein, which in this project was either S or O. Protein and ligand files were set up with the link atom identified, and appear in both the protein or ligand input files. The complex PDB file and Mol2 file, which present receptor and ligand, respectively, were prepared with default parameters. Then, the link atom in the ligand was forced to attach to the link atom in the protein. The correct geometry of the bond between ligand and protein was ensured with an angle-bending potential from the Tripos Force Field [48] added to the fitness function. On evaluating the score for the docked ligand, the angle-bending energy for the link atom was included in the calculation of the fitness score, which is the negative of the sum of the component energy terms. As a consequence, larger fitness scores are better.

CovDock was is included in Maestro version 2018.2, which was licensed from Schrodinger. LLC. CovDock is a workflow developed by Schrödinger for pose prediction and scoring of covalently bound ligands. It was built upon a foundation of the well-tested Glide [49] docking algorithm and Prime [50] structure refinement methodology for accurate prediction of non-covalently docked poses. There are two protocols for covalent docking. “Pose Prediction” mode [44], although time-consuming, is suitable for lead-optimization projects. Due largely to reduced sampling, the “CovDock-VS” mode [51] is 10–40 times faster than the “Pose Prediction” mode. According to our research purpose, the “Pose Prediction” mode was selected. The complex PDB file and SDF file that, respectively, present the receptor and the ligand, were prepared using default parameters. The ligand functional group was represented by a SMARTS pattern [51] whose atomic index identifies the atom that forms a bond with the acceptor. When the ligands were docked, all modes were tested to match the ligand. A unique match on a single ligand was considered a reasonable match and a covalent bond was formed. Affinity score was calculated as the average between the GlideScore for the pre-reaction noncovalent docking pose and the in-place score for the post-reaction pose. All docking projects were completed on the Tianhe Starlight platform of the NSCC-GZ (National Supercomputer Center in Guangzhou).

ICM-Pro version 3.8 was licensed from MolSoft LLC. Molsoft LLC developed an efficient methodology, referred to as ICM [52], which employs a Monte Carlo minimization algorithm and is based on a ligand description in internal coordinates. At first, according to the reaction type (RXN files in our data set), the warhead of the ligand was linked with the side-chain of cysteine or serine. During the conformational sampling process, the method can make a random step in a Monte Carlo procedure when armed with knowledge of the side-chain torsion angles. This biased probability Monte Carlo (BPMC) procedure [53] randomly selects the subspace first, then takes a step to a new random position independent of the previous position. All the stereoisomers in the chemical table are docked and evaluated by the ICM scoring function [54], and docking solutions are ranked by the score, which is usually a function of different energy terms based on a force-field. The lower the ICM score, the higher the chance the ligand can bind successfully.

In general, for the same protein, the calculation time increases as the number of ligand stereoisomers increases, so the number of stereoisomers was arbitrarily limited to 10. We choose the final 10 docking poses for further analysis.

## 5. Conclusions

In this study, we report a standard test data set BCDE, which fills a gap in the collection of benchmark data sets for assessing the performance of covalent docking tools. We also propose three quantitative measures (precision, generality, and robustness) for validating a covalent docking tool for protein—ligand covalent binding studies. Four profiling parameters were suggested for cross-validating covalent docking tools. Parameters such as the size and flexibility of the protein pocket, the electrophilic warhead, the chemical reactions, and the physical and chemical properties of a molecule impacting on covalent docking performance have been investigated. The performance of an ideal covalent docking tool should be independent of receptor class, warhead type, and reaction mechanisms. Our recommendations for selecting a covalent binding tool are based on the precision, generality, and robustness measures from validating the docking suites with a standard data set.

This work was based on an unevenly distributed data set in terms of receptor classifications, warhead types, and reaction mechanisms. For example, cysteine-related covalent binding data are abundantly reported but other types of covalent bindings are inadequately reported. The BCDE data should be updated from time to time to be more useful in covalent binding studies.

## Figures and Tables

**Table 1:** Covalent binding reaction classification and warhead classes.

Reaction Class	Reaction Type	Warhead Class	Warhead Core	Example	Frequency *
Nucleophilic Addition	Nucleophilic Addition by Cys	Nitrile(cys)	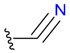	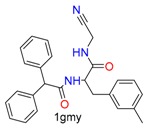	42
		Alkene(cys)	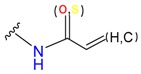	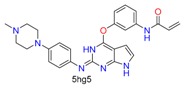	84
			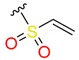	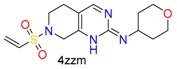	18
			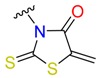	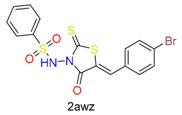	15
		Carbonyl(cys)	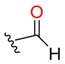	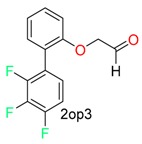	13
			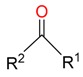	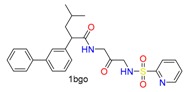	9
			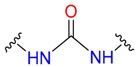	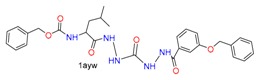	3
		Alkyne	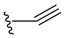	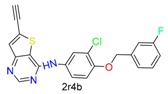	1
		Guanyl	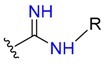	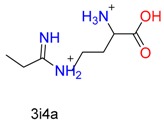	2
	Nucleophilic Addition by Ser	Nitrile (ser)	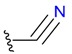	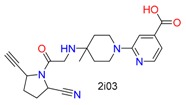	3
		Carbonyl (ser)	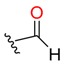	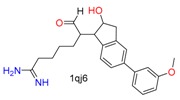	3
			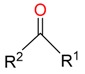	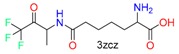	26
		Boric acid	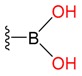	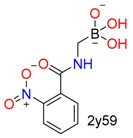	8
Nucleophilic Substitution	Nucleophilic Substitution (cys)	Halide	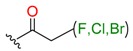	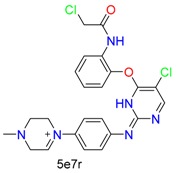	13
			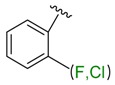	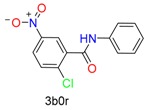	2
		Others	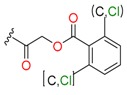	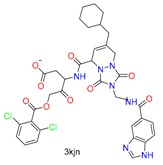	13
			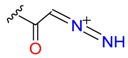	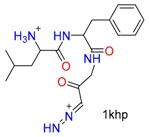	3
	Nucleophilic Substitution by Ser	Phosphonyl	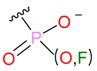	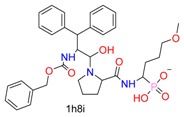	3
Ring Opening	Ring Opening by Cys	Heterocyclic	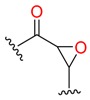	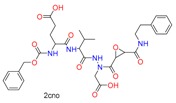	11
			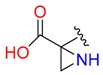	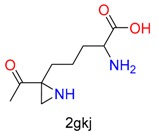	6
	Ring Opening by Ser	Lactam	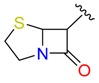	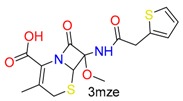	39
			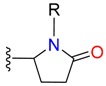	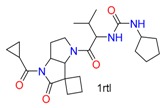	1
		Lactone	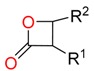	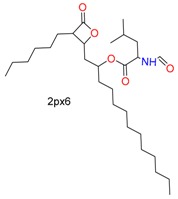	2
Disulfide Formation	Disulfide Formation	Sulfydryl	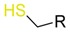	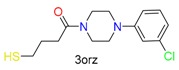	10

* Number of co-crystal structures collected in the benchmark data for covalent docking evaluation (BCDE) data set.

**Table 2:** Covalent docking performance measured by both Best Scored Pose and Best Sampled Pose based on reaction and warhead classifications. The RMSD data in the table are the backbone RMSD median values for a given warhead and a given tool. The RMSD data points are depicted in heat maps. The best RMSD poses (<1 Å) are encoded in deep green, and the worst RMSD poses (>5 Å) are encoded in deep red. *n* represents the total number of complexes for a given warhead.

Reaction Class	Warhead	*n*	Best Scored Pose (RMSD)				Best Sampled Pose (RMSD)				
			MOE	GOLD	CovDock	ICM-Pro	MOE	GOLD	CovDock	ICM-Pro	
**Nucleophilic Addition by Cys**	Nitrile	42	2.26	1.1	1.27	3.74	1.6	0.91	1.24	1.56	5 Å 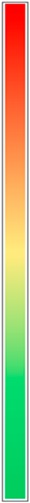 1 Å
	Alkene	117	1.99	2.47	1.47	1.68	1.4	1.59	1.18	1.41	
	Carbonyl	25	2.27	2.12	2.05	3.72	1.5	1.77	1.33	1.52	
	Alkyne	1	2.01	0.67	0.42	0.39	1.9	0.67	0.35	0.39	
	Guanyl	2	1.78	1.46	0.81	1.37	1.4	0.98	0.81	1.37	
**Nucleophilic Substitution by Cys**	Halide	15	1.67	2.31	4.37	1.06	1.3	2.17	4.37	0.88	
	Others	16	2.1	2.19	2.12	2.09	1.6	1.47	1.3	1.77	
**Ring Opening by Cys**	Heterocyclic	17	1.71	1.74	1.87	4.88	1.4	1.48	1.33	4.72	
**Disulfide Formation**	Sulfydryl	10	1.94	2.94	2.52	2.92	1.3	2.47	2.52	1.95	
**Nucleophilic Addition by Ser**	Nitrile	3	1.38	2.54	0.73	2.66	1.3	1.77	0.52	2.66	
	Carbonyl	29	1.61	3.54	3.1	2.03	1.3	1.58	1.4	1.36	
	Boronic Acid	8	1.94	2.57	2.94	2.44	1.5	1.88	2.74	2.44	
**Nucleophilic Substitution by Ser**	Phosphonyl	3	2.32	2.33	5.81	1.68	1.5	2.08	3.25	1.68	
**Ring Opening by Ser**	Lactam	40	1.73	2.95	1.7	2.53	1.5	1.62	1.7	1.48	
	Lactone	2	2.09	2.56	2.52	5.66	1.5	1.6	1.6	4.98	
***P*(*i*,*x*)**			**1.94**	**2.33**	**2.05**	**2.53**	**1.5**	**1.6**	**1.33**	**1.56**	

**Table 3:** Covalent docking performance as measured by both Best Scored Pose and Best Sampled Pose based on receptor types. The RMSD data in the table are the backbone RMSD median values for a given warhead and a given tool. The RMSD data points are depicted in heat maps. The best RMSD poses (<1 Å) are encoded in deep green, and the worst RMSD poses (>5 Å) are encoded in deep red. n represents the total number of complexes for a given warhead.

Receptor Type	*n*	Best Scored Pose (RMSD)				Best Sampled Pose (RMSD)				
		MOE	GOLD	CovDock	ICM-Pro	MOE	GOLD	CovDock	ICM-Pro	
**Hydrolase**	204	2.07	2.33	1.71	2.76	1.54	1.51	1.39	1.61	5 Å 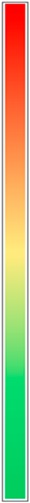 1 Å
**Transferase**	83	1.8	2.24	1.3	1.34	1.34	1.46	1.06	1.24	
**Ligase**	3	2.9	2.63	1.08	1.41	1.25	1.04	0.84	1.41	
**Lyase**	1	0.96	1.23	1.24	1.34	0.9	1.17	1.19	1.34	
**Oxidoreductase**	6	1.7	1.44	2.37	1.13	1.18	1.06	2.37	1.13	
**Isomerase**	6	1.49	1.2	1.02	2.25	1.33	0.78	1.01	2.25	
**Transcription**	18	2.38	6.15	2.15	3.42	1.6	3.03	1.71	2.1	
**Viral Protein**	4	3.91	0.81	4.29	4.93	2.97	0.81	1.3	4.93	
**Metal binding protein**	5	1.72	5.54	5.43	2.37	1.04	4.54	3	1.67	
***P*(*i*,*x*)**		**1.8**	**2.24**	**1.71**	**2.25**	**1.33**	**1.17**	**1.3**	**1.61**	

## Supplementary Materials

The following are available online, Table S1: PDB codes, uniprotIDs, information and RMSD values for complexes included in the Cov, Figure S1: Some computional costs in this work, Figure S2: Median RMSD value of Best Scored Pose on each target, Table S2: The number of best RMSD value cross all the docking tools for a given tool based on the receptor type for Best Scored Pose, Table S3: The generalities calculated by the generality(G(i,x)) formula for four docking tool with Best Scored Pose measurement based on the receptor type, Figure S3: Median RMSD value of Best Sampled Pose on each target, Table S4: The number of best RMSD value cross all the docking tools for a given tool based on the receptor type for Best Sampled Pose, Table S5: The generalities calculated by the generality(G(i,x)) formula for four docking tool with Best Sampled Pose measurement based on the receptor type, Table S6: The number of best RMSD value cross all the docking tools for a given tool based on the warhead type for Best Scored Pose, Table S7: The generalities calculated by the generality(G(i,x)) formula for four docking tool with Best Scored Pose measurement based on the warhead type, Table S8: The number of best RMSD value cross all the docking tools for a given tool based on the warhead type for Best Sampled Pose, Table S9: The generalities calculated by the generality(G(i,x)) formula for four docking tool with Best Sampled Pose measurement based on the warhead type, Table S10: Mann-Whitney U-test results of the difference between the Best Scored Pose and Best Sampled Pose RMSD sets on each of four docking tools, Table S11: Mann-Whitney U-test results of the difference between RMSD sets of the docking tools on Best Scored Pose and Best Sampled Pose on measurement, Table S12: The robustness analysis results for the four docking tools, Table S13: The P-deviations, S-counts, and F-counts for docking tools, Table S14: The P-deviations distributions for all docking tools, Table S15: Parameters to profile a docking tool, Table S16: Kinase domain distribution in BCDE and their PDB id.
