# Supplementary material for: Systematic Studies on the Protocol and Criteria for Selecting a Covalent Docking Tool
Source: Molecules. 2019 Jun 10;24(11):2183. doi: 10.3390/molecules24112183 (PMC6600387; doi:10.3390/molecules24112183)
Supplement: Supplementary file 1 [file molecules-24-02183-s001.pdf]

Systematic Studies on the Protocol and Criteria for Selecting a Covalent Docking Tool

Chang Wen [1], Xin Yan [1], Qiong Gu [1], Jiewen Du [1], Di Wu [2], Yutong Lu [2], Huihao Zhou [1,*] and Jun Xu [1,*]

[1] Research Center for Drug Discovery, School of Pharmaceutical Sciences, Sun Yat-Sen University, 132 East Circle at University City, Guangzhou 510006, China; wench7@mail2.sysu.edu.cn (C.W.); yanx28@mail.sysu.edu.cn(X.Y.); guqiong@mail.sysu.edu.cn (Q.G.); jiewen.du@xtalpi.com (J.D.)

[2] National Supercomputer Center in Guangzhou & School of Data and Computer Science, Sun Yat-Sen University, 132 East Circle at University City, Guangzhou 510006, China; wudi27@mail.sysu.edu.cn (D.W.); yutong.lu@nscc-gz.cn (Y.L.)

* Correspondence: zhuihao@mail.sysu.edu.cn (H.Z.).; junxu@biochemomes.com (J.X.)

Supplementary material

Table S1. PDB codes, uniprotIDs, information and RMSD values for complexes included in the Cov.S1 means the best scored pose, S2 represent the best sampled pose and S3 means the maximal deviated pose.

PDB	Classification	UniportID	Target	Ind.Cha.Res	Reaction	Warhead	Ligand	RMSD											
								MOE			GOLD			CovDock			ICM-Pro		
								S1	S2	S3	S1	S2	S3	S1	S2	S3	S1	S2	S3
3s3q	Hydrolase	Q8MNY2	CB1.1	100.A.CYS	Addition by Cys	Alkene(cys)	C1P	1.60	1.52	1.68	3.52	3.52	9.14	0.81	0.81	10.22	0.53	0.53	8.43
5acb	Transferase	O75909	CCNK	1039.C.CYS	Addition by Cys	Alkene(cys)	5I1	1.86	1.78	2.11	6.54	2.61	10.25	1.53	0.93	2.61	4.10	2.65	4.1
4wx6	Hydrolase	B9A5C1	L3	122.A.CYS	Addition by Cys	Alkene(cys)	3VK	3.54	2.25	4.12	1.09	1.09	3.49	1.81	0.83	1.85	2.93	1.62	5.68
4us1	Hydrolase	P01112	HRAS	118.R.CYS	Addition by Cys	Alkene(cys)	L71	1.62	1.62	2.19	5.72	5.72	5.81	5.97	1.65	6.77	5.81	1.86	5.81
4us2	Hydrolase	P01112	HRAS	118.R.CYS	Addition by Cys	Alkene(cys)	L7S	1.95	1.60	1.95	1.20	0.99	1.24	1.24	1.24	6.19	2.56	1.50	6.11
4lyf	Hydrolase	P01116	KRAS	12.B.CYS	Addition by Cys	Alkene(cys)	21C	2.15	1.18	4.24	2.08	0.68	3.04	2.45	1.30	2.52	1.17	1.17	3.35
4lyj	Hydrolase	P01116	KRAS	12.A.CYS	Addition by Cys	Alkene(cys)	21F	2.39	1.41	2.69	3.61	1.50	7.98	0.91	0.91	1.92	2.44	2.44	6.14
4m1o	Hydrolase	P01116	KRAS	12.B.CYS	Addition by Cys	Alkene(cys)	21J	1.15	1.15	1.16	1.24	0.63	1.29	1.20	1.20	2.11	0.79	0.73	3.49
4m1s	Hydrolase	P01116	KRAS	12.B.CYS	Addition by Cys	Alkene(cys)	21K	1.13	1.13	1.99	0.46	0.46	1.12	1.64	1.64	2.53	0.67	0.65	6.71
4m1t	Hydrolase	P01116	KRAS	12.B.CYS	Addition by Cys	Alkene(cys)	21M	2.14	2.14	2.77	0.45	0.32	0.74	1.50	1.50	2.33	1.17	0.74	3.4
4m1w	Hydrolase	P01116	KRAS	12.C.CYS	Addition by Cys	Alkene(cys)	21R	5.07	3.40	5.07	9.19	9.13	9.61	13.93	13.93	13.9	10.93	10.22	12.4
4m21	Hydrolase	P01116	KRAS	12.B.CYS	Addition by Cys	Alkene(cys)	21Y	1.37	0.77	1.85	3.62	1.05	3.93	1.69	1.69	1.79	3.45	3.45	7.41
4m22	Hydrolase	P01116	KRAS	12.C.CYS	Addition by Cys	Alkene(cys)	22C	2.11	1.11	2.43	3.54	2.42	3.54	2.49	2.49	3.58	12.37	2.74	12.37
5f2e	Hydrolase	P01116	KRAS	12.A.CYS	Addition by Cys	Alkene(cys)	5UT	2.50	1.24	2.51	1.47	1.01	5.79	2.12	1.09	2.25	0.62	0.62	3.26
5v6s	Hydrolase	P01116	KRAS	12.A.CYS	Addition by Cys	Alkene(cys)	8YD	2.69	1.14	2.69	4.10	3.51	3.60	1.07	1.02	2.12	0.28	0.28	2.07
5v71	Hydrolase	P01116	KRAS	12.A.CYS	Addition by Cys	Alkene(cys)	8ZG	2.21	1.53	2.96	4.06	2.14	7.37	1.34	1.34	2.3	2.42	1.64	2.46
5v9o	Hydrolase	P01116	KRAS	12.A.CYS	Addition by Cys	Alkene(cys)	91G	1.51	1.14	1.51	1.15	1.15	7.23	0.96	0.86	1.92	10.23	10.23	2.36
5e93	Oxidoreductase	Q4D3W2	PYR4	130.A.CYS	Addition by Cys	Alkene(cys)	5LL	1.55	1.28	1.66	0.47	0.43	1.05	1.76	1.18	2.68	1.00	1.00	3.03
5ea9	Oxidoreductase	Q4D3W2	PYR4	130.A.CYS	Addition by Cys	Alkene(cys)	5LM	2.23	1.85	2.54	1.21	1.07	1.21	6.46	6.46	6.46	0.45	0.45	2.36
5fx6	Hydrolase	P04936	POLG	147.A.CYS	Addition by Cys	Alkene(cys)	6OY	1.68	1.68	4.55	4.27	4.27	1.64	0.90	0.90	2.36	1.01	1.01	3.56
1cqq	Hydrolase	P04936	POLG	147.A.CYS	Addition by Cys	Alkene(cys)	AG7	2.48	1.54	2.55	1.10	1.10	4.04	0.90	0.68	6.77	4.46	2.21	5.96
5fx5	Hydrolase	P04936	POLG	147.A.CYS	Addition by Cys	Alkene(cys)	HOV	1.37	1.37	3.08	1.42	1.35	7.91	0.91	0.91	5.03	0.43	0.43	3.46
5iyt	Hydrolase	O91734	POLG	147.B.CYS	Addition by Cys	Alkene(cys)	NZN	2.70	2.70	5.6	4.35	4.35	6.75	8.03	6.33	8.03	6.04	4.24	6.77
5daf	Transcription	Q14145	KEAP1	151.A.CYS	Addition by Cys	Alkene(cys)	58E	2.04	1.52	2.04	3.13	3.13	8.59	1.20	0.95	2.2	2.56	2.56	3.23
5dad	Transcription	Q14145	KEAP1	151.A.CYS	Addition by Cys	Alkene(cys)	TX6	1.36	1.10	2.83	0.87	0.84	1.03	0.27	0.27	1.27	5.47	3.41	5.74
5git	Transcription	Q14145	KEAP1	151.A.CYS	Addition by Cys	Alkene(cys)	XXT	3.06	2.54	3.18	2.47	2.40	2.47	2.23	1.66	2.35	3.53	2.66	3.53
3v6r	Transferase	P53779	MAPK10	154.A.CYS	Addition by Cys	Alkene(cys)	CQQ	2.02	1.76	3.17	3.49	1.90	5.01	1.72	1.57	2.72	2.35	2.35	4.63
2c1e	Hydrolase	P42574	CASP3	163.A.CYS	Addition by Cys	Alkene(cys)	Peptide	2.21	1.79	5.85	4.07	3.64	8.91	2.53	1.95	2.93	5.89	2.55	5.86
2c2k	Hydrolase	P42574	CASP3	163.A.CYS	Addition by Cys	Alkene(cys)	Peptide	2.56	2.56	3.56	5.60	3.94	8.87	3.84	1.68	2.25	3.24	2.26	3.24
2c2o	Hydrolase	P42574	CASP3	163.A.CYS	Addition by Cys	Alkene(cys)	Peptide	3.15	2.99	3.15	2.68	1.97	6.95	2.43	1.98	2.42	2.20	2.20	3
5lcj	Transferase	P28482	MAPK1	166.A.CYS	Addition by Cys	Alkene(cys)	6TS	2.28	2.28	2.45	5.73	5.64	12.49	4.61	4.61	4.61	7.74	7.74	7.75
5lck	Transferase	P28482	MAPK1	166.A.CYS	Addition by Cys	Alkene(cys)	6TT	3.11	1.34	3.29	0.83	0.83	7.41	0.21	0.21	1.21	0.79	0.79	6.79
4zzo	Transferase	P28482	MAPK1	166.A.CYS	Addition by Cys	Alkene(cys)	CQ3	1.97	1.58	3.09	1.19	0.83	1.20	0.79	0.79	1.71	1.66	1.33	3.91
4zzm	Transferase	P28482	MAPK1	166.A.CYS	Addition by Cys	Alkene(cys)	CQ6	1.97	1.16	2.18	1.94	1.16	1.95	0.49	0.49	1.52	0.82	0.82	3.75
4gs6	Transferase	Q15750	TAB1	174.A.CYS	Addition by Cys	Alkene(cys)	1FM	2.84	2.08	3.08	7.00	4.87	7.76	4.87	0.24	4.89	0.79	0.79	3.42
5j7s	Transferase	Q15750	TAB1	174.A.CYS	Addition by Cys	Alkene(cys)	6H3	4.71	1.17	4.71	1.60	1.00	5.74	1.84	0.63	2.85	1.34	1.34	5.11
5j8i	Transferase	Q15750	TAB1	174.A.CYS	Addition by Cys	Alkene(cys)	6H4	1.51	0.98	2.12	8.40	2.34	8.40	1.42	1.42	1.54	4.24	3.25	4.54
5jk3	Transferase	Q15750	TAB1	174.A.CYS	Addition by Cys	Alkene(cys)	6L4	0.99	0.99	3.57	4.80	1.65	4.80	2.22	2.22	3.21	4.54	3.63	4.54
5vqv	Transferase	P03366	GAG-POL	181.A.CYS	Addition by Cys	Alkene(cys)	9J1	1.51	1.51	2.49	1.79	1.22	2.60	0.27	0.27	1.27	4.82	3.97	4.82

5vqy	Transferase	P03366	GAG-POL	181.A.CYS	Addition by Cys	Alkene(cys)	9J1	1.45	1.34	3.02	1.15	0.82	4.47	0.23	0.23	1.23	0.62	0.62	4.68
1ju6	Transferase	P04818	TYMS	195.C.CYS	Addition by Cys	Alkene(cys)	UMP	0.71	0.71	1.86	4.85	4.55	5.80	1.03	1.03	1.96	1.05	1.05	6.78
1hvy	Transferase	P04818	TYMS	195.A.CYS	Addition by Cys	Alkene(cys)	UMP	1.70	1.42	2.06	2.37	1.81	2.57	1.11	1.11	10.43	1.24	1.24	3.78
5hze	Transferase	Q02750	MAP2K1	207.A.CYS	Addition by Cys	Alkene(cys)	E62	1.08	1.08	2.7	6.25	2.87	7.94	0.40	0.40	1.39	1.95	1.41	2.64
3szb	Oxidoreductase	P30838	ALDH3A1	243.A.CYS	Addition by Cys	Alkene(cys)	I1E	1.45	1.01	2.01	1.67	0.64	2.08	1.28	1.28	9.69	1.27	1.27	9.74
1fh0	Hydrolase	O60911	CATL2	2098.B.CYS	Addition by Cys	Alkene(cys)	0IW	1.99	1.74	2.15	1.51	1.31	7.18	8.81	2.01	2.53	1.09	1.09	10.37
4pi3	Hydrolase	P25779	CYSP	25.A.CYS	Addition by Cys	Alkene(cys)	2V5	1.56	1.56	3.07	8.08	3.43	10.65	4.77	3.57	4.78	2.06	2.06	11.91
2fye	Hydrolase	P25774	CATS	25.A.CYS	Addition by Cys	Alkene(cys)	BCQ	2.25	2.25	3.76	1.91	1.31	8.38	1.97	1.97	3.32	1.77	1.77	9.36
2g6d	Hydrolase	P25774	CATS	25.A.CYS	Addition by Cys	Alkene(cys)	MQQ	3.46	1.46	2.27	1.98	1.57	2.84	2.02	1.51	9.77	2.42	2.26	5.06
5o8u	Transferase	Q16539	MAPK14	252.A.CYS	Addition by Cys	Alkene(cys)	9O5	2.17	1.73	2.18	1.29	1.16	3.15	1.28	1.06	1.93	0.37	0.37	6.97
3s3p	Transferase	P21980	TGM2	277.A.CYS	Addition by Cys	Alkene(cys)	Peptide	2.05	2.05	2.1	13.62	1.78	13.62	1.80	1.80	10.03	6.17	1.30	6.17
3s3s	Transferase	P21980	TGM2	277.A.CYS	Addition by Cys	Alkene(cys)	Peptide	2.25	2.25	4.52	5.04	3.12	12.90	1.58	1.58	10.6	2.24	1.77	2.24
3x1i	Transcription	P37231	PPAR-γ	285.A.CYS	Addition by Cys	Alkene(cys)	66B	1.92	1.53	3.39	8.45	3.17	8.79	1.89	1.89	5.67	5.61	2.55	7.62
5azv	Transcription	P37231	PPAR-γ	285.A.CYS	Addition by Cys	Alkene(cys)	4M5	2.57	1.42	2.57	5.75	1.59	6.04	2.67	2.67	2.67	1.40	1.40	4.51
5dv6	Transferase	P37231	PPAR-γ	285.A.CYS	Addition by Cys	Alkene(cys)	B4H	0.68	0.68	1.54	4.63	4.63	6.60	1.76	1.76	2.75	7.28	0.54	7.28
2zk5	Transcription	P37231	PPAR-γ	285.A.CYS	Addition by Cys	Alkene(cys)	NRO	1.71	0.72	1.71	0.94	0.94	1.00	1.28	1.26	2.89	0.50	0.50	6.69
2zk4	Transcription	P37231	PPAR-γ	285.A.CYS	Addition by Cys	Alkene(cys)	OCR	2.32	1.13	3.05	8.32	4.42	8.60	8.50	5.40	8.58	6.60	6.60	9.4
2zk3	Transcription	P37231	PPAR-γ	285.A.CYS	Addition by Cys	Alkene(cys)	OCX	2.28	1.55	2.28	7.06	2.55	10.98	4.02	4.02	10.61	7.60	4.34	7.6
2zk1	Transcription	P37231	PPAR-γ	285.A.CYS	Addition by Cys	Alkene(cys)	PTG	2.61	1.92	2.61	8.78	6.02	9.32	8.91	3.19	8.43	8.83	4.21	10.71
2zk2	Transcription	P37231	PPAR-γ	285.A.CYS	Addition by Cys	Alkene(cys)	PTG	2.60	2.10	2.65	7.91	6.98	7.98	10.74	6.50	8.67	9.55	7.48	9.55
5dv8	Transferase	P37231	PPAR-γ	285.A.CYS	Addition by Cys	Alkene(cys)	T51	3.67	1.86	3.67	8.93	1.59	11.44	1.02	0.92	3.44	0.49	0.49	2.44
5th7	Transferase	Q9NQR1	KMT5A	311.A.CYS	Addition by Cys	Alkene(cys)	7BY	1.26	1.07	3.75	1.04	1.04	5.50	16.09	16.09	13.85	1.94	1.94	5.89
4kty	Transferase	P00488	F13A1	314.A.CYS	Addition by Cys	Alkene(cys)	Peptide	4.86	2.97	3.2	3.22	3.22	8.66	7.78	7.15	8.28	4.34	3.03	4.34
3svv	Transferase	P00523	SRC	338.A.CYS	Addition by Cys	Alkene(cys)	VSP	1.87	1.23	1.87	0.83	0.83	3.44	0.99	0.99	9.58	0.74	0.74	3.58
2hwp	Transferase	P00523	SRC	345.B.CYS	Addition by Cys	Alkene(cys)	DJK	2.54	1.79	2.54	6.86	2.88	7.05	3.95	3.95	3.9	5.69	5.69	7.82
3lok	Transferase	P00523	SRC	345.A.CYS	Addition by Cys	Alkene(cys)	DJK	0.66	0.66	1.79	5.53	2.21	5.53	0.30	0.30	1.34	3.22	1.87	3.23
2qlq	Transferase	P00523	SRC	345.A.CYS	Addition by Cys	Alkene(cys)	SR2	2.45	1.01	2.45	1.24	1.04	1.24	0.41	0.41	2.93	1.79	1.79	1.93
2qq7	Transferase	P00523	SRC	345.A.CYS	Addition by Cys	Alkene(cys)	SR2	1.40	1.29	1.8	1.01	0.86	1.01	1.13	0.86	2.37	6.97	4.49	6.97
2c2z	Hydrolase	Q14790	CASP8	360.A.CYS	Addition by Cys	Alkene(cys)	Peptide	2.80	1.64	3.53	2.04	1.97	8.17	10.04	1.38	13.07	7.33	7.33	9.81
4yrs	Ligase	Q4DA54	Q4DA54	365.A.CYS	Addition by Cys	Alkene(cys)	4JS	3.48	1.44	3.89	2.63	1.04	2.63	0.78	0.78	1.7	1.47	1.47	2.23
2ax1	Transferase	P26663	POLG	366.A.CYS	Addition by Cys	Alkene(cys)	5EE	0.81	0.77	2.65	4.12	4.12	6.74	8.68	8.01	2.99	2.44	2.42	3.1
2awz	Transferase	P26663	POLG	366.A.CYS	Addition by Cys	Alkene(cys)	5H	1.49	1.20	1.49	5.18	3.57	7.60	8.15	8.15	5.18	1.68	1.68	2.73
2ax0	Transferase	P26663	POLG	366.A.CYS	Addition by Cys	Alkene(cys)	5X	2.78	1.57	2.9	6.79	6.01	6.83	1.99	0.95	4.3	2.01	1.80	3.73
3bm8	Hydrolase	P15273	YOPH	403.A.CYS	Addition by Cys	Alkene(cys)	PSY	1.79	1.79	2.32	1.71	1.48	2.00	0.78	0.78	1.94	1.26	1.26	2.59
3blt	Hydrolase	P15273	YOPH	403.A.CYS	Addition by Cys	Alkene(cys)	PSY	1.40	1.40	2.33	1.19	1.15	1.19	0.91	0.91	1.86	1.36	1.36	4.22
3blu	Hydrolase	P15273	YOPH	403.A.CYS	Addition by Cys	Alkene(cys)	PVS	1.80	1.12	1.97	0.58	0.52	0.59	0.48	0.48	2.72	1.50	1.50	3.6
4d9u	Transferase	P51812	RPS6KA3	436.A.CYS	Addition by Cys	Alkene(cys)	0JH	0.80	0.80	1.77	8.17	7.24	10.03	1.25	1.23	2.25	1.03	1.03	5.1
4hct	Transferase	Q08881	ITK	442.A.CYS	Addition by Cys	Alkene(cys)	18R	1.42	1.42	3.91	4.29	0.55	6.05	0.61	0.61	9.16	0.44	0.44	8.14
4hcu	Transferase	Q08881	ITK	442.A.CYS	Addition by Cys	Alkene(cys)	13L	1.06	0.87	2.24	0.53	0.53	4.26	0.85	0.85	1.52	1.42	1.42	6.57
3t9t	Transferase	Q08881	ITK	442.A.CYS	Addition by Cys	Alkene(cys)	IAQ	1.10	1.10	1.25	1.00	1.00	4.11	1.02	0.85	2.12	0.58	0.58	3.98
4qqc	Transferase	P22455	FGFR4	477.A.CYS	Addition by Cys	Alkene(cys)	37O	2.39	1.41	3.39	2.24	1.79	9.79	3.12	3.12	9.04	0.70	0.70	9.81
4qq5	Transferase	P22455	FGFR4	482.A.CYS	Addition by Cys	Alkene(cys)	37O	1.64	1.64	2.82	1.37	1.09	1.37	2.18	2.12	2.18	0.31	0.31	12.15
5j87	Transferase	Q06187	BTK	481.A.CYS	Addition by Cys	Alkene(cys)	N42	1.64	1.64	3.13	0.93	0.93	6.11	1.96	1.96	2.96	4.19	2.23	4.2
3bwk	Hydrolase	Q9NAW4	Q9NAW4	51.A.CYS	Addition by Cys	Alkene(cys)	C1P	5.80	2.01	8.59	2.46	2.44	9.26	1.66	1.66	2.49	2.09	2.09	10.56
4hax	Transcription	P30822	CRM1	539.C.CYS	Addition by Cys	Alkene(cys)	RJA	4.84	1.75	4.84	5.46	5.46	11.23	1.92	1.50	2.59	3.31	1.59	3.89

4hay	Transcription	P30822	CRM1	539.C.CYS	Addition by Cys	Alkene(cys)	LM8	1.59	1.59	2.43	2.93	2.93	7.88	2.08	1.45	12.66	2.57	2.04	2.93
4haz	Transcription	P30822	CRM1	539.C.CYS	Addition by Cys	Alkene(cys)	LBF	3.24	1.83	4.87	14.83	4.60	14.83	1.11	0.49	12.92	0.93	0.93	2.78
4hav	Transcription	P30822	CRM1	539.C.CYS	Addition by Cys	Alkene(cys)	AA8	5.24	2.14	5.24	6.55	4.66	9.37	2.32	1.18	12.69	2.95	1.39	2.95
4xcu	Transferase	P22455	FGFR4	552.A.CYS	Addition by Cys	Alkene(cys)	40M	2.18	1.47	2.44	9.39	9.39	9.70	0.47	0.47	1.47	0.59	0.59	4.09
5vnd	Transferase	P11362	FGFR1	563.A.CYS	Addition by Cys	Alkene(cys)	9ES	1.57	1.42	2.07	7.25	5.29	8.86	1.27	1.05	2.27	0.43	0.43	0.53
1npz	Hydrolase	P25774	CATS	25.A.CYS	Addition by Cys	Alkene(cys)	C1P	2.49	2.14	2.89	2.34	2.16	2.36	1.47	1.47	3.99	1.55	1.55	5
4li5	Transferase	P00533	EGFR	797.A.CYS	Addition by Cys	Alkene(cys)	1WY	2.04	1.78	2.89	2.78	2.78	6.35	0.31	0.31	1.36	0.37	0.37	6.43
4lqm	Transferase	P00533	EGFR	797.A.CYS	Addition by Cys	Alkene(cys)	DJK	1.38	1.31	1.64	1.27	0.97	1.27	0.78	0.78	1.77	0.83	0.83	2.91
3ika	Transferase	P00533	EGFR	797.A.CYS	Addition by Cys	Alkene(cys)	0UN	2.59	1.09	2.59	6.82	3.44	7.93	1.87	1.87	4.67	4.51	4.51	8.79
4g5j	Transferase	P00533	EGFR	797.A.CYS	Addition by Cys	Alkene(cys)	0WN	2.20	1.47	3.37	2.15	0.74	2.39	0.68	0.68	1.89	1.31	1.31	9.15
4i24	Transferase	P00533	EGFR	797.A.CYS	Addition by Cys	Alkene(cys)	1C9	1.42	0.96	1.93	1.52	0.98	4.78	0.41	0.41	1.59	0.68	0.68	3.3
5fed	Transferase	P00533	EGFR	797.A.CYS	Addition by Cys	Alkene(cys)	5X4	1.30	1.30	2.92	4.36	3.58	7.14	0.79	0.79	7.07	7.01	6.71	7.22
5hg7	Transferase	P00533	EGFR	797.A.CYS	Addition by Cys	Alkene(cys)	630	1.66	1.34	2.15	0.36	0.32	0.38	0.58	0.58	5.02	0.99	0.99	3.16
5hg5	Transferase	P00533	EGFR	797.A.CYS	Addition by Cys	Alkene(cys)	633	1.53	1.15	2.86	0.67	0.67	8.31	1.30	0.49	7.16	2.55	2.55	9.8
5hg8	Transferase	P00533	EGFR	797.A.CYS	Addition by Cys	Alkene(cys)	634	1.94	0.99	2.06	0.41	0.41	1.12	5.20	0.37	4.75	1.61	1.61	4.34
5hg9	Transferase	P00533	EGFR	797.A.CYS	Addition by Cys	Alkene(cys)	63A	2.27	1.31	2.3	0.50	0.50	4.99	1.12	1.12	4.88	1.06	1.06	3.74
5j9y	Transferase	P00533	EGFR	797.A.CYS	Addition by Cys	Alkene(cys)	6HL	1.12	1.08	2.29	2.46	0.68	2.54	0.77	0.77	2.34	0.69	0.69	5.32
5gnk	Transferase	P00533	EGFR	797.A.CYS	Addition by Cys	Alkene(cys)	80U	1.80	1.68	2.93	0.72	0.65	1.61	0.39	0.37	1.61	0.69	0.69	3.14
5ug9	Transferase	P00533	EGFR	797.A.CYS	Addition by Cys	Alkene(cys)	8AM	2.19	1.67	2.47	0.44	0.44	0.87	0.61	0.60	2.57	0.45	0.45	6.04
5ug8	Transferase	P00533	EGFR	797.A.CYS	Addition by Cys	Alkene(cys)	8BP	2.64	1.31	3.95	1.11	0.78	1.11	0.91	0.28	1.88	0.50	0.50	5.81
5ugc	Transferase	P00533	EGFR	797.A.CYS	Addition by Cys	Alkene(cys)	8BS	3.68	0.96	3.68	5.30	5.29	5.30	0.57	0.57	2.04	0.58	0.58	5.35
3w2q	Transferase	P00533	EGFR	797.A.CYS	Addition by Cys	Alkene(cys)	HKI	1.99	1.40	2.2	1.02	0.93	3.06	2.28	1.98	2.28	1.49	1.49	2.41
4pe7	Metal binding protein	P02638	S100-B	84.A.CYS	Addition by Cys	Alkene(cys)	0DN	0.95	0.95	2.57	9.52	7.08	11.71	9.78	5.45	9.78	2.37	2.37	3.75
4pdz	Metal binding protein	P02638	S100-B	84.B.CYS	Addition by Cys	Alkene(cys)	CTI	2.76	1.81	2.89	4.54	4.54	5.05	5.84	4.90	5.85	4.76	4.76	6.88
4pe4	Metal binding protein	P02638	S100-B	84.X.CYS	Addition by Cys	Alkene(cys)	REV	2.02	0.84	2.4	6.27	6.27	6.32	2.14	2.14	2.52	3.36	1.06	5.67
3zim	Transferase	P42336	PIK3CA	862.A.CYS	Addition by Cys	Alkene(cys)	KKR	2.78	2.45	4.17	2.19	1.33	13.81	1.17	1.17	3.02	0.94	0.94	7.14
4qps	Transferase	P52333	JAK3	909.A.CYS	Addition by Cys	Alkene(cys)	37Q	1.65	1.31	1.71	0.95	0.85	0.95	0.45	0.45	2.28	0.36	0.36	5.38
4z16	Transferase	P52333	JAK3	909.A.CYS	Addition by Cys	Alkene(cys)	4LH	2.00	1.80	2.14	1.49	1.49	9.65	1.09	0.80	1.09	7.34	5.98	8.73
5toz	Transferase	P52333	JAK3	909.A.CYS	Addition by Cys	Alkene(cys)	7H4	1.72	1.21	2.75	0.83	0.73	4.33	2.51	2.33	6.22	0.66	0.66	3.42
5ttu	Transferase	P52333	JAK3	909.A.CYS	Addition by Cys	Alkene(cys)	7KV	1.92	0.77	2.31	0.63	0.63	2.28	0.99	0.74	5.07	0.56	0.56	2.9
5ttv	Transferase	P52333	JAK3	909.A.CYS	Addition by Cys	Alkene(cys)	7KX	1.48	0.98	1.7	3.47	1.60	3.85	1.58	1.44	2.68	0.49	0.49	5.21
5WFJ	Transferase	P52333	JAK3	909.A.CYS	Addition by Cys	Alkene(cys)	9Z4	1.05	1.05	2.01	4.98	1.02	5.43	0.50	0.50	3.23	4.19	1.13	4.19
5d11	Transferase	P00523	SRC	345.A.CYS	Addition by Cys	Alkene(cys)	56G	2.27	1.27	2.27	9.15	1.67	9.15	2.17	0.47	2.26	2.04	1.24	2.04
2r4b	Transferase	Q15303	ERBB4	803.A.CYS	Addition by Cys	Alkyne(cys)	GW7	2.01	1.90	2.29	0.67	0.67	1.49	0.42	0.35	1.56	0.39	0.39	6.12
2f9a	Transferase	Q9M6U3	HMGS1	117.A.CYS	Addition by Cys	Carbonyl(cys)	F24	1.51	1.51	2.67	2.12	1.77	2.12	2.60	2.02	2.46	2.39	2.39	3.46
5n19	Hydrolase	P0C6X7	REP	145.A.CYS	Addition by Cys	Carbonyl(cys)	D03	2.95	1.24	2.95	1.65	1.03	3.91	2.64	2.49	6.59	4.36	4.36	6.14
5c5o	Hydrolase	P0C6X7	REP	145.A.CYS	Addition by Cys	Carbonyl(cys)	SDJ	1.42	1.42	2.64	2.57	0.79	5.51	2.51	2.42	7.94	6.88	2.65	6.88
6ffn	Hydrolase	P04936	POLG	147.A.CYS	Addition by Cys	Carbonyl(cys)	D8K	3.35	2.37	3.35	0.73	0.64	0.73	2.83	0.78	8.38	5.64	1.87	5.64
1pyo	Hydrolase	P42574	CASP3	155.A.CYS	Addition by Cys	Carbonyl(cys)	ASJ	1.47	1.41	4.07	4.64	2.06	9.58	1.09	1.01	2.17	1.30	1.30	2.18
1nlj	Hydrolase	P43235	CATK	25.B.CYS	Addition by Cys	Carbonyl(cys)	2CA	6.34	6.23	3.64	0.61	0.61	0.99	2.68	1.14	10.02	2.95	1.33	4.58
1nl6	Hydrolase	P43235	CATK	25.X.CYS	Addition by Cys	Carbonyl(cys)	750	2.27	2.25	5.79	2.08	2.08	4.56	1.62	1.58	8.41	1.28	0.90	1.23
1bgo	Hydrolase	P43235	CATK	25.A.CYS	Addition by Cys	Carbonyl(cys)	I10	3.23	1.61	4.07	1.02	0.94	4.70	1.20	1.20	2.14	11.39	1.28	11.38
1ayw	Hydrolase	P43235	CATK	25.A.CYS	Addition by Cys	Carbonyl(cys)	IN3	1.30	1.05	3.08	5.94	3.14	12.30	4.31	4.31	13.74	13.46	12.63	13.45

1ayv	Hydrolase	P43235	CATK	25.A.CYS	Addition by Cys	Carbonyl(cys)	IN6	3.41	3.41	5.18	2.29	2.29	6.32	2.05	2.05	14.06	3.72	2.22	3.72
1ayu	Hydrolase	P43235	CATK	25.A.CYS	Addition by Cys	Carbonyl(cys)	INA	3.25	2.68	3.25	3.30	1.97	5.43	6.48	1.00	14.19	2.67	2.67	5.97
1au3	Hydrolase	P43235	CATK	25.A.CYS	Addition by Cys	Carbonyl(cys)	PCM	6.50	6.50	1.94	1.51	1.51	1.67	1.80	1.66	6.93	13.06	11.62	13.6
1au2	Hydrolase	P43235	CATK	25.A.CYS	Addition by Cys	Carbonyl(cys)	POS	3.94	2.60	3.94	3.11	3.11	13.15	2.00	2.00	12.31	9.61	3.87	9.64
4yv8	Hydrolase	P43235	CATK	25.A.CYS	Addition by Cys	Carbonyl(cys)	RGL	3.21	1.93	3.28	6.16	1.88	7.15	9.54	1.91	8.38	6.20	3.78	6.21
1au0	Hydrolase	P43235	CATK	25.A.CYS	Addition by Cys	Carbonyl(cys)	SDK	2.85	1.70	3.42	5.51	1.86	7.55	3.56	3.31	6.19	3.54	3.54	5.67
2op3	Hydrolase	P25774	CATS	25.A.CYS	Addition by Cys	Carbonyl(cys)	TF5	2.84	1.29	2.84	0.48	0.48	1.85	0.41	0.41	1.76	4.75	0.44	7.38
3ovx	Hydrolase	P25774	CATS	25.A.CYS	Addition by Cys	Carbonyl(cys)	O64	1.87	1.87	3.74	0.57	0.57	2.58	4.03	1.08	2.05	8.95	0.37	8.95
3of8	Hydrolase	P07711	CATL1	26.A.CYS	Addition by Cys	Carbonyl(cys)	I0Y	1.94	1.89	2.94	2.74	2.22	10.85	0.85	0.85	2.75	11.25	1.72	11.4
2jai	Hydrolase	O94760	DDAH1	273.A.CYS	Addition by Cys	Carbonyl(cys)	CIR	1.10	0.64	1.97	1.27	1.27	1.33	0.99	0.99	6.278	0.82	0.75	1.92
4x0t	Oxidoreductase	P49419	ALDH7A1	302.A.CYS	Addition by Cys	Carbonyl(cys)	3W9	1.36	1.36	2.29	1.05	1.05	1.12	2.17	2.17	2.26	0.90	0.90	3.53
5lbg	Transferase	I6Y9J2	IDTB	354.B.CYS	Addition by Cys	Carbonyl(cys)	3HL	3.02	3.02	3.12	1.25	1.25	1.29	1.62	1.62	2.34	0.79	0.79	7.96
1qtn	Hydrolase	Q14790	CASP8	360.A.CYS	Addition by Cys	Carbonyl(cys)	ASJ	1.58	1.36	2.83	2.85	2.85	7.77	0.93	0.93	2.68	8.68	1.49	8.68
1f1j	Hydrolase	P55210	CASP7	186.A.CYS	Addition by Cys	Carbonyl(cys)	Peptide	2.78	1.48	3.06	4.72	2.81	5.36	1.10	0.97	2.56	1.36	1.36	5.65
3bpm	Hydrolase	Q9NAW4	Q9NAW4	51.A.CYS	Addition by Cys	Carbonyl(cys)	AR7	1.72	1.39	2.15	1.39	1.39	1.46	4.41	1.33	9.56	3.18	1.52	3.18
5c91	Ligase	P46934	NEDD4	627.A.CYS	Addition by Cys	Carbonyl(cys)	4YU	1.47	1.25	2.05	3.91	3.91	9.60	1.28	0.84	1.37	1.41	1.41	4.71
3kwb	Hydrolase	P43235	CATK	1025.X.CYS	Addition by Cys	Nitrile(cys)	ORH	5.99	5.84	2.16	0.80	0.77	1.23	1.22	1.22	1.24	8.14	2.85	9
4mzo	Hydrolase	O70370	CTSS	147.A.CYS	Addition by Cys	Nitrile(cys)	2EW	4.60	1.83	5.73	1.18	0.91	2.40	1.06	1.06	1.35	1.10	1.10	2.67
4dmx	Hydrolase	P43235	CATK	25.A.CYS	Addition by Cys	Nitrile(cys)	0LB	6.02	2.35	6.02	1.35	1.04	5.80	0.46	0.46	1.45	5.85	1.61	5.85
4dmy	Hydrolase	P43235	CATK	25.A.CYS	Addition by Cys	Nitrile(cys)	0LC	1.54	1.54	2.81	6.11	3.63	10.01	0.31	0.31	1.3	4.24	1.72	4.24
2xu1	Hydrolase	P07711	CATL1	25.A.CYS	Addition by Cys	Nitrile(cys)	424	2.08	1.75	2.68	0.39	0.25	1.76	6.97	6.97	1.87	12.10	5.80	12
2yjc	Hydrolase	P07711	CATL1	25.A.CYS	Addition by Cys	Nitrile(cys)	424	2.00	1.23	2.4	0.39	0.29	11.99	0.40	0.40	1.45	7.40	5.99	7.4
5f02	Hydrolase	P07711	CATL1	25.A.CYS	Addition by Cys	Nitrile(cys)	5T9	3.99	2.57	3.99	1.03	0.97	1.00	1.09	1.09	1.53	12.16	6.21	12.5
5tdi	Hydrolase	P43235	CATK	25.A.CYS	Addition by Cys	Nitrile(cys)	7AS	9.81	2.79	9.81	1.69	1.52	8.40	1.44	1.44	2.42	1.41	1.41	8.27
5maj	Hydrolase	P07711	CATL1	25.A.CYS	Addition by Cys	Nitrile(cys)	7KH	1.52	1.52	2.69	2.67	2.65	2.84	1.24	1.24	1.24	0.48	0.48	6.42
5mae	Hydrolase	P07711	CATL1	25.A.CYS	Addition by Cys	Nitrile(cys)	7KN	2.50	1.66	2.5	8.54	3.81	10.09	6.14	6.14	6.14	0.60	0.60	0.78
3hwn	Hydrolase	P07711	CATL1	25.A.CYS	Addition by Cys	Nitrile(cys)	BD	6.97	6.97	1.39	0.69	0.69	0.78	0.42	0.42	1.45	8.24	6.78	8.24
1ms6	Hydrolase	P25774	CATS	25.A.CYS	Addition by Cys	Nitrile(cys)	VLN	2.32	2.10	2.35	0.98	0.47	3.62	1.04	1.04	3.26	5.06	1.59	5.06
2ft2	Hydrolase	P25774	CATS	25.A.CYS	Addition by Cys	Nitrile(cys)	C28	1.71	1.57	3.06	0.92	0.92	5.30	1.51	1.28	1.51	1.14	1.14	2.12
2frq	Hydrolase	P25774	CATS	25.B.CYS	Addition by Cys	Nitrile(cys)	C71	3.96	2.07	3.96	3.75	2.40	3.72	1.40	1.40	2.23	6.14	4.62	6.14
2r6n	Hydrolase	P43235	CATK	25.A.CYS	Addition by Cys	Nitrile(cys)	CKE	1.48	1.35	1.61	0.90	0.88	0.90	1.87	0.96	2.34	3.44	1.21	3.4
2fq9	Hydrolase	P43235	CATK	25.A.CYS	Addition by Cys	Nitrile(cys)	CRJ	2.74	1.28	2.74	0.78	0.78	6.17	1.30	1.30	2.13	3.92	1.56	3.92
2fud	Hydrolase	P25774	CATS	25.A.CYS	Addition by Cys	Nitrile(cys)	CRL	1.57	1.57	2.19	0.42	0.42	1.23	1.24	1.24	2.77	0.58	0.58	4.77
2fra	Hydrolase	P25774	CATS	25.A.CYS	Addition by Cys	Nitrile(cys)	CRV	1.86	1.33	2.06	0.49	0.49	1.05	1.58	1.42	2.56	3.55	2.03	3.55
2xu4	Hydrolase	P07711	CATL1	25.A.CYS	Addition by Cys	Nitrile(cys)	DJT	2.27	1.29	2.6	1.42	1.36	1.42	1.54	1.54	2.54	1.22	1.22	3.5
3n4c	Hydrolase	P25774	CATS	25.A.CYS	Addition by Cys	Nitrile(cys)	EF3	3.27	3.27	9.29	1.11	0.82	1.11	0.92	0.92	3.71	2.78	0.49	2.78
5mqy	Hydrolase	P07711	CATL1	25.A.CYS	Addition by Cys	Nitrile(cys)	GH4	2.15	1.42	2.22	5.75	1.77	5.76	5.43	0.50	5.43	6.67	0.76	6.67
1u9v	Hydrolase	P43235	CATK	25.A.CYS	Addition by Cys	Nitrile(cys)	IHE	2.27	2.22	2.6	2.67	2.65	4.00	2.66	2.60	3.94	3.26	2.92	7.5
1u9w	Hydrolase	P43235	CATK	25.A.CYS	Addition by Cys	Nitrile(cys)	IHI	3.04	1.18	3.09	1.06	0.89	7.21	7.06	7.06	2.21	7.46	1.45	7.45
1u9x	Hydrolase	P43235	CATK	25.A.CYS	Addition by Cys	Nitrile(cys)	IHJ	4.24	3.79	6.17	9.06	4.59	9.44	1.37	1.37	9.05	6.79	1.32	6.79
3kwz	Hydrolase	P43235	CATK	25.A.CYS	Addition by Cys	Nitrile(cys)	KWZ	2.56	1.31	2.55	8.56	1.83	8.51	1.61	1.61	2.63	7.13	3.02	9.94
3hha	Hydrolase	P07711	CATL1	25.A.CYS	Addition by Cys	Nitrile(cys)	NOW	1.60	1.59	1.97	0.33	0.33	0.58	0.75	0.75	1.71	0.49	0.49	4.06
3o0u	Hydrolase	P43235	CATK	25.A.CYS	Addition by Cys	Nitrile(cys)	O47	2.04	1.02	2.66	0.83	0.77	0.83	1.24	1.24	2.25	1.55	1.55	14.2
3o1g	Hydrolase	P43235	CATK	25.A.CYS	Addition by Cys	Nitrile(cys)	O75	0.97	0.97	1.4	1.19	1.19	1.32	6.60	2.26	6.57	1.44	1.44	14.13
3kw9	Hydrolase	P43235	CATK	25.A.CYS	Addition by Cys	Nitrile(cys)	ORG	1.58	1.41	1.58	0.46	0.36	0.52	1.26	0.97	2.25	7.96	1.60	9.33

4axm	Hydrolase	P07711	CATL1	25.A.CYS	Addition by Cys	Nitrile(cys)	V65	2.39	1.20	3.34	3.24	3.11	6.22	0.43	0.43	2.59	0.84	0.84	4.23
2xu3	Hydrolase	P07711	CATL1	25.A.CYS	Addition by Cys	Nitrile(cys)	XU3	1.71	1.71	2.56	9.38	8.12	10.74	1.28	1.28	1.85	5.30	1.44	5.3
2xu5	Hydrolase	P07711	CATL1	25.A.CYS	Addition by Cys	Nitrile(cys)	XU5	1.82	1.82	3.1	0.56	0.51	1.56	0.85	0.85	1.98	5.77	2.06	5.77
2r9m	Hydrolase	P25774	CATS	25.A.CYS	Addition by Cys	Nitrile(cys)	Y11	2.05	1.81	2.05	0.78	0.78	0.98	0.54	0.54	2.65	3.29	1.04	5.21
2r9n	Hydrolase	P25774	CATS	25.A.CYS	Addition by Cys	Nitrile(cys)	Y14	2.25	1.81	2.76	1.08	0.84	1.13	0.58	0.58	2.63	1.40	1.40	5.92
2r9o	Hydrolase	P25774	CATS	25.A.CYS	Addition by Cys	Nitrile(cys)	Y15	4.39	2.07	4.39	2.26	0.73	2.54	2.29	2.29	2.01	3.33	3.33	5.83
2yj2	Hydrolase	P07711	CATL1	25.A.CYS	Addition by Cys	Nitrile(cys)	YJ2	5.91	2.68	2.5	0.49	0.27	1.37	2.89	2.89	1.86	5.69	5.69	10.61
2yj8	Hydrolase	P07711	CATL1	25.A.CYS	Addition by Cys	Nitrile(cys)	YJ8	3.65	2.04	3.65	0.37	0.32	1.27	0.38	0.38	1.69	5.67	3.21	6.08
2yj9	Hydrolase	P07711	CATL1	25.A.CYS	Addition by Cys	Nitrile(cys)	YJ9	5.12	5.12	2.95	0.55	0.55	0.65	0.68	0.68	1.74	6.27	1.49	6.26
2yjb	Hydrolase	P07711	CATL1	25.A.CYS	Addition by Cys	Nitrile(cys)	YJ9	4.32	4.28	4.88	1.32	1.29	1.32	1.05	1.05	1.57	6.20	6.20	11.1
3s3j	Transferase	P21980	TGM2	277.A.CYS	Addition by Cys	Nitrile(cys)	Peptide	2.05	2.05	3.27	2.41	2.25	10.95	17.99	17.99	5.66	1.25	1.25	3.72
1gmy	Hydrolase	P07858	CATB	29.A.CYS	Addition by Cys	Nitrile(cys)	AEM	2.31	1.77	2.81	1.41	1.32	1.41	1.66	1.66	5.74	1.15	1.15	10.05
6ay2	Hydrolase	P07858	CATB	29.A.CYS	Addition by Cys	Nitrile(cys)	C1G	1.62	1.09	3.12	2.72	2.72	9.85	2.45	1.41	2.43	2.48	2.22	9.71
3i4a	Hydrolase	O94760	DDAH1	274.A.CYS	Addition by Cys	Guanyl	LN5	1.57	1.39	1.69	0.96	0.67	0.96	0.77	0.77	3.75	0.81	0.81	3.76
3p8e	Hydrolase	O94760	DDAH1	274.A.CYS	Addition by Cys	Guanyl	LN7	1.98	1.37	2.78	1.95	1.28	2.09	0.85	0.85	2.39	1.93	1.93	3.62
5e7r	Transferase	Q15750	TAB1	174.A.CYS	Substitution by Cys	Halide	5KW	4.28	1.67	4.84	1.09	0.94	1.16	2.87	2.66	4.75	1.46	1.46	7.05
5vqx	Transferase	P03366	GAG-POL	181.A.CYS	Substitution by Cys	Halide	9HY	1.71	1.71	1.85	1.88	1.58	10.58	1.06	1.06	2.56	0.41	0.41	5.14
3b0r	Transcription	P37231	PPAR-γ	285.A.CYS	Substitution by Cys	Halide	GW9	1.73	0.70	1.73	0.58	0.58	1.01	4.11	4.11	4.62	1.06	1.06	7.19
4yqm	Transferase	P78417	GSTO1	32.A.CYS	Substitution by Cys	Halide	4G9	1.67	1.67	2.84	2.31	2.31	2.50	5.21	5.21	5.21	8.56	1.20	8.56
4yqu	Transferase	P78417	GSTO1	32.A.CYS	Substitution by Cys	Halide	4GB	1.43	1.42	2.15	5.88	3.03	5.88	0.80	0.80	1.81	0.34	0.34	6.25
4yqv	Transferase	P78417	GSTO1	32.A.CYS	Substitution by Cys	Halide	4GG	1.48	1.11	2.07	1.97	1.41	1.97	4.54	4.54	4.54	0.82	0.82	8.83
2tod	Lyase	P07805	DCOR	360.A.CYS	Substitution by Cys	Halide	DMO	0.96	0.90	2.29	1.23	1.17	1.23	1.24	1.19	14.03	1.34	1.34	6.28
4mz4	Transferase	O92972	POLG	366.A.CYS	Substitution by Cys	Halide	2F3	0.87	0.87	2.02	8.34	3.43	8.59	4.47	4.47	4.64	0.58	0.58	6.33
3tyq	Transferase	O92972	POLG	366.A.CYS	Substitution by Cys	Halide	HI4	2.30	1.50	1.03	7.03	7.03	10.16	4.37	4.37	2.13	0.77	0.77	7.03
3v4o	Hydrolase	Q9UDY8	MALT1	464.A.CYS	Substitution by Cys	Halide	Peptide	2.38	2.38	3.63	7.13	2.38	7.93	0.68	0.68	2.95	1.80	1.80	1.85
1x83	Isomerase	Q46822	IDI	67.A.CYS	Substitution by Cys	Halide	SBH	3.29	1.63	3.28	2.45	2.45	5.41	4.74	4.74	3.56	1.52	1.52	3.07
5mjb	Transferase	P54762	EPHB1	703.A.CYS	Substitution by Cys	Halide	7O3	1.36	0.55	4.86	2.21	2.17	2.25	6.63	6.63	6.63	1.70	0.52	1.84
5l6o	Transferase	P54753	EPHB3	717.A.CYS	Substitution by Cys	Halide	6P6	1.92	0.73	2.27	1.03	1.03	1.20	3.96	3.96	4.06	0.88	0.88	7.37
5l6p	Transferase	P54753	EPHB3	717.A.CYS	Substitution by Cys	Halide	6P8	1.15	1.15	2.07	3.65	1.25	3.65	6.63	5.49	6.63	0.47	0.47	6.83
4xz1	Transferase	P43403	ZAP70	78.A.CYS	Substitution by Cys	Halide	4N8	2.18	1.34	2.33	4.81	3.35	5.00	5.24	5.24	5.24	4.77	3.24	6.82
4xz0	Transferase	P43403	ZAP70	117.A.CYS	Substitution by Cys	Others	4N5	3.49	0.97	3.48	7.43	6.01	8.00	7.43	7.43	7.43	2.76	2.76	6.89
1nms	Hydrolase	P42574	CASP3	163.A.CYS	Substitution by Cys	Others	161	1.49	1.49	2.76	0.58	0.54	5.60	1.44	1.44	5.18	0.40	0.40	3.52
3kjf	Hydrolase	P42574	CASP3	163.A.CYS	Substitution by Cys	Others	B92	2.39	2.39	5.58	0.90	0.67	3.68	2.50	1.16	2.87	0.75	0.75	4.72
4jje	Hydrolase	P42574	CASP3	163.A.CYS	Substitution by Cys	Others	Peptide	2.92	1.71	2.92	2.32	1.50	7.63	2.60	2.46	6.55	3.11	3.11	5.12
4jj8	Hydrolase	P55210	CASP7	186.A.CYS	Substitution by Cys	Others	Peptide	2.43	1.59	2.88	2.33	1.37	5.50	5.51	3.62	7.79	5.00	4.76	5.73
5w1y	Transferase	Q9NQR1	KMT5A	311.A.CYS	Substitution by Cys	Others	9SV	1.77	0.83	1.77	3.83	2.84	3.83	0.42	0.42	1.42	2.09	2.09	10.73
3kjn	Hydrolase	Q14790	CASP8	360.A.CYS	Substitution by Cys	Others	B93	2.10	2.10	3.14	1.61	1.43	6.61	1.26	0.31	2.36	4.42	4.42	5.87
3kjq	Hydrolase	Q14790	CASP8	360.A.CYS	Substitution by Cys	Others	B94	2.08	2.08	3.36	1.01	1.01	1.37	0.62	0.62	4.53	3.74	1.19	3.74
4jj7	Hydrolase	Q14790	CASP8	360.A.CYS	Substitution by Cys	Others	Peptide	1.87	1.36	2.7	2.06	2.06	1.74	3.85	2.92	6.38	6.20	3.48	6.2
4yhf	Transferase	Q06187	BTK	481.A.CYS	Substitution by Cys	Others	4C9	2.10	1.66	2.84	0.39	0.38	0.56	2.90	2.90	3.98	2.00	2.00	8.19
4pnc	Hydrolase	C3TPN7	MAP	59.A.CYS	Substitution by Cys	Others	7NP	1.99	1.21	2.87	3.08	3.04	3.62	1.74	0.75	1.74	0.79	0.79	4.09
4pe0	Metal binding protein	P02638	S100-B	84.A.CYS	Substitution by Cys	Others	NQS	1.72	1.72	3.65	5.54	1.71	5.54	5.43	2.79	5.43	0.81	0.81	5.19
4onm	Ligase	Q15819	UBE2V2	87.B.CYS	Substitution by Cys	Others	N2F	2.90	1.23	2.9	0.26	0.26	0.51	1.08	1.08	2.78	0.19	0.19	3.84
1khp	Hydrolase	P00784	PAPA1	25.A.CYS	Substitution by Cys	Others	Peptide	1.77	1.77	2.71	1.18	1.18	1.79	1.04	1.04	2.67	1.90	1.54	2.24

1khq	Hydrolase	P00784	PAPA1	25.A.CYS	Substitution by Cys	Others	Peptide	2.41	1.41	2.45	5.15	5.07	5.15	1.56	0.77	2.56	2.09	1.21	2.56
3of9	Hydrolase	P07711	CATL1	26.A.CYS	Substitution by Cys	Others	I0X	2.63	2.63	3.7	3.77	2.93	10.97	7.69	7.69	3.54	10.91	2.66	10.9
5v6v	Hydrolase	P01116	KRAS	12.B.CYS	Ring Opening by Cys	Heterocyclic	8YA	1.01	0.72	1.1	0.80	0.80	3.47	11.52	10.89	11.53	2.66	2.66	3.76
2a5k	Hydrolase	P0C6X7	REP	145.B.CYS	Ring Opening by Cys	Heterocyclic	AZP	3.43	1.45	3.7	1.74	1.48	6.08	1.35	1.04	2.79	5.24	2.98	6.56
2cno	Hydrolase	P42574	CASP3	163.A.CYS	Ring Opening by Cys	Heterocyclic	M60	2.93	1.76	3.39	4.75	4.30	6.88	9.52	5.66	3.85	6.09	5.58	8.92
2cdr	Hydrolase	P42574	CASP3	163.A.CYS	Ring Opening by Cys	Heterocyclic	Peptide	3.05	1.49	6.18	6.36	3.19	6.36	3.58	2.21	6.24	10.63	7.60	10.69
2cnk	Hydrolase	P42574	CASP3	163.A.CYS	Ring Opening by Cys	Heterocyclic	Peptide	3.07	2.46	3.29	6.94	4.58	6.94	2.19	1.33	3.06	10.30	7.66	13.3
2cnl	Hydrolase	P42574	CASP3	163.A.CYS	Ring Opening by Cys	Heterocyclic	Peptide	2.32	1.97	2.4	4.30	2.86	9.52	2.25	1.61	2.37	9.53	9.53	10.78
2cnn	Hydrolase	P42574	CASP3	163.A.CYS	Ring Opening by Cys	Heterocyclic	Peptide	3.13	1.98	3.13	4.79	3.34	4.87	3.54	3.51	2.33	4.88	4.88	6.85
2gkj	Isomerase	P44859	DAPF	217.A.CYS	Ring Opening by Cys	Heterocyclic	ZDR	1.60	0.97	1.78	0.34	0.34	1.39	0.88	0.88	2.07	1.19	1.19	3.92
3bcn	Hydrolase	A5YVK8	A5YVK8	25.A.CYS	Ring Opening by Cys	Heterocyclic	E64	2.20	1.03	2.2	4.00	3.89	4.01	2.66	2.39	4.08	2.75	2.75	5.04
2pre	Hydrolase	A8DS38	A8DS38	25.A.CYS	Ring Opening by Cys	Heterocyclic	E64	1.58	1.58	3.15	2.87	2.70	5.14	1.43	1.43	2.91	5.37	5.37	10.95
1atk	Hydrolase	P43235	CATK	25.A.CYS	Ring Opening by Cys	Heterocyclic	E64	1.49	1.49	3.7	1.33	1.08	2.36	0.70	0.65	5.41	5.82	5.82	10.87
3ekm	Isomerase	Q9LFG2	DAPF	254.A.CYS	Ring Opening by Cys	Heterocyclic	ZDR	1.71	1.37	1.71	1.15	1.04	1.23	0.83	0.83	2.16	1.41	1.41	4.07
1csb	Hydrolase	P07858	CATB	29.A.CYS	Ring Opening by Cys	Heterocyclic	EP0	1.37	1.37	2.97	1.04	0.96	1.59	1.31	0.81	2.9	2.12	1.33	2.28
3bpf	Hydrolase	Q9N6S8	Q9N6S8	42.A.CYS	Ring Opening by Cys	Heterocyclic	E64	1.93	1.43	2.43	6.02	6.02	12.82	3.04	1.08	3.59	6.72	3.31	6.72
1nfz	Isomerase	Q46822	IDI	67.A.CYS	Ring Opening by Cys	Heterocyclic	EIP	1.38	1.38	2.13	1.64	0.52	1.72	1.87	1.87	2.19	2.98	2.98	4.22
2gke	Isomerase	P44859	DAPF	73.A.CYS	Ring Opening by Cys	Heterocyclic	ZDP	1.30	1.30	1.97	0.47	0.47	1.66	1.11	1.10	1.56	4.79	4.79	8.75
3ejx	Isomerase	Q9LFG2	DAPF	99.A.CYS	Ring Opening by Cys	Heterocyclic	ZDP	1.32	1.00	1.58	1.25	1.25	2.09	0.92	0.92	2.48	4.72	4.72	6.42
4lv6	Hydrolase	P01116	KRAS	12.A.CYS	Disulfide Formation	Sulfydryl	20H	2.97	1.22	2.97	1.07	0.79	3.76	0.59	0.59	1.59	2.51	0.43	3.54
4rqx	Oxidoreductase	Q13162	PRDX4	124.A.CYS	Disulfide Formation	Sulfydryl	COM	1.85	0.94	4.89	1.85	1.85	4.28	2.57	2.57	3.41	2.80	2.80	4.45
2mlm	Hydrolase	R9YTM5	R9YTM5	126.A.CYS	Disulfide Formation	Sulfydryl	2W7	2.68	2.68	3.57	5.06	5.06	14.75	1.51	1.51	2.51	1.62	1.62	9.2
3orx	Transferase	O15530	PDPK1	148.G.CYS	Disulfide Formation	Sulfydryl	1F8	1.00	0.90	3.13	7.55	7.19	7.55	0.21	0.21	1.21	5.98	5.98	11.14
3orz	Transferase	O15530	PDPK1	148.A.CYS	Disulfide Formation	Sulfydryl	2A2	1.41	1.41	2.4	0.72	0.72	1.73	1.64	1.64	2.65	1.93	1.93	3.2
3otu	Transferase	O15530	PDPK1	148.A.CYS	Disulfide Formation	Sulfydryl	J30	1.60	1.59	2.39	2.30	1.59	3.15	3.20	3.20	2.94	3.04	1.96	3.31
2ipp	Hydrolase	P07858	CATB	29.A.CYS	Disulfide Formation	Sulfydryl	PYS	2.65	2.65	3.13	3.59	2.77	6.36	3.69	3.69	3.63	3.67	3.01	6.79
2ijn	Transferase	Q99AU2	Q99AU2	366.A.CYS	Disulfide Formation	Sulfydryl	221	2.44	1.99	2.44	7.70	1.89	8.52	2.47	2.47	3.47	5.99	2.16	5.99
4pok	Oxidoreductase	P10599	TXN	69.A.CYS	Disulfide Formation	Sulfydryl	COM	2.03	1.08	2.02	3.97	3.72	3.97	2.80	2.80	3.01	3.96	1.84	3.96
4pe1	Metal binding protein	P02638	S100-B	84.A.CYS	Disulfide Formation	Sulfydryl	DCD	1.12	1.04	1.36	2.18	2.16	2.81	3.00	3.00	3.12	2.10	1.67	5.13
1zlr	Hydrolase	P03951	FA11	195.A.SER	Addition to Ser	Boric acid	368	1.74	1.50	1.74	1.91	1.80	1.91	2.61	1.26	2.26	1.84	1.84	9.92
1z6f	Hydrolase	P0AEB2	DACA	44.A.SER	Addition by Ser	Boric acid	BO9	4.46	4.18	4.55	4.31	2.24	10.41	2.94	2.94	8.83	5.37	3.24	5.36
2y59	Hydrolase	P39045	DAC	49.A.SER	Addition by Ser	Boric acid	BH	1.78	1.77	3.25	3.54	3.29	3.54	2.74	2.74	3.73	2.84	2.64	3.51
2xln	Hydrolase	P39045	DAC	49.A.SER	Addition by Ser	Boric acid	EWA	2.07	2.07	2.48	1.25	1.25	1.36	3.01	2.40	3.34	2.44	2.44	3.53
2xk1	Hydrolase	P39045	DAC	49.A.SER	Addition by Ser	Boric acid	EWB	1.94	0.85	1.94	2.57	1.88	5.41	5.00	5.00	4.99	2.97	2.97	4.63
4b4x	Hydrolase	P39045	DAC	49.A.SER	Addition by Ser	Boric acid	HQZ	1.57	1.36	2.07	3.09	0.97	3.09	1.07	1.07	1.48	1.46	1.46	3.14
2y4a	Hydrolase	P39045	DAC	49.A.SER	Addition by Ser	Boric acid	ZA	4.37	1.01	4.76	1.94	1.89	1.94	3.97	3.97	3.97	1.35	1.35	4.41

4x6t	Hydrolase	P9WKD3	BLAC	84.A.SER	Addition by Ser	Boric acid	3Y6	2.03	1.37	1.82	7.36	7.23	7.50	1.41	1.41	2.68	5.04	5.04	6.99
2i03	Hydrolase	P27487	DPP4	630.B.SER	Addition by Ser	Nitrile(ser)	AXD	2.39	1.64	2.96	1.06	1.06	4.32	1.46	0.52	1.51	38.93	38.93	5.12
3bjm	Hydrolase	P27487	DPP4	630.A.SER	Addition by Ser	Nitrile(ser)	BJM	1.24	1.02	1.45	2.54	1.77	3.03	0.34	0.34	1.38	0.50	0.50	2.61
3w2t	Hydrolase	P27487	DPP4	630.A.SER	Addition by Ser	Nitrile(ser)	LF7	1.38	1.34	1.52	2.63	2.09	2.79	0.73	0.73	2.15	2.66	2.66	7.36
5kre	Hydrolase	Q5VWZ2	LYPLAL1	124.A.SER	Addition by Ser	Carbonyl(ser)	6WG	1.20	1.20	1.33	1.05	1.02	1.07	1.48	1.48	2.89	1.36	1.36	4.62
4uuq	Hydrolase	Q99685	MGLL	132.A.SER	Addition by Ser	Carbonyl(ser)	64D	0.96	0.96	1.54	9.21	1.30	9.21	1.40	1.40	2.39	0.47	0.47	6.52
3u1i	Hydrolase	Q5UB51	POLG	135.B.SER	Addition by Ser	Carbonyl(ser)	Peptide	4.28	2.30	4.73	8.04	3.18	10.96	7.49	5.57	9.72	4.71	4.71	6.91
2a4q	Viralprotein	Q91RS4	Q91RS4	139.A.SER	Addition by Ser	Carbonyl(ser)	FNH	4.89	4.17	5.81	0.90	0.90	7.88	3.10	1.91	5.84	5.27	5.27	10.09
2oc0	Viralprotein	P27958	POLG	139.A.SER	Addition by Ser	Carbonyl(ser)	HU1	1.77	1.77	2.71	0.68	0.67	1.00	5.47	1.05	7.59	4.59	4.59	5.04
2oc1	Viralprotein	P27958	POLG	139.A.SER	Addition by Ser	Carbonyl(ser)	HU2	2.93	1.21	3.37	0.73	0.73	6.40	1.29	1.29	12.78	11.53	7.20	11.53
1qhr	Hydrolase	P00734	THRB	195.B.SER	Addition by Ser	Carbonyl(ser)	157	1.49	1.15	2.11	2.43	2.14	2.43	1.37	1.37	1.89	1.36	1.36	7.14
1qj1	Hydrolase	P00734	THRB	195.B.SER	Addition by Ser	Carbonyl(ser)	166	1.01	1.01	1.65	1.64	1.58	1.94	1.59	1.42	1.73	1.40	1.40	7.53
1qj6	Hydrolase	P00734	THRB	195.B.SER	Addition by Ser	Carbonyl(ser)	167	1.44	1.08	1.95	2.69	2.69	3.77	3.95	1.25	3.27	2.03	0.79	7.69
1zsl	Hydrolase	P03951	FA11	195.A.SER	Addition by Ser	Carbonyl(ser)	624	2.00	1.69	2.23	5.37	0.80	6.14	7.12	0.57	2.89	1.02	1.02	5.63
1zpz	Hydrolase	P03951	FA11	195.A.SER	Addition by Ser	Carbonyl(ser)	BUK	5.37	2.75	6.89	5.27	4.03	5.63	7.49	2.22	5.36	4.85	2.01	7.69
3fvf	Hydrolase	Q16651	PRSS8	195.B.SER	Addition by Ser	Carbonyl(ser)	1JZ	1.03	0.92	1.33	3.54	3.54	3.67	1.23	0.81	2.63	0.71	0.71	6.13
4ym9	Hydrolase	P00772	CELA1	195.A.SER	Addition by Ser	Carbonyl(ser)	4E4	1.61	1.24	1.67	1.97	1.50	3.32	4.80	1.29	4.89	0.70	0.70	5.04
3e0n	Hydrolase	Q16651	PRSS8	195.B.SER	Addition by Ser	Carbonyl(ser)	Peptide	2.00	1.76	8.13	8.90	6.44	11.71	7.90	7.45	11.95	2.57	0.60	6.08
1iau	Hydrolase	P10144	GZMB	195.A.SER	Addition by Ser	Carbonyl(ser)	Peptide	1.84	1.63	2.03	10.21	6.43	10.20	9.05	8.57	9.83	1.13	1.13	7.88
3e0p	Hydrolase	Q16651	PRSS8	195.B.SER	Addition by Ser	Carbonyl(ser)	B3C	3.79	3.79	4.51	8.56	2.07	8.56	3.76	3.76	10.56	7.94	4.88	7.94
3hgn	Hydrolase	P00772	CELA1	195.A.SER	Addition by Ser	Carbonyl(ser)	FRW	2.35	1.36	2.38	5.93	5.93	10.30	1.15	1.15	16.81	1.34	1.34	3.68
8est	Hydrolase	P00772	CELA1	195.E.SER	Addition by Ser	Carbonyl(ser)	GIS	0.97	0.97	1.94	0.67	0.66	0.67	2.90	1.56	3.01	2.65	2.25	2.65
1qj7	Hydrolase	P00734	THRB	195.B.SER	Addition by Ser	Carbonyl(ser)	GR1	2.48	1.75	2.48	6.56	1.16	6.77	9.21	1.16	9.06	7.70	1.88	2.17
1haz	Hydrolase	P00772	CELA1	195.B.SER	Addition by Ser	Carbonyl(ser)	IL0	3.49	3.49	7.73	4.93	4.70	4.93	7.52	1.79	7.79	1.10	1.10	6.18
2v35	Hydrolase	P00772	CELA1	195.A.SER	Addition by Ser	Carbonyl(ser)	J54	1.53	1.40	2.58	1.54	1.54	1.68	4.86	1.64	4.93	9.20	1.99	9.2
1oce	Hydrolase	P04058	ACES	200.A.SER	Addition by Ser	Carbonyl(ser)	MF2	1.46	1.26	1.85	4.16	2.87	8.17	2.90	2.90	6.5	3.24	3.24	8.69
2wke	Hydrolase	P39045	DAC	49.A.SER	Addition by Ser	Carbonyl(ser)	BIY	1.37	0.88	1.5	2.58	2.57	2.90	3.90	2.49	4.71	2.24	2.24	3.79
3zcz	Hydrolase	P39045	DAC	49.A.SER	Addition by Ser	Carbonyl(ser)	TFR	0.91	0.76	1.5	0.91	0.91	1.41	0.94	0.52	8.15	0.96	0.96	6.36
5ced	Hydrolase	Q6MHT0	BD3459	70.A.SER	Addition by Ser	Carbonyl(ser)	PNM	0.75	0.75	2.19	3.65	3.20	6.02	6.87	6.87	6.87	2.89	2.03	2.89
3bg8	Hydrolase	P03951	FA11	557.A.SER	Addition by Ser	Carbonyl(ser)	INH	1.07	1.07	2.31	7.55	2.86	8.97	0.92	0.92	1.96	3.86	3.86	7.96
3k84	Hydrolase	P97612	FAAH	241.A.SER	Addition by Ser	Carbonyl(ser)	K84	2.02	1.37	2.69	5.29	0.87	5.29	1.15	1.15	2.15	1.01	1.01	11.23
2wap	Hydrolase	P97612	FAAH	241.A.SER	Addition by Ser	Carbonyl(ser)	PIX	1.04	0.80	1.54	1.03	1.03	1.31	1.40	1.40	2.03	1.24	1.24	6.38
3LJ6	Hydrolase	P97612	FAAH	241.B.SER	Addition by Ser	Carbonyl(ser)	PIX	2.41	1.39	2.46	0.82	0.36	0.82	1.37	1.37	2.73	0.19	0.19	5.96
1h8i	Hydrolase	P00734	THRB	195.H.SER	Substitution by Ser	Phosphonyl	PHV	5.53	2.31	5.67	2.33	2.08	6.35	5.81	1.05	6.06	23.33	9.94	3.15
1mt5	Hydrolase	P97612	FAAH	241.A.SER	Substitution by Ser	Phosphonyl	MAY	2.33	1.54	2.33	1.35	1.02	1.35	6.22	6.22	6.62	1.68	1.68	2.04
1scw	Hydrolase	P15555	DAC	62.A.SER	Substitution by Ser	Phosphonyl	CP5	1.39	1.39	2.86	4.98	3.54	1.58	3.25	3.25	3.14	1.53	1.53	6.19
1rtl	Viralprotein	O39914	NS4a/b	139.A.SER	Ring Opening by Ser	Lactam	CPX	8.42	8.42	2.01	8.87	4.58	8.87	7.34	1.32	2.87	2.84	2.84	10.08
3upn	Hydrolase	P9WKD1	PBPA	222.A.SER	Ring Opening by Ser	Lactam	IM2	2.29	2.27	2.95	3.05	2.87	4.83	2.65	2.05	2.6	3.96	3.96	8.04
3upp	Hydrolase	P9WKD1	PBPA	222.A.SER	Ring Opening by Ser	Lactam	PCZ	1.86	1.06	1.86	1.52	1.30	3.38	1.41	1.11	3.01	2.79	2.79	6.23
3upo	Hydrolase	P9WKD1	PBPA	222.A.SER	Ring Opening by Ser	Lactam	PNM	2.62	2.10	2.85	2.26	0.66	7.26	2.52	1.55	2.57	2.18	2.02	2.18
3zfz	Hydrolase	A0A0H3JPA5	MECA	403.A.SER	Ring Opening by Ser	Lactam	AI8	1.92	1.92	2.91	1.60	1.60	3.50	1.68	1.68	3.11	3.08	1.56	3.09
4dki	Hydrolase	A0A0H2WXF8	MECA	403.A.SER	Ring Opening by Ser	Lactam	RB6	4.05	1.91	5.89	3.49	2.85	3.49	3.10	2.70	3.13	0.99	0.99	3.36

3mze	Hydrolase	P0AEB2	DACA	44.A.SER	Ring Opening by Ser	Lactam	CFX	1.07	1.07	2.41	2.43	1.33	2.48	2.41	2.41	2.57	1.35	1.35	1.41
3mzd	Hydrolase	P0AEB2	DACA	44.A.SER	Ring Opening by Ser	Lactam	CXV	1.61	1.43	2.91	3.65	3.63	7.56	1.73	1.21	2.68	2.67	2.51	2.61
3beb	Hydrolase	P0AEB2	DACA	44.A.SER	Ring Opening by Ser	Lactam	HJ3	0.85	0.85	1.55	1.61	1.60	2.63	1.31	1.31	3.87	2.11	1.28	3.48
4ben	Hydrolase	P39045	DAC	49.B.SER	Ring Opening by Ser	Lactam	IM2	1.49	0.98	1.67	4.12	3.90	4.30	6.48	3.30	3.56	2.94	2.94	2.67
1w8y	Hydrolase	P39045	DAC	49.A.SER	Ring Opening by Ser	Lactam	NCF	1.06	0.54	2.42	7.39	1.65	7.40	4.63	2.92	2.32	0.94	0.94	5.35
2vgj	Hydrolase	P39045	DAC	49.A.SER	Ring Opening by Ser	Lactam	REC	1.82	1.81	2.63	1.79	0.69	3.20	0.96	0.82	1.85	1.06	1.06	3.6
2j8y	Hydrolase	Q8DH45	TLL2115	61.A.SER	Ring Opening by Ser	Lactam	PNM	1.68	0.95	2.6	1.24	1.23	1.63	1.49	1.10	2.67	0.83	0.83	5.25
2jbf	Hydrolase	Q8DH45	TLL2116	61.A.SER	Ring Opening by Ser	Lactam	PNM	1.46	0.98	2.06	0.68	0.38	0.68	1.03	1.03	2.25	2.37	1.06	5.51
2ex9	Hydrolase	P24228	DACB	62.A.SER	Ring Opening by Ser	Lactam	35P	1.65	1.65	2.96	1.23	1.23	1.90	1.17	1.17	2.68	1.58	1.58	6.48
2ex6	Hydrolase	P24228	DACB	62.A.SER	Ring Opening by Ser	Lactam	AIX	0.98	0.69	3.45	3.34	3.26	3.34	3.70	1.93	3.67	3.99	0.90	3.99
2ex8	Hydrolase	P24228	DACB	62.A.SER	Ring Opening by Ser	Lactam	PNM	2.14	1.39	2.73	3.23	2.34	3.23	1.47	1.47	2.25	0.60	0.60	5.29
1yqs	Hydrolase	P15555	DAC	62.A.SER	Ring Opening by Ser	Lactam	BSA	1.67	1.47	3.35	6.44	6.04	6.64	6.23	6.23	6.84	1.07	1.07	5.25
1cef	Hydrolase	P15555	DAC	62.A.SER	Ring Opening by Ser	Lactam	CEF	2.67	1.79	3.08	3.13	1.42	3.13	2.74	2.34	5.49	2.80	1.67	2.97
1hvb	Hydrolase	P15555	DAC	62.A.SER	Ring Opening by Ser	Lactam	CEH	2.35	1.44	2.35	6.65	5.42	6.57	4.40	2.54	8.62	2.60	2.60	6
1ceg	Hydrolase	P15555	DAC	62.A.SER	Ring Opening by Ser	Lactam	CEP	1.26	1.06	2.67	1.70	1.68	1.74	1.72	1.72	8.6	1.19	1.19	4.84
2exb	Hydrolase	P24228	DACB	62.A.SER	Ring Opening by Ser	Lactam	FXM	1.67	1.62	4.77	2.98	2.47	3.17	2.67	2.64	3.37	5.72	5.72	6.61
1pw8	Hydrolase	P15555	DAC	62.A.SER	Ring Opening by Ser	Lactam	H2A	2.37	1.94	2.73	2.65	1.17	3.27	1.59	1.09	5.68	3.38	1.41	3.38
1pwg	Hydrolase	P15555	DAC	62.A.SER	Ring Opening by Ser	Lactam	HE0	2.28	2.21	2.52	0.92	0.92	1.77	1.13	0.98	3.4	1.03	1.03	4.82
5eph	Hydrolase	Q3SAW3	BLA	62.A.SER	Ring Opening by Ser	Lactam	ID1	2.00	1.50	2	1.06	0.95	1.34	5.23	2.52	5.87	2.16	1.73	2.16
1pwc	Hydrolase	P15555	DAC	62.A.SER	Ring Opening by Ser	Lactam	PNM	2.37	1.82	2.64	1.80	1.47	1.80	1.60	1.51	3.64	1.23	1.23	7.07
3a3i	Hydrolase	A8E0K8	DACB	69.A.SER	Ring Opening by Ser	Lactam	AIX	1.51	1.32	1.9	3.03	0.86	3.10	1.18	1.18	3.56	2.42	0.62	5.2
3a3e	Hydrolase	A8E0K8	DACB	69.A.SER	Ring Opening by Ser	Lactam	CMX	2.39	1.64	4.87	2.93	2.29	2.93	2.36	2.26	3.05	2.45	1.35	2.45
3a3f	Hydrolase	A8E0K8	DACB	69.A.SER	Ring Opening by Ser	Lactam	FMZ	2.48	1.47	2.48	2.55	1.38	2.63	1.74	1.38	1.73	5.21	0.60	5.21
3bfc	Hydrolase	Q93PQ0	Q93PQ0	70.A.SER	Ring Opening by Ser	Lactam	IM2	1.03	1.03	3.46	4.67	4.48	6.26	6.30	1.09	6.31	2.08	1.28	2.19
3d4f	Hydrolase	P0AD64	BLA	70.A.SER	Ring Opening by Ser	Lactam	LN1	2.20	1.44	2.35	4.02	3.84	5.86	3.09	2.84	3.42	6.86	6.57	6.86
3bfg	Hydrolase	Q93PQ0	Q93PQ0	70.A.SER	Ring Opening by Ser	Lactam	MER	3.58	1.83	3.58	2.22	1.97	4.30	1.85	1.85	2.85	9.03	2.41	9.03
2h5s	Hydrolase	P0AD64	BLA	70.A.SER	Ring Opening by Ser	Lactam	SA2	1.70	1.59	2.82	5.20	5.18	5.83	2.40	2.40	6.46	3.62	2.52	3.62
3bff	Hydrolase	Q93PQ0	Q93PQ0	70.A.SER	Ring Opening by Ser	Lactam	SFR	1.73	1.02	2.22	2.84	1.24	2.84	1.65	1.12	2.55	3.28	1.56	5.52
1vm1	Hydrolase	P0AD64	BLA	70.A.SER	Ring Opening by Ser	Lactam	TBE	1.73	1.31	2.26	5.37	3.05	10.01	2.90	2.90	6.08	3.01	3.01	5.37
3pae	Hydrolase	Q8RLA6	BLAOXA-33	81.A.SER	Ring Opening by Ser	Lactam	4J6	1.52	1.52	2.76	1.09	1.01	1.05	2.58	2.51	3.51	1.03	1.03	3.73

3fzc	Hydrolase	Q8RLA6	BLAOXA-33	81.A.SER	Ring Opening by Ser	Lactam	MXF	1.64	1.64	2.04	3.43	3.41	3.48	3.19	2.30	2.77	4.14	2.85	3.16
3fv7	Hydrolase	Q8RLA6	BLAOXA-33	81.A.SER	Ring Opening by Ser	Lactam	MXS	2.86	1.56	2.85	7.55	7.55	7.56	3.29	2.61	3.64	2.76	2.50	7.86
3cg5	Hydrolase	P9WKD3	BLAC	84.A.SER	Ring Opening by Ser	Lactam	ISS	1.60	1.22	1.95	3.77	1.39	3.80	0.84	0.84	1.85	3.16	1.42	3.16
4q8i	Hydrolase	P9WKD3	BLAC	84.A.SER	Ring Opening by Ser	Lactam	TEB	1.74	1.74	3.04	1.30	1.27	2.85	1.87	1.68	2.56	1.71	1.54	3.43
1awf	Hydrolase	P00734	THRB	195.H.SER	Ring Opening by Ser	Lactone	GR4	1.43	1.43	2.67	1.74	1.74	1.96	1.64	1.64	5.2	3.50	3.50	5.75
2px6	Transferase	P49327	FASN	2308.A.SER	Ring Opening by Ser	Lactone	DH9	2.74	1.63	2.74	3.38	1.46	10.17	3.41	1.57	8.35	7.83	6.46	7.83

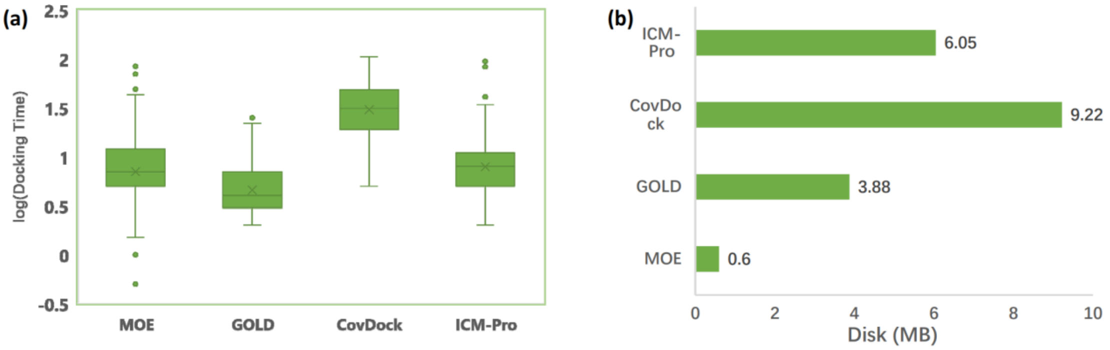

Figure S1. Some computional costs in this work. (**a**) Docking time (minute) for four covalent docking tools. (**b**)The average disk of docking files.

Protein Varieties	uniportID	n	MOE	GOLD	CovDock	ICM-Pro
Hydrolase	P43235	24	2.95	1.60	1.71	5.03
	P07711	18	2.33	1.18	1.07	5.99
	P25774	14	2.28	1.03	1.44	3.04
	P01116	14	2.13	1.77	1.57	2.43
	P42574	12	2.74	4.47	2.51	4.06
	P39045	10	1.68	2.57	3.46	1.85
	P15555	8	2.31	2.89	2.23	1.38
	P00734	6	1.46	2.38	2.79	2.76
	P00772	5	1.61	1.97	4.80	1.34
	Q14790	5	2.08	2.04	1.26	6.20
	P0AEB2	4	1.34	3.04	2.07	2.39
	P24228	4	1.66	3.11	2.07	2.79
	P03951	4	1.87	5.32	4.87	2.85
	P04936	4	2.08	1.26	0.91	2.74
	P97612	4	2.18	1.19	1.39	1.12
	P07858	4	1.96	2.07	2.05	2.30
	A8E0K8	3	2.39	2.93	1.74	2.45
	O94760	3	1.57	1.27	0.85	0.82
	P27487	3	1.38	2.54	0.73	2.66
	Q16651	3	2.00	8.56	3.76	2.57
	P0AD64	3	1.73	5.20	2.90	3.62
	Q93PQ0	3	1.73	2.84	1.85	3.28
	P9WKD3	3	1.74	3.77	1.41	3.16
	Q8RLA6	3	1.64	3.43	3.19	2.76
	P0C6X7	3	2.95	1.74	2.51	5.24
	P15273	3	1.79	1.19	0.78	1.36
	P9WKD1	3	2.29	2.26	2.52	2.79
	P00784	2	2.09	3.16	1.30	2.00
	Q8DH45	2	1.57	0.96	1.26	1.60
	P55210	2	2.60	3.52	3.31	3.18
	Q9NAW4	2	3.76	1.93	3.04	2.64
	P01112	2	1.78	3.46	3.60	4.19
	O60911	1	1.99	1.51	8.81	1.09
	P10144	1	1.84	10.21	9.05	1.13
	A5YVK8	1	2.20	4.00	2.66	2.75
	Q9N6S8	1	1.93	6.02	3.04	6.72
	A8DS38	1	1.58	2.87	1.43	5.37
	R9YTM5	1	2.68	5.06	1.51	1.62
	A0A0H2WXF8	1	4.05	3.49	3.10	0.99
	A0A0H3JPA5	1	1.92	1.60	1.68	3.08
	B9A5C1	1	3.54	1.09	1.81	2.93
	C3TPN7	1	1.99	3.08	1.74	0.79
	O91734	1	2.70	4.35	8.03	6.04
	P04058	1	1.46	4.16	2.90	3.24
	P25779	1	1.56	8.08	4.77	2.06
	Q9UDY8	1	2.38	7.13	0.68	1.80
	Q6MHT0	1	0.75	3.65	6.87	2.89
	Q99685	1	0.96	9.21	1.40	0.47
	Q5UB51	1	4.28	8.04	7.49	4.71
	Q5VWZ2	1	1.20	1.05	1.48	1.36
	Q8MNY2	1	1.60	3.52	0.81	0.53
	Q3SAW3	1	2.00	1.06	5.23	2.16
	O70370	1	4.60	1.18	1.06	1.10
Transferase	P00533	16	1.97	1.19	0.77	0.91
	P52333	6	1.69	1.22	1.04	0.61
	P00523	6	2.07	3.38	1.06	2.63
	P28482	4	2.12	1.57	0.64	1.24
	Q15750	5	2.84	4.80	2.22	1.46
	P26663	3	1.49	5.18	8.15	2.01
	O15530	3	1.41	2.30	1.64	3.04
	P03366	3	1.51	1.79	0.27	0.62
	P21980	3	2.05	5.04	1.80	2.24
	P22455	3	2.18	2.24	2.18	0.59
	P78417	3	1.48	2.31	4.54	0.82
	Q08881	3	1.10	1.00	0.85	0.58
	P04818	2	1.20	3.61	1.07	1.14
	P54753	2	1.54	2.34	5.30	0.67
	P43403	2	2.84	6.12	6.34	3.76
	Q06187	2	1.87	0.66	2.43	3.10
	Q9NQR1	2	1.52	2.43	8.25	2.02
	O92972	2	1.59	7.69	4.42	0.67
	P51812	1	0.80	8.17	1.25	1.03
	P49327	1	2.74	3.38	3.41	7.83
	P00488	1	4.86	3.22	7.78	4.34
	P11362	1	1.57	7.25	1.27	0.43
	P53779	1	2.02	3.49	1.72	2.35
	Q15303	1	2.01	0.67	0.42	0.39
	Q9M6U3	1	1.51	2.12	2.60	2.39
	Q16539	1	2.17	1.29	1.28	0.37
	Q02750	1	1.08	6.25	0.40	1.95
	P54762	1	1.36	2.21	6.63	1.70
	P42336	1	2.78	2.19	1.17	0.94
	I6Y9J2	1	3.02	1.25	1.62	0.79
	O75909	1	1.86	6.54	1.53	4.10
Ligase	Q15819	1	2.90	0.26	1.08	0.19
	P46934	1	1.47	3.91	1.28	1.41
	Q4DA54	1	3.48	2.63	0.78	1.47
Lyase	P07805	1	0.96	1.23	1.24	1.34
Oxidoreductase	Q4D3W2	2	1.89	0.84	4.11	0.73
	P10599	1	2.03	3.97	2.80	3.96
	Q13162	1	1.85	1.84	2.57	2.80
	P30838	1	1.45	1.67	1.28	1.27
	P49419	1	1.36	1.05	2.17	0.90
Isomerase	P44859	2	1.45	0.40	0.99	2.99
	Q46822	2	2.33	2.05	3.31	2.25
	Q9LFG2	2	1.52	1.20	0.88	3.07
Transcription	P37231	10	2.30	7.49	3.34	6.11
	P30822	4	4.04	6.00	2.00	2.76
	Q14145	3	2.04	2.47	1.20	3.53
	Q99AU2	1	2.44	7.70	2.47	5.99
Viral Protein	P27958	2	2.35	0.70	3.38	8.06
	Q91RS4	1	4.89	0.90	3.10	5.27
	O39914	1	8.42	8.87	7.34	2.84
Metal binding protein	P02638	5	1.72	5.54	5.43	2.37

Figure S2. Median RMSD value of *Best Scored Pose* on each target. n stands for the total number of complexes for a given uniprotID.

Table S2. The number of best RMSD value cross all the docking tools for a given tool based on the receptor type for *Best Scored Pose*.

Receptor Type	MOE	GOLD	CovDock	ICM-Pro
Hydrolase	18	10	15	10
Transferase	8	2	9	12
Ligase	0	1	2	0
Lyase	1	0	0	0
Oxidoreductase	1	1	0	3
Isomerase	0	2	1	0
Transcription	1	0	3	0
Viral Protein	0	2	0	1
Metal binding protein	1	0	0	0
Total	30	18	30	26

Table S3. The generalities calculated by the generality($G(i,x)$) formula for four docking tool with *Best Scored Pose* measurement based on the receptor type.

Receptor Type	MOE	GOLD	CovDock	ICM-Pro
Hydrolase	8.8	4.9	7.4	4.9
Transferase	9.6	2.4	10.8	14.5
Ligase	0	33.3	66.7	0
Lyase	100	0	0	0
Oxidoreductase	16.7	16.7	0	50
Isomerase	0	33.3	16.7	0
Transcription	5.6	0	16.7	0
Viral Protein	0	50	0	25
Metal binding protein	20	0	0	0
$G(i,x)$	160.7	140.6	118.3	94.4

Protein Varieties	UniportID	n	MOE	GOLD	CovDock	ICM-Pro
Hydrolase	P43235	24	1.82	1.51	1.40	1.67
	P07711	18	1.79	1.29	0.95	1.89
	P25774	14	1.84	0.80	1.26	1.57
	P01116	14	1.16	1.03	1.32	1.41
	P42574	12	1.88	3.03	1.81	2.83
	P39045	10	0.99	1.76	2.61	1.85
	P15555	8	1.63	1.57	2.03	1.32
	P00734	6	1.29	1.91	1.31	1.64
	P00772	5	1.36	1.54	1.56	1.34
	O94760	5	1.37	1.27	0.85	0.81
	P0AEB2	4	1.25	1.92	1.86	1.93
	P24228	4	1.51	2.41	1.70	1.24
	P03951	4	1.60	2.33	1.09	1.92
	P04936	4	1.61	1.23	0.84	1.44
	P97612	4	1.38	0.94	1.39	1.12
	P07858	4	1.57	2.02	1.53	1.77
	A8E0K8	3	1.47	1.38	1.38	0.62
	Q14790	3	1.64	1.97	0.93	3.48
	P27487	3	1.34	1.77	0.52	2.66
	Q16651	3	1.76	3.54	3.76	0.71
	P0AD64	3	1.44	3.84	2.84	3.01
	Q93PQ0	3	1.03	1.97	1.12	1.56
	P9WKD3	3	1.37	1.39	1.41	1.54
	Q8RLA6	3	1.56	3.41	2.51	2.50
	P0C6X7	3	1.42	1.03	2.42	2.98
	P15273	3	1.40	1.15	0.78	1.36
	P9WKD1	3	2.10	1.30	1.55	2.79
	P00784	2	1.59	3.13	0.91	1.38
	Q8DH45	2	0.96	0.80	1.06	0.95
	P55210	2	1.54	2.09	2.30	3.06
	Q9NAW4	2	1.70	1.92	1.50	1.81
	P01112	2	1.61	3.36	1.44	1.68
	O60911	1	1.74	1.31	2.01	1.09
	P10144	1	1.63	6.43	8.57	1.13
	A5YVK8	1	1.03	3.89	2.39	2.75
	Q9N6S8	1	1.43	6.02	1.08	3.31
	A8DS38	1	1.58	2.70	1.43	5.37
	R9YTM5	1	2.68	5.06	1.51	1.62
	A0A0H2WXF8	1	1.91	2.85	2.70	0.99
	A0A0H3JPA5	1	1.92	1.60	1.68	1.56
	B9A5C1	1	2.25	1.09	0.83	1.62
	C3TPN7	1	1.21	3.04	0.75	0.79
	O91734	1	2.70	4.35	6.33	4.24
	P04058	1	1.26	2.87	2.90	3.24
	P25779	1	1.56	3.43	3.57	2.06
	Q9UDY8	1	2.38	2.38	0.68	1.80
	Q6MHT0	1	0.75	3.20	6.87	2.03
	Q99685	1	0.96	1.30	1.40	0.47
	Q5UB51	1	2.30	3.18	5.57	4.71
	Q5VWZ2	1	1.20	1.02	1.48	1.36
	Q8MNY2	1	1.52	3.52	0.81	0.53
	Q3SAW3	1	1.50	0.95	2.52	1.73
	O70370	1	1.83	0.91	1.06	1.10
Transferase	P00533	16	1.31	0.76	0.59	0.91
	P52333	6	1.13	0.94	0.77	0.61
	P00523	6	1.25	1.36	0.66	1.83
	Q15750	5	1.17	1.65	1.42	1.46
	P28482	4	1.46	1.00	0.64	1.07
	P26663	3	1.20	4.12	8.01	1.80
	O15530	3	1.41	1.59	1.64	1.96
	P03366	3	1.51	1.22	0.27	0.62
	P21980	3	2.05	2.25	1.80	1.30
	P22455	3	1.47	1.79	2.12	0.59
	P78417	3	1.42	2.31	4.54	0.82
	Q08881	3	1.10	0.55	0.85	0.58
	P04818	2	1.06	3.18	1.07	1.14
	P54753	2	0.94	1.14	4.72	0.67
	P43403	2	1.16	4.68	6.34	3.00
	Q06187	2	1.65	0.65	2.43	2.11
	Q9NQR1	2	0.95	1.94	8.25	2.02
	O92972	2	1.18	5.23	4.42	0.67
	P51812	1	0.80	7.24	1.23	1.03
	P49327	1	1.63	1.46	1.57	6.46
	P00488	1	2.97	3.22	7.15	3.03
	P11362	1	1.42	5.29	1.05	0.43
	P53779	1	1.76	1.90	1.57	2.35
	Q15303	1	1.90	0.67	0.35	0.39
	Q9M6U3	1	1.51	1.77	2.02	2.39
	Q16539	1	1.73	1.16	1.06	0.37
	Q02750	1	1.08	2.87	0.40	1.41
	P54762	1	0.55	2.17	6.63	0.52
	P42336	1	2.45	1.33	1.17	0.94
	I6Y9J2	1	3.02	1.25	1.62	0.79
	O75909	1	1.78	2.61	0.93	2.65
Ligase	Q15819	1	1.23	0.26	1.08	0.19
	P46934	1	1.25	3.91	0.84	1.41
	Q4DA54	1	1.44	1.04	0.78	1.47
Lyase	P07805	1	0.90	1.17	1.19	1.34
Oxidoreductase	Q4D3W2	2	1.57	0.75	3.82	0.73
	P10599	1	1.08	3.72	2.80	1.84
	Q13162	1	0.94	1.85	2.57	2.80
	P30838	1	1.01	0.64	1.28	1.27
	P49419	1	1.36	1.05	2.17	0.90
Isomerase	P44859	2	1.14	0.40	0.99	2.99
	Q46822	2	1.50	1.49	3.31	2.25
	Q9LFG2	2	1.19	1.15	0.88	3.07
Transcription	P37231	10	1.47	2.86	2.93	1.97
	P30822	4	1.79	4.63	1.31	1.49
	Q14145	3	1.52	2.40	0.95	2.66
	Q99AU2	1	1.99	1.89	2.47	2.16
Viral Protein	P27958	2	1.49	0.70	1.17	5.90
	Q91RS4	1	4.17	0.90	1.91	5.27
	O39914	1	8.42	4.58	1.32	2.84
Metal binding protein	P02638	5	1.04	4.54	3.00	1.67

Figure S3. Median RMSD value of *Best Sampled Pose* on each target. *n* stands for the total number of complexes for a given uniprotID.

Table S4. The number of best RMSD value cross all the docking tools for a given tool based on the receptor type for *Best Sampled Pose*.

Receptor Type	MOE	GOLD	CovDock	ICM-Pro
Hydrolase	14	9	18	12
Transferase	9	2	8	12
Ligase	0	0	2	1
Lyase	1	0	0	0
Oxidoreductase	2	1	0	2
Isomerase	0	2	1	0
Transcription	1	1	2	0
Viral Protein	0	2	1	0
Metal binding protein	1	0	0	0
Total	28	17	32	27

Table S5. The generalities calculated by the generality(*G(i,x)*) formula for four docking tool with *Best Sampled Pose* measurement based on the receptor type**.**

Receptor Type	MOE	GOLD	CovDock	ICM-Pro
Hydrolase	6.9	4.4	8.8	5.9
Transferase	10.8	2.4	9.6	14.5
Ligase	0	0	66.7	33.3
Lyase	100	0	0	0
Oxidoreductase	33.3	16.7	0	33.3
Isomerase	0	33.3	16.7	0
Transcription	5.6	5.6	11.1	0
Viral Protein	0	50	25	0
Metal binding protein	20	0	0	0
G(i,x)	176.6	112.4	137.9	87

Table S6. The number of best RMSD value cross all the docking tools for a given tool based on the warhead type for *Best Scored Pose*.

Warhead Type	MOE	GOLD	CovDock	ICM-Pro
Nitrile	0	1	0	0
Alkene	1	0	1	1
Carbonyl	2	1	0	0
Alkyne	0	0	1	0
Guanyl	0	0	1	0
Halide	1	0	0	1
Others	0	1	1	0
Heterocyclic	1	1	0	0
Sulfydryl	1	0	0	0
Nitrile	0	0	1	0
Carbonyl	1	0	0	1
Boronic Acid	1	0	0	0
Phosphonyl	0	0	0	1
Lactam	1	0	0	1
lactone	1	0	0	0
Total	10	4	5	5

Table S7. The generalities calculated by the generality(*G(i,x)*) formula for four docking tool with *Best Scored Pose* measurement based on the warhead type.

Warhead Type	MOE	GOLD	CovDock	ICM-Pro
Nitrile	0.0	2.4	0.0	0.0
Alkene	0.9	0.0	0.9	0.9
Carbonyl	8.0	4.0	0.0	0.0
Alkyne	0.0	0.0	100.0	0.0
Guanyl	0.0	0.0	50.0	0.0
Halide	6.7	0.0	0.0	6.7
Others	0.0	6.3	6.3	0.0
Heterocyclic	5.9	5.9	0.0	0.0
Sulfydryl	10.0	0.0	0.0	0.0
Nitrile	0.0	0.0	33.3	0.0

Carbonyl	3.4	0.0	0.0	3.4
Boronic Acid	12.5	0.0	0.0	0.0
Phosphonyl	0.0	0.0	0.0	33.3
Lactam	2.5	0.0	0.0	2.5
lactone	50.0	0.0	0.0	0.0
G(i,x)	99.9	18.5	190.4	46.8

Table S8. The number of best RMSD value cross all the docking tools for a given tool based on the warhead type for *Best Sampled Pose*.

Warhead Type	MOE	GOLD	CovDock	ICM-Pro
Nitrile	0	1	0	0
Alkene	1	0	1	1
Carbonyl	0	0	2	1
Alkyne	0	0	1	0
Guanyl	0	0	1	0
Halide	0	0	0	2
Others	0	1	1	0
Heterocyclic	1	1	0	0
Sulfydryl	1	0	0	0
Nitrile	0	0	1	0
Carbonyl	1	0	0	1
Boronic Acid	1	0	0	0
Phosphonyl	1	0	0	0
Lactam	0	0	1	1
lactone	1	0	0	0
Total	7	3	8	6

Table S9. The generalities calculated by the generality(*G(i,x)*) formula for four docking tool with *Best Sampled Pose* measurement based on the warhead type.

Warhead Type	MOE	GOLD	CovDock	ICM-Pro
Nitrile	0.0	2.4	0.0	0.0
Alkene	0.9	0.0	0.9	0.9
Carbonyl	0.0	0.0	8.0	4.0
Alkyne	0.0	0.0	100.0	0.0
Guanyl	0.0	0.0	50.0	0.0
Halide	0.0	0.0	0.0	13.3
Others	0.0	6.3	6.3	0.0
Heterocyclic	5.9	5.9	0.0	0.0
Sulfydryl	10.0	0.0	0.0	0.0
Nitrile	0.0	0.0	33.3	0.0
Carbonyl	3.4	0.0	0.0	3.4
Boronic Acid	12.5	0.0	0.0	0.0
Phosphonyl	33.3	0.0	0.0	0.0
Lactam	0.0	0.0	2.5	2.5
lactone	50.0	0.0	0.0	0.0
G(i,x)	116.0	14.5	200.9	24.1

Table S10. Mann-Whitney U-test results of the difference between the *Best Scored Pose* and *Best Sampled Pose* RMSD sets on each of four docking tools.

MOE	GOLD	CovDock	ICM-Pro
3.49E-20	1.39E-06	8.50E-05	1.05E-05

Table S11. Mann-Whitney U-test results of the difference between RMSD sets of the docking tools on *Best Scored Pose* and *Best Sampled Pose* on measurement.

Best Scored Pose	0.006
Best Sampled Pose	0.069

Table S12. The robustness analysis results for the four docking tools.

Tool	Average and Standard Deviation of the RMSD(Å)	
	Best Scored Pose	Best Sampled Pose
MOE	2.27±1.21	1.66±0.91
GOLD	3.18±2.6	2.18±1.74
CovDock	2.71±2.61	2.05±2.18
ICM-Pro	3.41±3.6	2.36±2.84

Table S13. The *P-deviations*, *S-counts*, and *F-counts* for docking tools.

PDB	S_1	S_2	MOE			GOLD			CovDOCK			ICM-Pro		
			P-deviation	S-count	F-count	P-deviation	S-count	F-count	P-deviation	S-count	F-count	P-deviation	S-count	F-count
1atk	1.49	1.49	0.00	1	0	0.25	1.00	0.00	0.05	1.00	0.00	0.00	0.00	1.00
1au0	2.85	1.70	1.15	0	0	3.64	0.00	0.00	0.25	0.00	1.00	0.00	0.00	1.00
1au2	3.94	2.60	1.34	0	1	0.00	0.00	1.00	0.00	1.00	0.00	5.74	0.00	1.00
1au3	6.50	6.50	0.00	0	1	0.00	1.00	0.00	0.14	1.00	0.00	1.44	0.00	1.00
1awf	1.43	1.43	0.00	1	0	0.00	1.00	0.00	0.00	1.00	0.00	0.00	0.00	1.00
1ayu	3.25	2.68	0.57	0	1	1.33	0.00	0.00	5.48	0.00	0.00	0.00	0.00	1.00
1ayv	3.41	3.41	0.00	0	1	0.00	0.00	1.00	0.00	0.00	1.00	1.50	0.00	1.00
1ayw	1.30	1.05	0.26	1	0	2.79	0.00	1.00	0.00	0.00	1.00	0.82	0.00	1.00
1bgo	3.23	1.61	1.62	0	0	0.08	1.00	0.00	0.00	1.00	0.00	10.10	0.00	0.00
1cef	2.67	1.79	0.88	0	0	1.71	0.00	0.00	0.40	0.00	1.00	1.13	0.00	0.00
1ceg	1.26	1.06	0.20	1	0	0.02	1.00	0.00	0.00	1.00	0.00	0.00	1.00	0.00
1cqq	2.48	1.54	0.94	0	0	0.00	1.00	0.00	0.21	1.00	0.00	2.25	0.00	1.00
1csb	1.37	1.37	0.00	1	0	0.08	1.00	0.00	0.50	1.00	0.00	0.79	0.00	0.00
1f1j	2.78	1.48	1.30	0	0	1.91	0.00	1.00	0.13	1.00	0.00	0.00	1.00	0.00
1fh0	1.99	1.74	0.24	1	0	0.20	1.00	0.00	6.80	0.00	1.00	0.00	1.00	0.00
1gmy	2.31	1.77	0.54	0	0	0.09	1.00	0.00	0.00	1.00	0.00	0.00	1.00	0.00
1h8i	5.53	2.31	3.22	0	1	0.25	0.00	1.00	4.76	0.00	0.00	13.39	0.00	1.00
1haz	3.49	3.49	0.00	0	1	0.23	0.00	1.00	5.73	0.00	0.00	0.00	1.00	0.00
1hvb	2.35	1.44	0.91	0	0	1.23	0.00	1.00	1.86	0.00	1.00	0.00	0.00	1.00
1hvy	1.70	1.42	0.29	1	0	0.55	0.00	0.00	0.00	1.00	0.00	0.00	1.00	0.00
1iau	1.84	1.63	0.20	1	0	3.78	0.00	1.00	0.48	0.00	1.00	0.00	1.00	0.00
1ju6	0.71	0.71	0.00	1	0	0.31	0.00	1.00	0.00	1.00	0.00	0.00	1.00	0.00
1khp	1.77	1.77	0.00	1	0	0.00	1.00	0.00	0.00	1.00	0.00	0.36	1.00	0.00
1khq	2.41	1.41	0.99	0	0	0.07	0.00	1.00	0.79	1.00	0.00	0.88	0.00	0.00
1ms6	2.32	2.10	0.22	0	1	0.51	1.00	0.00	0.00	1.00	0.00	3.47	0.00	0.00
1mt5	2.33	1.54	0.79	0	0	0.34	1.00	0.00	0.00	0.00	1.00	0.00	1.00	0.00
1nfz	1.38	1.38	0.00	1	0	1.12	1.00	0.00	0.00	1.00	0.00	0.00	0.00	1.00
1nl6	2.27	2.25	0.01	0	1	0.00	0.00	1.00	0.04	1.00	0.00	0.37	1.00	0.00
1nlj	6.34	6.23	0.11	0	1	0.00	1.00	0.00	1.54	0.00	0.00	1.61	0.00	0.00
1nms	1.49	1.49	0.00	1	0	0.05	1.00	0.00	0.00	1.00	0.00	0.00	1.00	0.00
1npz	2.49	2.14	0.35	0	1	0.18	0.00	1.00	0.00	1.00	0.00	0.00	1.00	0.00
1oce	1.46	1.26	0.20	1	0	1.29	0.00	1.00	0.00	0.00	1.00	0.00	0.00	1.00
1pw8	2.37	1.94	0.44	0	0	1.48	0.00	0.00	0.50	1.00	0.00	1.97	0.00	0.00
1pwc	2.37	1.82	0.55	0	0	0.33	1.00	0.00	0.09	1.00	0.00	0.00	1.00	0.00
1pwg	2.28	2.21	0.07	0	1	0.00	1.00	0.00	0.15	1.00	0.00	0.00	1.00	0.00
1pyo	1.47	1.41	0.06	1	0	2.59	0.00	1.00	0.08	1.00	0.00	0.00	1.00	0.00
1qhr	1.49	1.15	0.34	1	0	0.29	0.00	1.00	0.00	1.00	0.00	0.00	1.00	0.00
1qj1	1.01	1.01	0.00	1	0	0.06	1.00	0.00	0.17	1.00	0.00	0.00	1.00	0.00
1qj6	1.44	1.08	0.36	1	0	0.00	0.00	1.00	2.70	0.00	0.00	1.24	0.00	0.00
1qj7	2.48	1.75	0.73	0	0	5.40	0.00	0.00	8.06	0.00	0.00	5.81	0.00	0.00
1qtn	1.58	1.36	0.22	1	0	0.00	0.00	1.00	0.00	1.00	0.00	7.19	0.00	0.00
1rtl	8.42	8.42	0.00	0	1	4.29	0.00	1.00	6.02	0.00	0.00	0.00	0.00	1.00
1scw	1.39	1.39	0.00	1	0	1.44	0.00	1.00	0.00	0.00	1.00	0.00	1.00	0.00
1u9v	2.27	2.22	0.05	0	1	0.02	0.00	1.00	0.06	0.00	1.00	0.34	0.00	1.00
1u9w	3.04	1.18	1.87	0	0	0.17	1.00	0.00	0.00	0.00	1.00	6.00	0.00	0.00
1u9x	4.24	3.79	0.46	0	1	4.47	0.00	1.00	0.00	1.00	0.00	5.46	0.00	0.00
1vm1	1.73	1.31	0.42	1	0	2.33	0.00	1.00	0.00	0.00	1.00	0.00	0.00	1.00
1w8y	1.06	0.54	0.52	1	0	5.75	0.00	0.00	1.70	0.00	1.00	0.00	1.00	0.00
1x83	3.29	1.63	1.66	0	0	0.00	0.00	1.00	0.00	0.00	1.00	0.00	1.00	0.00
1yqs	1.67	1.47	0.20	1	0	0.40	0.00	1.00	0.00	0.00	1.00	0.00	1.00	0.00
1z6f	4.46	4.18	0.27	0	1	2.07	0.00	1.00	0.00	0.00	1.00	2.13	0.00	1.00
1zlr	1.74	1.50	0.24	1	0	0.12	1.00	0.00	1.35	0.00	0.00	0.00	1.00	0.00
1zpz	5.37	2.75	2.61	0	1	1.25	0.00	1.00	5.28	0.00	1.00	2.84	0.00	1.00
1zsl	2.00	1.69	0.30	1	0	4.57	0.00	0.00	6.55	0.00	0.00	0.00	1.00	0.00
2a4q	4.89	4.17	0.72	0	1	0.00	1.00	0.00	1.19	0.00	0.00	0.00	0.00	1.00
2a5k	3.43	1.45	1.98	0	0	0.26	1.00	0.00	0.30	1.00	0.00	2.25	0.00	1.00
2awz	1.49	1.20	0.28	1	0	1.61	0.00	1.00	0.00	0.00	1.00	0.00	1.00	0.00
2ax0	2.78	1.57	1.21	0	0	0.78	0.00	1.00	1.03	1.00	0.00	0.21	0.00	0.00
2ax1	0.81	0.77	0.05	1	0	0.00	0.00	1.00	0.67	0.00	1.00	0.02	0.00	1.00
2c1e	2.21	1.79	0.43	0	0	0.43	0.00	1.00	0.58	0.00	0.00	3.34	0.00	1.00

2c2k	2.56	2.56	0.00	0	1	1.65	0.00	1.00	2.16	0.00	0.00	0.98	0.00	1.00
2c2o	3.15	2.99	0.16	0	1	0.71	0.00	0.00	0.45	0.00	0.00	0.00	0.00	1.00
2c2z	2.80	1.64	1.16	0	0	0.07	0.00	0.00	8.66	0.00	0.00	0.00	0.00	1.00
2cdr	3.05	1.49	1.57	0	0	3.17	0.00	1.00	1.37	0.00	1.00	3.02	0.00	1.00
2cnk	3.07	2.46	0.61	0	1	2.36	0.00	1.00	0.86	0.00	0.00	2.64	0.00	1.00
2cnl	2.32	1.97	0.34	0	0	1.44	0.00	1.00	0.64	0.00	0.00	0.00	0.00	1.00
2cnn	3.13	1.98	1.15	0	0	1.44	0.00	1.00	0.03	0.00	1.00	0.00	0.00	1.00
2cno	2.93	1.76	1.17	0	0	0.45	0.00	1.00	3.87	0.00	1.00	0.51	0.00	1.00
2ex6	0.98	0.69	0.29	1	0	0.08	0.00	1.00	1.77	0.00	0.00	3.09	0.00	0.00
2ex8	2.14	1.39	0.75	0	0	0.89	0.00	1.00	0.00	1.00	0.00	0.00	1.00	0.00
2ex9	1.65	1.65	0.00	1	0	0.00	1.00	0.00	0.00	1.00	0.00	0.00	1.00	0.00
2exb	1.67	1.62	0.05	1	0	0.51	0.00	1.00	0.03	0.00	1.00	0.00	0.00	1.00
2f9a	1.51	1.51	0.00	1	0	0.34	0.00	0.00	0.58	0.00	1.00	0.00	0.00	1.00
2fq9	2.74	1.28	1.46	0	0	0.00	1.00	0.00	0.00	1.00	0.00	2.36	0.00	0.00
2fra	1.86	1.33	0.53	1	0	0.00	1.00	0.00	0.15	1.00	0.00	1.52	0.00	1.00
2frq	3.96	2.07	1.89	0	1	1.35	0.00	1.00	0.00	1.00	0.00	1.51	0.00	1.00
2ft2	1.71	1.57	0.14	1	0	0.00	1.00	0.00	0.22	1.00	0.00	0.00	1.00	0.00
2fud	1.57	1.57	0.00	1	0	0.00	1.00	0.00	0.00	1.00	0.00	0.00	1.00	0.00
2fye	2.25	2.25	0.00	0	1	0.59	1.00	0.00	0.00	1.00	0.00	0.00	1.00	0.00
2g6d	3.46	1.46	2.01	0	0	0.41	1.00	0.00	0.52	0.00	0.00	0.16	0.00	1.00
2gke	1.30	1.30	(0.00)	1	0	0.00	1.00	0.00	0.01	1.00	0.00	0.00	0.00	1.00
2gkj	1.60	0.97	0.63	1	0	0.00	1.00	0.00	0.00	1.00	0.00	0.00	1.00	0.00
2h5s	1.70	1.59	0.11	1	0	0.02	0.00	1.00	0.00	0.00	1.00	1.10	0.00	1.00
2hwp	2.54	1.79	0.74	0	0	3.99	0.00	1.00	0.00	0.00	1.00	0.00	0.00	1.00
2i03	2.39	1.64	0.75	0	0	0.00	1.00	0.00	0.94	1.00	0.00	0.00	0.00	1.00
2ijn	2.44	1.99	0.45	0	0	5.81	0.00	0.00	0.00	0.00	1.00	3.82	0.00	1.00
2ipp	2.65	2.65	0.00	0	1	0.81	0.00	1.00	0.00	0.00	1.00	0.66	0.00	1.00
2j8y	1.68	0.95	0.73	1	0	0.01	1.00	0.00	0.40	1.00	0.00	0.00	1.00	0.00
2jai	1.10	0.64	0.46	1	0	0.01	1.00	0.00	0.00	1.00	0.00	0.07	1.00	0.00
2jbf	1.46	0.98	0.49	1	0	0.30	1.00	0.00	0.00	1.00	0.00	1.31	0.00	0.00
2mlm	2.68	2.68	0.00	0	1	0.00	0.00	1.00	0.00	1.00	0.00	0.00	1.00	0.00
2oc0	1.77	1.77	0.00	1	0	0.01	1.00	0.00	4.42	0.00	0.00	0.00	0.00	1.00
2oc1	2.93	1.21	1.72	0	0	0.00	1.00	0.00	0.00	1.00	0.00	4.33	0.00	1.00
2op3	2.84	1.29	1.54	0	0	0.00	1.00	0.00	0.00	1.00	0.00	4.30	0.00	0.00
2pre	1.58	1.58	0.00	1	0	0.17	0.00	1.00	0.00	1.00	0.00	0.00	0.00	1.00
2px6	2.74	1.63	1.12	0	0	1.93	0.00	0.00	1.84	0.00	0.00	1.37	0.00	1.00
2qlq	2.45	1.01	1.44	0	0	0.19	1.00	0.00	0.00	1.00	0.00	0.00	1.00	0.00
2qq7	1.40	1.29	0.11	1	0	0.14	1.00	0.00	0.28	1.00	0.00	2.48	0.00	1.00
2r4b	2.01	1.90	0.11	0	0	0.00	1.00	0.00	0.07	1.00	0.00	0.00	1.00	0.00
2r6n	1.48	1.35	0.13	1	0	0.02	1.00	0.00	0.90	1.00	0.00	2.23	0.00	0.00
2r9m	2.05	1.81	0.24	0	0	0.00	1.00	0.00	0.00	1.00	0.00	2.25	0.00	0.00
2r9n	2.25	1.81	0.44	0	0	0.24	1.00	0.00	0.00	1.00	0.00	0.00	1.00	0.00
2r9o	4.39	2.07	2.32	0	1	1.54	0.00	0.00	0.00	0.00	1.00	0.00	0.00	1.00
2tod	0.96	0.90	0.07	1	0	0.06	1.00	0.00	0.05	1.00	0.00	0.00	1.00	0.00
2v35	1.53	1.40	0.13	1	0	0.00	1.00	0.00	3.22	0.00	0.00	7.21	0.00	0.00
2vgj	1.82	1.81	0.01	1	0	1.11	1.00	0.00	0.14	1.00	0.00	0.00	1.00	0.00
2wap	1.04	0.80	0.24	1	0	0.00	1.00	0.00	0.00	1.00	0.00	0.00	1.00	0.00
2wke	1.37	0.88	0.49	1	0	0.01	0.00	1.00	1.41	0.00	1.00	0.00	0.00	1.00
2xk1	1.94	0.85	1.09	1	0	0.69	0.00	0.00	0.00	0.00	1.00	0.00	0.00	1.00
2xln	2.07	2.07	0.00	0	1	0.01	1.00	0.00	0.61	0.00	1.00	0.00	0.00	1.00
2xu1	2.08	1.75	0.33	0	0	0.15	1.00	0.00	0.00	0.00	1.00	6.30	0.00	1.00
2xu3	1.71	1.71	0.00	1	0	1.26	0.00	1.00	0.00	1.00	0.00	3.86	0.00	0.00
2xu4	2.27	1.29	0.97	0	0	0.06	1.00	0.00	0.00	1.00	0.00	0.00	1.00	0.00
2xu5	1.82	1.82	0.00	1	0	0.05	1.00	0.00	0.00	1.00	0.00	3.71	0.00	1.00
2y4a	4.37	1.01	3.36	0	0	0.05	1.00	0.00	0.00	0.00	1.00	0.00	1.00	0.00
2y59	1.78	1.77	0.00	1	0	0.25	0.00	1.00	0.00	0.00	1.00	0.20	0.00	1.00
2yj2	5.91	2.68	3.22	0	1	0.22	1.00	0.00	0.00	0.00	1.00	0.00	0.00	1.00
2yj8	3.65	2.04	1.61	0	1	0.05	1.00	0.00	0.00	1.00	0.00	2.46	0.00	1.00
2yj9	5.12	5.12	0.00	0	1	0.00	1.00	0.00	0.00	1.00	0.00	4.77	0.00	0.00
2yjb	4.32	4.28	0.03	0	1	0.02	1.00	0.00	0.00	1.00	0.00	0.00	0.00	1.00
2yjc	2.00	1.23	0.77	0	0	0.10	1.00	0.00	0.00	1.00	0.00	1.41	0.00	1.00
2zk1	2.61	1.92	0.69	0	0	2.77	0.00	1.00	5.72	0.00	1.00	4.62	0.00	1.00
2zk2	2.60	2.10	0.50	0	1	0.94	0.00	1.00	4.24	0.00	1.00	2.06	0.00	1.00
2zk3	2.28	1.55	0.74	0	0	4.51	0.00	1.00	0.00	0.00	1.00	3.27	0.00	1.00
2zk4	2.32	1.13	1.19	0	0	3.90	0.00	1.00	3.10	0.00	1.00	0.00	0.00	1.00
2zk5	1.71	0.72	0.99	1	0	0.00	1.00	0.00	0.02	1.00	0.00	0.00	1.00	0.00
3a3e	2.39	1.64	0.74	0	0	0.64	0.00	1.00	0.09	0.00	1.00	1.10	0.00	0.00
3a3f	2.48	1.47	1.02	0	0	1.17	0.00	0.00	0.36	1.00	0.00	4.61	0.00	0.00
3a3i	1.51	1.32	0.19	1	0	2.17	0.00	0.00	0.00	1.00	0.00	1.80	0.00	0.00
3b0r	1.73	0.70	1.03	1	0	0.00	1.00	0.00	0.00	0.00	1.00	0.00	1.00	0.00
3bcn	2.20	1.03	1.17	0	0	0.12	0.00	1.00	0.27	0.00	1.00	0.00	0.00	1.00
3beb	0.85	0.85	0.00	1	0	0.01	1.00	0.00	0.00	1.00	0.00	0.83	0.00	0.00
3bfc	1.03	1.03	0.00	1	0	0.20	0.00	1.00	5.21	0.00	0.00	0.80	0.00	0.00
3bff	1.73	1.02	0.71	1	0	1.60	0.00	0.00	0.53	1.00	0.00	1.72	0.00	0.00
3bfg	3.58	1.83	1.75	0	0	0.25	0.00	0.00	0.00	1.00	0.00	6.62	0.00	1.00
3bg8	1.07	1.07	0.00	1	0	4.69	0.00	1.00	0.00	1.00	0.00	0.00	0.00	1.00
3bjm	1.24	1.02	0.22	1	0	0.78	0.00	0.00	0.00	1.00	0.00	0.00	1.00	0.00
3blt	1.40	1.40	0.00	1	0	0.04	1.00	0.00	0.00	1.00	0.00	0.00	1.00	0.00
3blu	1.80	1.12	0.68	1	0	0.06	1.00	0.00	0.00	1.00	0.00	0.00	1.00	0.00
3bm8	1.79	1.79	0.00	1	0	0.23	1.00	0.00	0.00	1.00	0.00	0.00	1.00	0.00
3bpf	1.93	1.43	0.50	1	0	0.00	0.00	1.00	1.97	0.00	0.00	3.42	0.00	1.00
3bpm	1.72	1.39	0.33	1	0	0.00	1.00	0.00	3.08	0.00	0.00	1.66	0.00	0.00
3bwk	5.80	2.01	3.79	0	1	0.02	0.00	1.00	0.00	1.00	0.00	0.00	0.00	1.00
3cg5	1.60	1.22	0.39	1	0	2.38	0.00	0.00	0.00	1.00	0.00	1.74	0.00	0.00
3d4f	2.20	1.44	0.76	0	0	0.18	0.00	1.00	0.25	0.00	1.00	0.29	0.00	1.00
3e0n	2.00	1.76	0.23	1	0	2.46	0.00	1.00	0.45	0.00	1.00	1.97	0.00	0.00
3e0p	3.79	3.79	0.00	0	1	6.49	0.00	1.00	0.00	0.00	1.00	3.06	0.00	1.00

3ejx	1.32	1.00	0.32	1	0	0.00	1.00	0.00	0.00	1.00	0.00	0.00	0.00	1.00
3ekm	1.71	1.37	0.34	1	0	0.11	1.00	0.00	0.00	1.00	0.00	0.00	1.00	0.00
3fv7	2.86	1.56	1.29	0	0	0.00	0.00	1.00	0.68	0.00	1.00	0.26	0.00	1.00
3fvf	1.03	0.92	0.11	1	0	0.00	0.00	1.00	0.43	1.00	0.00	0.00	1.00	0.00
3fzc	1.64	1.64	0.00	1	0	0.01	0.00	1.00	0.89	0.00	1.00	1.28	0.00	1.00
3hgn	2.35	1.36	0.99	0	0	0.00	0.00	1.00	0.00	1.00	0.00	0.00	1.00	0.00
3hha	1.60	1.59	0.00	1	0	0.00	1.00	0.00	0.00	1.00	0.00	0.00	1.00	0.00
3hwn	6.97	6.97	0.00	0	1	0.00	1.00	0.00	0.00	1.00	0.00	1.46	0.00	1.00
3i4a	1.57	1.39	0.18	1	0	0.29	1.00	0.00	0.00	1.00	0.00	0.00	1.00	0.00
3ika	2.59	1.09	1.50	0	0	3.38	0.00	1.00	0.00	1.00	0.00	0.00	0.00	1.00
3k84	2.02	1.37	0.66	0	0	4.42	0.00	0.00	0.00	1.00	0.00	0.00	1.00	0.00
3kjf	2.39	2.39	0.00	0	1	0.23	1.00	0.00	1.34	0.00	0.00	0.00	1.00	0.00
3kjn	2.10	2.10	0.00	0	1	0.18	1.00	0.00	0.95	1.00	0.00	0.00	0.00	1.00
3kjq	2.08	2.08	0.00	0	1	0.00	1.00	0.00	0.00	1.00	0.00	2.55	0.00	0.00
3kw9	1.58	1.41	0.17	1	0	0.10	1.00	0.00	0.28	1.00	0.00	6.36	0.00	0.00
3kwb	5.99	5.84	0.15	0	1	0.03	1.00	0.00	0.00	1.00	0.00	5.29	0.00	1.00
3kwz	2.56	1.31	1.25	0	0	6.73	0.00	0.00	0.00	1.00	0.00	4.11	0.00	1.00
3LJ6	2.41	1.39	1.02	0	0	0.46	1.00	0.00	0.00	1.00	0.00	0.00	1.00	0.00
3lok	0.66	0.66	0.00	1	0	3.32	0.00	1.00	0.00	1.00	0.00	1.35	0.00	0.00
3mzd	1.61	1.43	0.18	1	0	0.02	0.00	1.00	0.52	1.00	0.00	0.15	0.00	1.00
3mze	1.07	1.07	0.00	1	0	1.10	0.00	0.00	0.00	0.00	1.00	0.00	1.00	0.00
3n4c	3.27	3.27	0.00	0	1	0.30	1.00	0.00	0.00	1.00	0.00	2.30	0.00	0.00
3o0u	2.04	1.02	1.02	0	0	0.06	1.00	0.00	0.00	1.00	0.00	0.00	1.00	0.00
3o1g	0.97	0.97	0.00	1	0	0.00	1.00	0.00	4.34	0.00	1.00	0.00	1.00	0.00
3of8	1.94	1.89	0.04	1	0	0.52	0.00	1.00	0.00	1.00	0.00	9.53	0.00	0.00
3of9	2.63	2.63	0.00	0	1	0.83	0.00	1.00	0.00	0.00	1.00	8.25	0.00	1.00
3orx	1.00	0.90	0.09	1	0	0.36	0.00	1.00	0.00	1.00	0.00	0.00	0.00	1.00
3orz	1.41	1.41	0.00	1	0	0.00	1.00	0.00	0.00	1.00	0.00	0.00	1.00	0.00
3otu	1.60	1.59	0.00	1	0	0.71	0.00	0.00	0.00	0.00	1.00	1.07	0.00	0.00
3ovx	1.87	1.87	0.01	1	0	0.00	1.00	0.00	2.94	0.00	0.00	8.58	0.00	0.00
3p8e	1.98	1.37	0.62	1	0	0.67	1.00	0.00	0.00	1.00	0.00	0.00	1.00	0.00
3pae	1.52	1.52	0.00	1	0	0.08	1.00	0.00	0.07	0.00	1.00	0.00	1.00	0.00
3s3j	2.05	2.05	0.00	0	1	0.17	0.00	1.00	0.00	0.00	1.00	0.00	1.00	0.00
3s3p	2.05	2.05	0.00	0	1	11.84	0.00	0.00	0.00	1.00	0.00	4.87	0.00	0.00
3s3q	1.60	1.52	0.08	1	0	0.00	0.00	1.00	0.00	1.00	0.00	0.00	1.00	0.00
3s3s	2.25	2.25	0.00	0	1	1.92	0.00	1.00	0.00	1.00	0.00	0.46	0.00	0.00
3svv	1.87	1.23	0.64	1	0	0.00	1.00	0.00	0.99	1.00	0.00	0.00	1.00	0.00
3szb	1.45	1.01	0.44	1	0	1.03	1.00	0.00	0.00	1.00	0.00	0.00	1.00	0.00
3t9t	1.10	1.10	0.00	1	0	0.00	1.00	0.00	0.17	1.00	0.00	0.00	1.00	0.00
3tyq	2.30	1.50	0.80	0	0	0.00	0.00	1.00	0.00	0.00	1.00	0.00	1.00	0.00
3u1i	4.28	2.30	1.97	0	1	4.86	0.00	1.00	1.92	0.00	1.00	0.00	0.00	1.00
3upn	2.29	2.27	0.01	0	1	0.18	0.00	1.00	0.60	0.00	1.00	0.00	0.00	1.00
3upo	2.62	2.10	0.52	0	1	1.60	0.00	0.00	0.97	0.00	0.00	0.16	0.00	1.00
3upp	1.86	1.06	0.79	1	0	0.22	1.00	0.00	0.30	1.00	0.00	0.00	0.00	1.00
3v4o	2.38	2.38	0.00	0	1	4.75	0.00	1.00	0.00	1.00	0.00	0.00	1.00	0.00
3v6r	2.02	1.76	0.26	0	0	1.59	0.00	0.00	0.15	1.00	0.00	0.00	0.00	1.00
3w2q	1.99	1.40	0.60	1	0	0.09	1.00	0.00	0.31	0.00	0.00	0.00	1.00	0.00
3w2t	1.38	1.34	0.04	1	0	0.54	0.00	1.00	0.00	1.00	0.00	0.00	0.00	1.00
3x1i	1.92	1.53	0.38	1	0	5.28	0.00	1.00	0.00	1.00	0.00	3.07	0.00	1.00
3zcz	0.91	0.76	0.15	1	0	0.00	1.00	0.00	0.42	1.00	0.00	0.00	1.00	0.00
3zfz	1.92	1.92	0.00	1	0	0.00	1.00	0.00	0.00	1.00	0.00	1.52	0.00	0.00
3zim	2.78	2.45	0.33	0	1	0.86	0.00	0.00	0.00	1.00	0.00	0.00	1.00	0.00
4axm	2.39	1.20	1.19	0	0	0.14	0.00	1.00	0.00	1.00	0.00	0.00	1.00	0.00
4b4x	1.57	1.36	0.22	1	0	2.11	0.00	0.00	0.00	1.00	0.00	0.00	1.00	0.00
4ben	1.49	0.98	0.52	1	0	0.22	0.00	1.00	3.19	0.00	1.00	0.00	0.00	1.00
4d9u	0.80	0.80	0.00	1	0	0.93	0.00	1.00	0.02	1.00	0.00	0.00	1.00	0.00
4dki	4.05	1.91	2.14	0	0	0.64	0.00	1.00	0.41	0.00	1.00	0.00	1.00	0.00
4dmx	6.02	2.35	3.67	0	1	0.31	1.00	0.00	0.00	1.00	0.00	4.24	0.00	0.00
4dmy	1.54	1.54	0.00	1	0	2.48	0.00	1.00	0.00	1.00	0.00	2.52	0.00	0.00
4g5j	2.20	1.47	0.72	0	0	1.41	0.00	0.00	0.00	1.00	0.00	0.00	1.00	0.00
4gs6	2.84	2.08	0.76	0	1	2.13	0.00	1.00	4.63	0.00	0.00	0.00	1.00	0.00
4hav	5.24	2.14	3.10	0	1	1.88	0.00	1.00	1.14	0.00	0.00	1.55	0.00	0.00
4hax	4.84	1.75	3.08	0	0	0.00	0.00	1.00	0.42	1.00	0.00	1.72	0.00	0.00
4hay	1.59	1.59	0.00	1	0	0.00	0.00	1.00	0.64	0.00	0.00	0.53	0.00	1.00
4haz	3.24	1.83	1.40	0	0	10.23	0.00	1.00	0.62	1.00	0.00	0.00	1.00	0.00
4hct	1.42	1.42	0.00	1	0	3.74	0.00	0.00	0.00	1.00	0.00	0.00	1.00	0.00
4hcu	1.06	0.87	0.19	1	0	0.00	1.00	0.00	0.00	1.00	0.00	0.00	1.00	0.00
4i24	1.42	0.96	0.46	1	0	0.53	1.00	0.00	0.00	1.00	0.00	0.00	1.00	0.00
4jj7	1.87	1.36	0.51	1	0	0.00	0.00	1.00	0.93	0.00	1.00	2.72	0.00	1.00
4jj8	2.43	1.59	0.83	0	0	0.96	0.00	0.00	1.89	0.00	1.00	0.24	0.00	1.00
4jje	2.92	1.71	1.21	0	0	0.82	0.00	0.00	0.14	0.00	1.00	0.00	0.00	1.00
4kty	4.86	2.97	1.88	0	1	0.00	0.00	1.00	0.64	0.00	1.00	1.31	0.00	1.00
4li5	2.04	1.78	0.26	0	0	0.00	0.00	1.00	0.00	1.00	0.00	0.00	1.00	0.00
4lqm	1.38	1.31	0.07	1	0	0.31	1.00	0.00	0.00	1.00	0.00	0.00	1.00	0.00
4lv6	2.97	1.22	1.76	0	0	0.29	1.00	0.00	0.00	1.00	0.00	2.08	0.00	0.00
4lyf	2.15	1.18	0.97	0	0	1.40	0.00	0.00	1.15	0.00	0.00	0.00	1.00	0.00
4lyj	2.39	1.41	0.98	0	0	2.10	0.00	0.00	0.00	1.00	0.00	0.00	0.00	1.00
4m1o	1.15	1.15	0.00	1	0	0.62	1.00	0.00	0.00	1.00	0.00	0.06	1.00	0.00
4m1s	1.13	1.13	0.00	1	0	0.00	1.00	0.00	0.00	1.00	0.00	0.02	1.00	0.00
4m1t	2.14	2.14	0.00	0	1	0.12	1.00	0.00	0.00	1.00	0.00	0.42	1.00	0.00
4m1w	5.07	3.40	1.66	0	1	0.06	0.00	1.00	0.00	0.00	1.00	0.71	0.00	1.00
4m21	1.37	0.77	0.60	1	0	2.58	0.00	0.00	0.00	1.00	0.00	0.00	0.00	1.00
4m22	2.11	1.11	1.00	0	0	1.12	0.00	1.00	0.00	0.00	1.00	9.62	0.00	1.00
4mz4	0.87	0.87	0.00	1	0	4.90	0.00	1.00	0.00	0.00	1.00	0.00	1.00	0.00
4mzo	4.60	1.83	2.78	0	0	0.27	1.00	0.00	0.00	1.00	0.00	0.00	1.00	0.00
4onm	2.90	1.23	1.67	0	0	0.00	1.00	0.00	0.00	1.00	0.00	0.00	1.00	0.00
4pdz	2.76	1.81	0.95	0	0	0.00	0.00	1.00	0.94	0.00	1.00	0.00	0.00	1.00

4pe0	1.72	1.72	0.00	1	0	3.83	0.00	0.00	2.64	0.00	1.00	0.00	1.00	0.00
4pe1	1.12	1.04	0.08	1	0	0.02	0.00	1.00	0.00	0.00	1.00	0.44	0.00	0.00
4pe4	2.02	0.84	1.18	0	0	0.00	0.00	1.00	0.00	0.00	1.00	2.30	0.00	0.00
4pe7	0.95	0.95	0.00	1	0	2.44	0.00	1.00	4.33	0.00	1.00	0.00	0.00	1.00
4pi3	1.56	1.56	0.00	1	0	4.66	0.00	1.00	1.20	0.00	1.00	0.00	0.00	1.00
4pnc	1.99	1.21	0.78	1	0	0.04	0.00	1.00	1.00	1.00	0.00	0.00	1.00	0.00
4pok	2.03	1.08	0.95	0	0	0.24	0.00	1.00	0.00	0.00	1.00	2.12	0.00	0.00
4q8i	1.74	1.74	0.00	1	0	0.03	1.00	0.00	0.18	1.00	0.00	0.17	1.00	0.00
4qps	1.65	1.31	0.34	1	0	0.10	1.00	0.00	0.00	1.00	0.00	0.00	1.00	0.00
4qq5	1.64	1.64	0.00	1	0	0.28	1.00	0.00	0.06	0.00	1.00	0.00	1.00	0.00
4qqc	2.39	1.41	0.98	0	0	0.45	0.00	0.00	0.00	0.00	1.00	0.00	1.00	0.00
4rqx	1.85	0.94	0.91	1	0	0.00	1.00	0.00	0.00	0.00	1.00	0.00	0.00	1.00
4us1	1.62	1.62	0.00	1	0	0.00	0.00	1.00	4.32	0.00	0.00	3.95	0.00	0.00
4us2	1.95	1.60	0.35	1	0	0.21	1.00	0.00	0.00	1.00	0.00	1.06	0.00	0.00
4uuq	0.96	0.96	0.00	1	0	7.91	0.00	0.00	0.00	1.00	0.00	0.00	1.00	0.00
4wx6	3.54	2.25	1.29	0	1	0.00	1.00	0.00	0.98	1.00	0.00	1.32	0.00	0.00
4x0t	1.36	1.36	0.00	1	0	0.00	1.00	0.00	0.00	0.00	1.00	0.00	1.00	0.00
4x6t	2.03	1.37	0.66	0	0	0.14	0.00	1.00	0.00	1.00	0.00	0.00	0.00	1.00
4xcu	2.18	1.47	0.71	0	0	0.00	0.00	1.00	0.00	1.00	0.00	0.00	1.00	0.00
4xz0	3.49	0.97	2.52	0	0	1.41	0.00	1.00	0.00	0.00	1.00	0.00	0.00	1.00
4xz1	2.18	1.34	0.84	0	0	1.46	0.00	1.00	0.00	0.00	1.00	1.53	0.00	1.00
4yhf	2.10	1.66	0.44	0	0	0.01	1.00	0.00	0.00	0.00	1.00	0.00	1.00	0.00
4ym9	1.61	1.24	0.37	1	0	0.47	1.00	0.00	3.51	0.00	0.00	0.00	1.00	0.00
4yqm	1.67	1.67	0.00	1	0	0.00	0.00	1.00	0.00	0.00	1.00	7.36	0.00	0.00
4yqu	1.43	1.42	0.01	1	0	2.85	0.00	1.00	0.00	1.00	0.00	0.00	1.00	0.00
4yqv	1.48	1.11	0.36	1	0	0.57	1.00	0.00	0.00	0.00	1.00	0.00	1.00	0.00
4yrs	3.48	1.44	2.04	0	0	1.59	0.00	0.00	0.00	1.00	0.00	0.00	1.00	0.00
4yv8	3.21	1.93	1.27	0	0	4.28	0.00	0.00	7.63	0.00	0.00	2.42	0.00	1.00
4z16	2.00	1.80	0.20	0	0	0.00	1.00	0.00	0.28	1.00	0.00	1.36	0.00	1.00
4zzm	1.97	1.16	0.82	1	0	0.79	1.00	0.00	0.00	1.00	0.00	0.00	1.00	0.00
4zzo	1.97	1.58	0.39	1	0	0.36	1.00	0.00	0.00	1.00	0.00	0.33	1.00	0.00
5acb	1.86	1.78	0.08	1	0	3.93	0.00	1.00	0.59	1.00	0.00	1.45	0.00	1.00
5azv	2.57	1.42	1.15	0	0	4.16	0.00	0.00	0.00	0.00	1.00	0.00	1.00	0.00
5c5o	1.42	1.42	0.00	1	0	1.79	0.00	0.00	0.10	0.00	1.00	4.23	0.00	1.00
5c91	1.47	1.25	0.22	1	0	0.00	0.00	1.00	0.44	1.00	0.00	0.00	1.00	0.00
5ced	0.75	0.75	0.00	1	0	0.45	0.00	1.00	0.00	0.00	1.00	0.87	0.00	1.00
5d11	2.27	1.27	1.00	0	0	7.48	0.00	0.00	1.70	0.00	0.00	0.80	0.00	0.00
5dad	1.36	1.10	0.25	1	0	0.04	1.00	0.00	0.00	1.00	0.00	2.06	0.00	1.00
5daf	2.04	1.52	0.51	0	0	0.00	0.00	1.00	0.26	1.00	0.00	0.00	0.00	1.00
5dv6	0.68	0.68	0.00	1	0	0.00	0.00	1.00	0.00	1.00	0.00	6.73	0.00	0.00
5dv8	3.67	1.86	1.81	0	0	7.34	0.00	0.00	0.10	1.00	0.00	0.00	1.00	0.00
5e7r	4.28	1.67	2.61	0	0	0.14	1.00	0.00	0.21	0.00	1.00	0.00	1.00	0.00
5e93	1.55	1.28	0.27	1	0	0.05	1.00	0.00	0.59	1.00	0.00	0.00	1.00	0.00
5ea9	2.23	1.85	0.38	0	0	0.13	1.00	0.00	0.00	0.00	1.00	0.00	1.00	0.00
5eph	2.00	1.50	0.50	1	0	0.11	1.00	0.00	2.70	0.00	1.00	0.43	0.00	0.00
5f02	3.99	2.57	1.42	0	1	0.06	1.00	0.00	0.00	1.00	0.00	5.95	0.00	1.00
5f2e	2.50	1.24	1.27	0	0	0.45	1.00	0.00	1.03	0.00	0.00	0.00	1.00	0.00
5fed	1.30	1.30	0.00	1	0	0.78	0.00	1.00	0.00	1.00	0.00	0.30	0.00	1.00
5fx5	1.37	1.37	0.00	1	0	0.06	1.00	0.00	0.00	1.00	0.00	0.00	1.00	0.00
5fx6	1.68	1.68	0.00	1	0	0.00	0.00	1.00	0.00	1.00	0.00	0.00	1.00	0.00
5git	3.06	2.54	0.52	0	1	0.07	0.00	1.00	0.57	0.00	0.00	0.88	0.00	1.00
5gnk	1.80	1.68	0.12	1	0	0.07	1.00	0.00	0.02	1.00	0.00	0.00	1.00	0.00
5hg5	1.53	1.15	0.39	1	0	0.00	1.00	0.00	0.81	1.00	0.00	0.00	0.00	1.00
5hg7	1.66	1.34	0.32	1	0	0.04	1.00	0.00	0.00	1.00	0.00	0.00	1.00	0.00
5hg8	1.94	0.99	0.96	1	0	0.00	1.00	0.00	4.84	0.00	0.00	0.00	1.00	0.00
5hg9	2.27	1.31	0.96	0	0	0.00	1.00	0.00	0.00	1.00	0.00	0.00	1.00	0.00
5hze	1.08	1.08	0.00	1	0	3.38	0.00	1.00	0.00	1.00	0.00	0.54	1.00	0.00
5iyt	2.70	2.70	0.00	0	1	0.00	0.00	1.00	1.70	0.00	1.00	1.80	0.00	1.00
5j7s	4.71	1.17	3.54	0	0	0.59	1.00	0.00	1.21	1.00	0.00	0.00	1.00	0.00
5j87	1.64	1.64	0.00	1	0	0.00	1.00	0.00	0.00	1.00	0.00	1.96	0.00	1.00
5j8i	1.51	0.98	0.53	1	0	6.06	0.00	1.00	0.00	1.00	0.00	0.99	0.00	1.00
5j9y	1.12	1.08	0.04	1	0	1.78	0.00	0.00	0.00	1.00	0.00	0.00	1.00	0.00
5jk3	0.99	0.99	0.00	1	0	3.15	0.00	0.00	0.00	0.00	1.00	0.91	0.00	1.00
5kre	1.20	1.20	0.00	1	0	0.03	1.00	0.00	0.00	1.00	0.00	0.00	1.00	0.00
5l6o	1.92	0.73	1.19	1	0	0.00	1.00	0.00	0.00	0.00	1.00	0.00	1.00	0.00
5l6p	1.15	1.15	0.00	1	0	2.40	0.00	0.00	1.15	0.00	1.00	0.00	1.00	0.00
5lbg	3.02	3.02	0.00	0	1	0.00	1.00	0.00	0.00	1.00	0.00	0.00	1.00	0.00
5lcj	2.28	2.28	0.00	0	1	0.09	0.00	1.00	0.00	0.00	1.00	0.00	0.00	1.00
5lck	3.11	1.34	1.77	0	0	0.00	1.00	0.00	0.00	1.00	0.00	0.00	1.00	0.00
5mae	2.50	1.66	0.84	0	0	4.73	0.00	1.00	0.00	0.00	1.00	0.00	1.00	0.00
5maj	1.52	1.52	0.00	1	0	0.02	0.00	1.00	0.00	1.00	0.00	0.00	1.00	0.00
5mjb	1.36	0.55	0.80	1	0	0.04	0.00	1.00	0.00	0.00	1.00	1.17	1.00	0.00
5mqy	2.15	1.42	0.73	0	0	3.98	0.00	0.00	4.93	0.00	0.00	5.91	0.00	0.00
5n19	2.95	1.24	1.71	0	0	0.63	1.00	0.00	0.15	0.00	1.00	0.00	0.00	1.00
5o8u	2.17	1.73	0.45	0	0	0.13	1.00	0.00	0.22	1.00	0.00	0.00	1.00	0.00
5tdi	9.81	2.79	7.02	0	1	0.17	1.00	0.00	0.00	1.00	0.00	0.00	1.00	0.00
5th7	1.26	1.07	0.20	1	0	0.00	1.00	0.00	0.00	0.00	1.00	0.00	1.00	0.00
5toz	1.72	1.21	0.51	1	0	0.10	1.00	0.00	0.17	0.00	1.00	0.00	1.00	0.00
5ttu	1.92	0.77	1.15	1	0	0.00	1.00	0.00	0.25	1.00	0.00	0.00	1.00	0.00
5ttv	1.48	0.98	0.50	1	0	1.87	0.00	0.00	0.14	1.00	0.00	0.00	1.00	0.00
5ug8	2.64	1.31	1.33	0	0	0.33	1.00	0.00	0.62	1.00	0.00	0.00	1.00	0.00
5ug9	2.19	1.67	0.52	0	0	0.00	1.00	0.00	0.01	1.00	0.00	0.00	1.00	0.00
5ugc	3.68	0.96	2.72	0	0	0.00	0.00	1.00	0.00	1.00	0.00	0.00	1.00	0.00
5v6s	2.69	1.14	1.55	0	0	0.59	0.00	1.00	0.05	1.00	0.00	0.00	1.00	0.00
5v6v	1.01	0.72	0.29	1	0	0.00	1.00	0.00	0.64	0.00	1.00	0.00	0.00	1.00
5v71	2.21	1.53	0.68	0	0	1.92	0.00	1.00	0.00	1.00	0.00	0.78	0.00	0.00
5v9o	1.51	1.14	0.37	1	0	0.00	1.00	0.00	0.11	1.00	0.00	0.00	0.00	1.00

5vnd	1.57	1.42	0.15	1	0	1.96	0.00	1.00	0.22	1.00	0.00	0.00	1.00	0.00
5vqv	1.51	1.51	0.00	1	0	0.57	1.00	0.00	0.00	1.00	0.00	0.85	0.00	1.00
5vqx	1.71	1.71	0.00	1	0	0.30	1.00	0.00	0.00	1.00	0.00	0.00	1.00	0.00
5vqy	1.45	1.34	0.11	1	0	0.34	1.00	0.00	0.00	1.00	0.00	0.00	1.00	0.00
5w1y	1.77	0.83	0.94	1	0	0.98	0.00	1.00	0.00	1.00	0.00	0.00	0.00	1.00
5WFJ	1.05	1.05	0.00	1	0	3.96	0.00	0.00	0.00	1.00	0.00	3.05	0.00	0.00
6ay2	1.62	1.09	0.53	1	0	0.00	0.00	1.00	1.03	0.00	0.00	0.26	0.00	1.00
6ffn	3.35	2.37	0.99	0	1	0.09	1.00	0.00	2.06	0.00	0.00	3.77	0.00	0.00
8est	0.97	0.97	0.00	1	0	0.01	1.00	0.00	1.34	0.00	0.00	0.40	0.00	1.00

Table S14. The *P-deviations* distributions for all docking tools.

Frequency	MOE	GOLD	CovDock	ICM-Pro
0	191	205	242	215
1	102	52	46	36
2	23	29	12	31
3	10	10	8	14
4	3	14	7	10
5	0	10	7	6
6	0	4	3	7
7	1	3	2	5
8	0	1	2	1
9	0	0	1	1
10	0	1	0	3
11	0	0	0	0
12	0	1	0	0
13	0	0	0	1
14	0	0	0	0

Table S15. Parameters to profile a docking tool.

PBD	MOE1	MOE2	GOLD1	GOLD2	CovDock1	CovDock2	ICM-Pro1	ICM-Pro2	Cross-S	Cross-F	MOE-S-Only	MOE-F-Only	GOLD-S-Only	GOLD-F-Only	CovDock-S-Only	CovDock-F-Only	ICM-S-Only	ICM-F-Only	MOE-S-2	MOE-F-2	GOLD-S-2	GOLD-F-2	Cov-S-2	Cov-F-2	ICM-S-2	ICM-F-2
1atk	1	0	1	0	1	0	0	1	3	1	0	0	0	0	0	0	0	1	1	0	0	0	1	0	0	0
1au0	0	0	0	0	0	1	0	1	0	2	0	0	0	0	0	0	0	0	0	0	0	0	0	1	0	1
1au2	0	1	0	1	1	0	0	1	1	3	0	0	0	0	1	0	0	0	0	1	0	1	0	0	0	1
1au3	0	1	1	0	1	0	0	1	2	2	0	0	0	0	0	0	0	0	0	1	0	0	1	0	0	1
1awf	1	0	1	0	1	0	0	1	3	1	0	0	0	0	0	0	0	1	1	0	0	0	1	0	0	0
1ayu	0	1	0	0	0	0	0	1	0	2	0	0	0	0	0	0	0	0	0	1	0	0	0	0	0	1
1ayv	0	1	0	1	0	1	0	1	0	4	0	0	0	0	0	0	0	0	0	1	0	1	0	1	0	1
1ayw	1	0	0	1	0	1	0	1	1	3	1	0	0	0	0	0	0	0	0	0	0	1	0	1	0	1
1bgo	0	0	1	0	1	0	0	0	2	0	0	0	0	0	0	0	0	0	0	0	0	0	1	0	0	0
1cef	0	0	0	0	0	1	0	0	0	1	0	0	0	0	0	1	0	0	0	0	0	0	0	0	0	0
1ceg	1	0	1	0	1	0	1	0	4	0	0	0	0	0	0	0	0	0	1	0	0	0	1	0	1	0
1cqq	0	0	1	0	1	0	0	1	2	1	0	0	0	0	0	0	0	1	0	0	0	0	1	0	0	0
1csb	1	0	1	0	1	0	0	0	3	0	0	0	0	0	0	0	0	0	1	0	0	0	1	0	0	0
1f1j	0	0	0	1	1	0	1	0	2	1	0	0	0	1	0	0	0	0	0	0	1	0	1	0	1	0
1fh0	1	0	1	0	0	1	1	0	3	1	0	0	0	0	0	1	0	0	1	0	0	0	0	0	1	0
1gm	0	0	1	0	1	0	1	0	3	0	0	0	0	0	0	0	0	0	0	0	0	0	1	0	1	0
1h8i	0	1	0	1	0	0	0	1	0	3	0	0	0	0	0	0	0	0	0	1	0	1	0	0	0	1
1haz	0	1	0	1	0	0	1	0	1	2	0	0	0	0	0	0	1	0	0	1	0	1	0	0	0	0
1hvb	0	0	0	1	0	1	0	1	0	3	0	0	0	0	0	0	0	0	0	0	0	1	0	1	0	1
1hvy	1	0	0	0	1	0	1	0	3	0	0	0	0	0	0	0	0	0	1	0	0	0	1	0	1	0
1iau	1	0	0	1	0	1	1	0	2	2	0	0	0	0	0	0	0	0	1	0	1	1	0	1	1	0
1ju6	1	0	0	1	1	0	1	0	3	1	0	0	0	1	0	0	0	0	1	0	1	0	1	0	1	0
1khp	1	0	1	0	1	0	1	0	4	0	0	0	0	0	0	0	0	0	1	0	0	0	1	0	1	0
1khq	0	0	0	1	1	0	0	0	1	1	0	0	0	1	1	0	0	0	0	0	0	0	0	0	0	0
1ms6	0	1	1	0	1	0	0	0	2	1	0	1	0	0	0	0	0	0	0	0	0	0	1	0	0	0
1mt5	0	0	1	0	0	1	1	0	2	1	0	0	0	0	0	1	0	0	0	0	0	0	0	0	1	0
1nfz	1	0	1	0	1	0	0	1	3	1	0	0	0	0	0	0	0	1	1	0	0	0	1	0	0	0
1nl6	0	1	0	1	1	0	1	0	2	2	0	0	0	0	0	0	0	0	0	1	1	1	1	0	1	0
1nlj	0	1	1	0	0	0	0	0	1	1	0	1	1	0	0	0	0	0	0	0	0	0	0	0	0	0
1nms	1	0	1	0	1	0	1	0	4	0	0	0	0	0	0	0	0	0	1	0	0	0	1	0	1	0
1npz	0	1	0	1	1	0	1	0	2	2	0	0	0	0	0	0	0	0	0	1	1	1	1	0	1	0
1oce	1	0	0	1	0	1	0	1	1	3	1	0	0	0	0	0	0	0	0	0	0	1	0	1	0	1
1pw	0	0	0	0	1	0	0	0	1	0	0	0	0	0	1	0	0	0	0	0	0	0	0	0	0	0
1pwc	0	0	1	0	1	0	1	0	3	0	0	0	0	0	0	0	0	0	0	0	0	0	1	0	1	0
1pw	0	1	1	0	1	0	1	0	3	1	0	1	0	0	0	0	0	0	0	0	0	0	1	0	1	0
1pyo	1	0	0	1	1	0	1	0	3	1	0	0	0	1	0	0	0	0	1	0	1	0	1	0	1	0
1qhr	1	0	0	1	1	0	1	0	3	1	0	0	0	1	0	0	0	0	1	0	1	0	1	0	1	0
1qj1	1	0	1	0	1	0	1	0	4	0	0	0	0	0	0	0	0	0	1	0	0	0	1	0	1	0
1qj6	1	0	0	1	0	0	0	0	1	1	1	0	0	1	0	0	0	0	0	0	0	0	0	0	0	0
1qj7	0	0	0	0	0	0	0	0	0	0	0	0	0	0	0	0	0	0	0	0	0	0	0	0	0	0
1qtn	1	0	0	1	1	0	0	0	2	1	0	0	0	1	0	0	0	0	1	0	1	0	1	0	0	0
1rtl	0	1	0	1	0	0	0	1	0	3	0	0	0	0	0	0	0	0	0	1	0	1	0	0	0	1
1scw	1	0	0	1	0	1	1	0	2	2	0	0	0	0	0	0	0	0	1	0	1	1	0	1	1	0
1u9v	0	1	0	1	0	1	0	1	0	4	0	0	0	0	0	0	0	0	0	1	0	1	0	1	0	1
1u9	0	0	1	0	0	1	0	0	1	1	0	0	1	0	0	1	0	0	0	0	0	0	0	0	0	0
1u9x	0	1	0	1	1	0	0	0	1	2	0	0	0	0	1	0	0	0	0	1	0	1	0	0	0	0

1vm	1	0	0	1	0	1	0	1	1	3	1	0	0	0	0	0	0	0	0	0	0	1	0	1	0	1
1w8	1	0	0	0	0	1	1	0	2	1	0	0	0	0	0	1	0	0	1	0	0	0	0	0	1	0
1x83	0	0	0	1	0	1	1	0	1	2	0	0	0	0	0	0	1	0	0	0	0	1	0	1	0	0
1yqs	1	0	0	1	0	1	1	0	2	2	0	0	0	0	0	0	0	0	1	0	1	1	0	1	1	0
1z6f	0	1	0	1	0	1	0	1	0	4	0	0	0	0	0	0	0	0	0	1	0	1	0	1	0	1
1zlr	1	0	1	0	0	0	1	0	3	0	0	0	0	0	0	0	0	0	1	0	0	0	0	0	1	0
1zpz	0	1	0	1	0	1	0	1	0	4	0	0	0	0	0	0	0	0	0	1	0	1	0	1	0	1
1zsl	1	0	0	0	0	0	1	0	2	0	0	0	0	0	0	0	0	0	1	0	0	0	0	0	1	0
2a4q	0	1	1	0	0	0	0	1	1	2	0	0	1	0	0	0	0	0	0	1	0	0	0	0	0	1
2a5k	0	0	1	0	1	0	0	1	2	1	0	0	0	0	0	0	0	1	0	0	0	0	1	0	0	0
2awz	1	0	0	1	0	1	1	0	2	2	0	0	0	0	0	0	0	0	1	0	1	1	0	1	1	0
2ax0	0	0	0	1	1	0	0	0	1	1	0	0	0	1	1	0	0	0	0	0	0	0	0	0	0	0
2ax1	1	0	0	1	0	1	0	1	1	3	1	0	0	0	0	0	0	0	0	0	0	1	0	1	0	1
2c1e	0	0	0	1	0	0	0	1	0	2	0	0	0	0	0	0	0	0	0	0	0	1	0	0	0	1
2c2k	0	1	0	1	0	0	0	1	0	3	0	0	0	0	0	0	0	0	0	1	0	1	0	0	0	1
2c2o	0	1	0	0	0	0	0	1	0	2	0	0	0	0	0	0	0	0	0	1	0	0	0	0	0	1
2c2z	0	0	0	0	0	0	0	1	0	1	0	0	0	0	0	0	0	1	0	0	0	0	0	0	0	0
2cdr	0	0	0	1	0	1	0	1	0	3	0	0	0	0	0	0	0	0	0	0	0	1	0	1	0	1
2cnk	0	1	0	1	0	0	0	1	0	3	0	0	0	0	0	0	0	0	0	1	0	1	0	0	0	1
2cnl	0	0	0	1	0	0	0	1	0	2	0	0	0	0	0	0	0	0	0	0	0	1	0	0	0	1
2cnn	0	0	0	1	0	1	0	1	0	3	0	0	0	0	0	0	0	0	0	0	0	1	0	1	0	1
2cno	0	0	0	1	0	1	0	1	0	3	0	0	0	0	0	0	0	0	0	0	0	1	0	1	0	1
2ex6	1	0	0	1	0	0	0	0	1	1	1	0	0	1	0	0	0	0	0	0	0	0	0	0	0	0
2ex8	0	0	0	1	1	0	1	0	2	1	0	0	0	1	0	0	0	0	0	0	1	0	1	0	1	0
2ex9	1	0	1	0	1	0	1	0	4	0	0	0	0	0	0	0	0	0	1	0	0	0	1	0	1	0
2exb	1	0	0	1	0	1	0	1	1	3	1	0	0	0	0	0	0	0	0	0	0	1	0	1	0	1
2f9a	1	0	0	0	0	1	0	1	1	2	1	0	0	0	0	0	0	0	0	0	0	0	0	1	0	1
2fq9	0	0	1	0	1	0	0	0	2	0	0	0	0	0	0	0	0	0	0	0	0	0	1	0	0	0
2fra	1	0	1	0	1	0	0	1	3	1	0	0	0	0	0	0	0	1	1	0	0	0	1	0	0	0
2frq	0	1	0	1	1	0	0	1	1	3	0	0	0	0	1	0	0	0	0	1	0	1	0	0	0	1
2ft2	1	0	1	0	1	0	1	0	4	0	0	0	0	0	0	0	0	0	1	0	0	0	1	0	1	0
2fud	1	0	1	0	1	0	1	0	4	0	0	0	0	0	0	0	0	0	1	0	0	0	1	0	1	0
2fye	0	1	1	0	1	0	1	0	3	1	0	1	0	0	0	0	0	0	0	0	0	0	1	0	1	0
2g6d	0	0	1	0	0	0	0	1	1	1	0	0	1	0	0	0	0	1	0	0	0	0	0	0	0	0
2gke	1	0	1	0	1	0	0	1	3	1	0	0	0	0	0	0	0	1	1	0	0	0	1	0	0	0
2gkj	1	0	1	0	1	0	1	0	4	0	0	0	0	0	0	0	0	0	1	0	0	0	1	0	1	0
2h5s	1	0	0	1	0	1	0	1	1	3	1	0	0	0	0	0	0	0	0	0	0	1	0	1	0	1
2hw	0	0	0	1	0	1	0	1	0	3	0	0	0	0	0	0	0	0	0	0	0	1	0	1	0	1
2i03	0	0	1	0	1	0	0	1	2	1	0	0	0	0	0	0	0	1	0	0	0	0	1	0	0	0
2ijn	0	0	0	0	0	1	0	1	0	2	0	0	0	0	0	0	0	0	0	0	0	0	0	1	0	1
2ipp	0	1	0	1	0	1	0	1	0	4	0	0	0	0	0	0	0	0	0	1	0	1	0	1	0	1
2j8y	1	0	1	0	1	0	1	0	4	0	0	0	0	0	0	0	0	0	1	0	0	0	1	0	1	0
2jai	1	0	1	0	1	0	1	0	4	0	0	0	0	0	0	0	0	0	1	0	0	0	1	0	1	0
2jbf	1	0	1	0	1	0	0	0	3	0	0	0	0	0	0	0	0	0	1	0	0	0	1	0	0	0
2ml	0	1	0	1	1	0	1	0	2	2	0	0	0	0	0	0	0	0	0	1	1	1	1	0	1	0
2oc0	1	0	1	0	0	0	0	1	2	1	0	0	0	0	0	0	0	1	1	0	0	0	0	0	0	0
2oc1	0	0	1	0	1	0	0	1	2	1	0	0	0	0	0	0	0	1	0	0	0	0	1	0	0	0
2op3	0	0	1	0	1	0	0	0	2	0	0	0	0	0	0	0	0	0	0	0	0	0	1	0	0	0
2pre	1	0	0	1	1	0	0	1	2	2	0	0	0	0	0	0	0	0	1	0	1	1	1	0	0	1
2px6	0	0	0	0	0	0	0	1	0	1	0	0	0	0	0	0	0	1	0	0	0	0	0	0	0	0
2qlq	0	0	1	0	1	0	1	0	3	0	0	0	0	0	0	0	0	0	0	0	0	0	1	0	1	0
2qq7	1	0	1	0	1	0	0	1	3	1	0	0	0	0	0	0	0	1	1	0	0	0	1	0	0	0
2r4b	0	0	1	0	1	0	1	0	3	0	0	0	0	0	0	0	0	0	0	0	0	0	1	0	1	0
2r6n	1	0	1	0	1	0	0	0	3	0	0	0	0	0	0	0	0	0	1	0	0	0	1	0	0	0
2r9m	0	0	1	0	1	0	0	0	2	0	0	0	0	0	0	0	0	0	0	0	0	0	1	0	0	0
2r9n	0	0	1	0	1	0	1	0	3	0	0	0	0	0	0	0	0	0	0	0	0	0	1	0	1	0
2r9o	0	1	0	0	0	1	0	1	0	3	0	0	0	0	0	0	0	0	0	1	0	0	0	1	0	1
2tod	1	0	1	0	1	0	1	0	4	0	0	0	0	0	0	0	0	0	1	0	0	0	1	0	1	0
2v35	1	0	1	0	0	0	0	0	2	0	0	0	0	0	0	0	0	0	1	0	0	0	0	0	0	0
2vgj	1	0	1	0	1	0	1	0	4	0	0	0	0	0	0	0	0	0	1	0	0	0	1	0	1	0
2wap	1	0	1	0	1	0	1	0	4	0	0	0	0	0	0	0	0	0	1	0	0	0	1	0	1	0
2wke	1	0	0	1	0	1	0	1	1	3	1	0	0	0	0	0	0	0	0	0	0	1	0	1	0	1
2xk1	1	0	0	0	0	1	0	1	1	2	1	0	0	0	0	0	0	0	0	0	0	0	0	1	0	1
2xln	0	1	1	0	0	1	0	1	1	3	0	0	1	0	0	0	0	0	0	1	0	0	0	1	0	1
2xu1	0	0	1	0	0	1	0	1	1	2	0	0	1	0	0	0	0	0	0	0	0	0	0	1	0	1
2xu3	1	0	0	1	1	0	0	0	2	1	0	0	0	1	0	0	0	0	1	0	1	0	1	0	0	0
2xu4	0	0	1	0	1	0	1	0	3	0	0	0	0	0	0	0	0	0	0	0	0	0	1	0	1	0
2xu5	1	0	1	0	1	0	0	1	3	1	0	0	0	0	0	0	0	1	1	0	0	0	1	0	0	0
2y4a	0	0	1	0	0	1	1	0	2	1	0	0	0	0	0	1	0	0	0	0	0	0	0	0	1	0
2y59	1	0	0	1	0	1	0	1	1	3	1	0	0	0	0	0	0	0	0	0	0	1	0	1	0	1
2yj2	0	1	1	0	0	1	0	1	1	3	0	0	1	0	0	0	0	0	0	1	0	0	0	1	0	1
2yj8	0	1	1	0	1	0	0	1	2	2	0	0	0	0	0	0	0	0	0	1	0	0	1	0	0	1
2yj9	0	1	1	0	1	0	0	0	2	1	0	1	0	0	0	0	0	0	0	0	0	0	1	0	0	0
2yjb	0	1	1	0	1	0	0	1	2	2	0	0	0	0	0	0	0	0	0	1	0	0	1	0	0	1
2yjc	0	0	1	0	1	0	0	1	2	1	0	0	0	0	0	0	0	1	0	0	0	0	1	0	0	0
2zk1	0	0	0	1	0	1	0	1	0	3	0	0	0	0	0	0	0	0	0	0	0	1	0	1	0	1
2zk2	0	1	0	1	0	1	0	1	0	4	0	0	0	0	0	0	0	0	0	1	0	1	0	1	0	1
2zk3	0	0	0	1	0	1	0	1	0	3	0	0	0	0	0	0	0	0	0	0	0	1	0	1	0	1
2zk4	0	0	0	1	0	1	0	1	0	3	0	0	0	0	0	0	0	0	0	0	0	1	0	1	0	1
2zk5	1	0	1	0	1	0	1	0	4	0	0	0	0	0	0	0	0	0	1	0	0	0	1	0	1	0
3a3e	0	0	0	1	0	1	0	0	0	2	0	0	0	0	0	0	0	0	0	0	0	1	0	1	0	0
3a3f	0	0	0	0	1	0	0	0	1	0	0	0	0	0	1	0	0	0	0	0	0	0	0	0	0	0
3a3i	1	0	0	0	1	0	0	0	2	0	0	0	0	0	0	0	0	0	1	0	0	0	1	0	0	0
3b0r	1	0	1	0	0	1	1	0	3	1	0	0	0	0	0	1	0	0	1	0	0	0	0	0	1	0
3bcn	0	0	0	1	0	1	0	1	0	3	0	0	0	0	0	0	0	0	0	0	0	1	0	1	0	1
3beb	1	0	1	0	1	0	0	0	3	0	0	0	0	0	0	0	0	0	1	0	0	0	1	0	0	0
3bfc	1	0	0	1	0	0	0	0	1	1	1	0	0	1	0	0	0	0	0	0	0	0	0	0	0	0
3bff	1	0	0	0	1	0	0	0	2	0	0	0	0	0	0	0	0	0	1	0	0	0	1	0	0	0
3bfg	0	0	0	0	1	0	0	1	1	1	0	0	0	0	1	0	0	1	0	0	0	0	0	0	0	0
3bg8	1	0	0	1	1	0	0	1	2	2	0	0	0	0	0	0	0	0	1	0	1	1	1	0	0	1
3bjm	1	0	0	0	1	0	1	0	3	0	0	0	0	0	0	0	0	0	1	0	0	0	1	0	1	0
3blt	1	0	1	0	1	0	1	0	4	0	0	0	0	0	0	0	0	0	1	0	0	0	1	0	1	0
3blu	1	0	1	0	1	0	1	0	4	0	0	0	0	0	0	0	0	0	1	0	0	0	1	0	1	0
3bm	1	0	1	0	1	0	1	0	4	0	0	0	0	0	0	0	0	0	1	0	0	0	1	0	1	0
3bpf	1	0	0	1	0	0	0	1	1	2	1	0	0	0	0	0	0	0	0	0	0	1	0	0	0	1
3bp	1	0	1	0	0	0	0	0	2	0	0	0	0	0	0	0	0	0	1	0	0	0	0	0	0	0
3bw	0	1	0	1	1	0	0	1	1	3	0	0	0	0	1	0	0	0	0	1	0	1	0	0	0	1
3cg5	1	0	0	0	1	0	0	0	2	0	0	0	0	0	0	0	0	0	1	0	0	0	1	0	0	0

3d4f	0	0	0	1	0	1	0	1	0	3	0	0	0	0	0	0	0	0	0	0	0	1	0	1	0	1
3e0n	1	0	0	1	0	1	0	0	1	2	1	0	0	0	0	0	0	0	0	0	0	1	0	1	0	0
3e0p	0	1	0	1	0	1	0	1	0	4	0	0	0	0	0	0	0	0	0	1	0	1	0	1	0	1
3ejx	1	0	1	0	1	0	0	1	3	1	0	0	0	0	0	0	0	1	1	0	0	0	1	0	0	0
3ek	1	0	1	0	1	0	1	0	4	0	0	0	0	0	0	0	0	0	1	0	0	0	1	0	1	0
3fv7	0	0	0	1	0	1	0	1	0	3	0	0	0	0	0	0	0	0	0	0	0	1	0	1	0	1
3fvf	1	0	0	1	1	0	1	0	3	1	0	0	0	1	0	0	0	0	1	0	1	0	1	0	1	0
3fzc	1	0	0	1	0	1	0	1	1	3	1	0	0	0	0	0	0	0	0	0	0	1	0	1	0	1
3hgn	0	0	0	1	1	0	1	0	2	1	0	0	0	1	0	0	0	0	0	0	1	0	1	0	1	0
3hha	1	0	1	0	1	0	1	0	4	0	0	0	0	0	0	0	0	0	1	0	0	0	1	0	1	0
3hw	0	1	1	0	1	0	0	1	2	2	0	0	0	0	0	0	0	0	0	1	0	0	1	0	0	1
3i4a	1	0	1	0	1	0	1	0	4	0	0	0	0	0	0	0	0	0	1	0	0	0	1	0	1	0
3ika	0	0	0	1	1	0	0	1	1	2	0	0	0	0	1	0	0	0	0	0	0	1	0	0	0	1
3k84	0	0	0	0	1	0	1	0	2	0	0	0	0	0	0	0	0	0	0	0	0	0	1	0	1	0
3kjf	0	1	1	0	0	0	1	0	2	1	0	1	0	0	0	0	0	0	0	0	0	0	0	0	1	0
3kjn	0	1	1	0	1	0	0	1	2	2	0	0	0	0	0	0	0	0	0	1	0	0	1	0	0	1
3kjq	0	1	1	0	1	0	0	0	2	1	0	1	0	0	0	0	0	0	0	0	0	0	1	0	0	0
3kw	1	0	1	0	1	0	0	0	3	0	0	0	0	0	0	0	0	0	1	0	0	0	1	0	0	0
3kw	0	1	1	0	1	0	0	1	2	2	0	0	0	0	0	0	0	0	0	1	0	0	1	0	0	1
3kwz	0	0	0	0	1	0	0	1	1	1	0	0	0	0	1	0	0	1	0	0	0	0	0	0	0	0
3LJ6	0	0	1	0	1	0	1	0	3	0	0	0	0	0	0	0	0	0	0	0	0	0	1	0	1	0
3lok	1	0	0	1	1	0	0	0	2	1	0	0	0	1	0	0	0	0	1	0	1	0	1	0	0	0
3mz	1	0	0	1	1	0	0	1	2	2	0	0	0	0	0	0	0	0	1	0	1	1	1	0	0	1
3mze	1	0	0	0	0	1	1	0	2	1	0	0	0	0	0	1	0	0	1	0	0	0	0	0	1	0
3n4c	0	1	1	0	1	0	0	0	2	1	0	1	0	0	0	0	0	0	0	0	0	0	1	0	0	0
3o0u	0	0	1	0	1	0	1	0	3	0	0	0	0	0	0	0	0	0	0	0	0	0	1	0	1	0
3o1g	1	0	1	0	0	1	1	0	3	1	0	0	0	0	0	1	0	0	1	0	0	0	0	0	1	0
3of8	1	0	0	1	1	0	0	0	2	1	0	0	0	1	0	0	0	0	1	0	1	0	1	0	0	0
3of9	0	1	0	1	0	1	0	1	0	4	0	0	0	0	0	0	0	0	0	1	0	1	0	1	0	1
3orx	1	0	0	1	1	0	0	1	2	2	0	0	0	0	0	0	0	0	1	0	1	1	1	0	0	1
3orz	1	0	1	0	1	0	1	0	4	0	0	0	0	0	0	0	0	0	1	0	0	0	1	0	1	0
3otu	1	0	0	0	0	1	0	0	1	1	1	0	0	0	0	1	0	0	0	0	0	0	0	0	0	0
3ovx	1	0	1	0	0	0	0	0	2	0	0	0	0	0	0	0	0	0	1	0	0	0	0	0	0	0
3p8e	1	0	1	0	1	0	1	0	4	0	0	0	0	0	0	0	0	0	1	0	0	0	1	0	1	0
3pae	1	0	1	0	0	1	1	0	3	1	0	0	0	0	0	1	0	0	1	0	0	0	0	0	1	0
3s3j	0	1	0	1	0	1	1	0	1	3	0	0	0	0	0	0	1	0	0	1	0	1	0	1	0	0
3s3p	0	1	0	0	1	0	0	0	1	1	0	1	0	0	1	0	0	0	0	0	0	0	0	0	0	0
3s3q	1	0	0	1	1	0	1	0	3	1	0	0	0	1	0	0	0	0	1	0	1	0	1	0	1	0
3s3s	0	1	0	1	1	0	0	0	1	2	0	0	0	0	1	0	0	0	0	1	0	1	0	0	0	0
3svv	1	0	1	0	1	0	1	0	4	0	0	0	0	0	0	0	0	0	1	0	0	0	1	0	1	0
3szb	1	0	1	0	1	0	1	0	4	0	0	0	0	0	0	0	0	0	1	0	0	0	1	0	1	0
3t9t	1	0	1	0	1	0	1	0	4	0	0	0	0	0	0	0	0	0	1	0	0	0	1	0	1	0
3tyq	0	0	0	1	0	1	1	0	1	2	0	0	0	0	0	0	1	0	0	0	0	1	0	1	0	0
3u1i	0	1	0	1	0	1	0	1	0	4	0	0	0	0	0	0	0	0	0	1	0	1	0	1	0	1
3upn	0	1	0	1	0	1	0	1	0	4	0	0	0	0	0	0	0	0	0	1	0	1	0	1	0	1
3upo	0	1	0	0	0	0	0	1	0	2	0	0	0	0	0	0	0	0	0	1	0	0	0	0	0	1
3upp	1	0	1	0	1	0	0	1	3	1	0	0	0	0	0	0	0	1	1	0	0	0	1	0	0	0
3v4o	0	1	0	1	1	0	1	0	2	2	0	0	0	0	0	0	0	0	0	1	1	1	1	0	1	0
3v6r	0	0	0	0	1	0	0	1	1	1	0	0	0	0	1	0	0	1	0	0	0	0	0	0	0	0
3w2	1	0	1	0	0	0	1	0	3	0	0	0	0	0	0	0	0	0	1	0	0	0	0	0	1	0
3w2t	1	0	0	1	1	0	0	1	2	2	0	0	0	0	0	0	0	0	1	0	1	1	1	0	0	1
3x1i	1	0	0	1	1	0	0	1	2	2	0	0	0	0	0	0	0	0	1	0	1	1	1	0	0	1
3zcz	1	0	1	0	1	0	1	0	4	0	0	0	0	0	0	0	0	0	1	0	0	0	1	0	1	0
3zfz	1	0	1	0	1	0	0	0	3	0	0	0	0	0	0	0	0	0	1	0	0	0	1	0	0	0
3zim	0	1	0	0	1	0	1	0	2	1	0	1	0	0	0	0	0	0	0	0	0	0	1	0	1	0
4ax	0	0	0	1	1	0	1	0	2	1	0	0	0	1	0	0	0	0	0	0	1	0	1	0	1	0
4b4x	1	0	0	0	1	0	1	0	3	0	0	0	0	0	0	0	0	0	1	0	0	0	1	0	1	0
4ben	1	0	0	1	0	1	0	1	1	3	1	0	0	0	0	0	0	0	0	0	0	1	0	1	0	1
4d9u	1	0	0	1	1	0	1	0	3	1	0	0	0	1	0	0	0	0	1	0	1	0	1	0	1	0
4dki	0	0	0	1	0	1	1	0	1	2	0	0	0	0	0	0	1	0	0	0	0	1	0	1	0	0
4dm	0	1	1	0	1	0	0	0	2	1	0	1	0	0	0	0	0	0	0	0	0	0	1	0	0	0
4dm	1	0	0	1	1	0	0	0	2	1	0	0	0	1	0	0	0	0	1	0	1	0	1	0	0	0
4g5j	0	0	0	0	1	0	1	0	2	0	0	0	0	0	0	0	0	0	0	0	0	0	1	0	1	0
4gs6	0	1	0	1	0	0	1	0	1	2	0	0	0	0	0	0	1	0	0	1	0	1	0	0	0	0
4hav	0	1	0	1	0	0	0	0	0	2	0	0	0	0	0	0	0	0	0	1	0	1	0	0	0	0
4hax	0	0	0	1	1	0	0	0	1	1	0	0	0	1	1	0	0	0	0	0	0	0	0	0	0	0
4hay	1	0	0	1	0	0	0	1	1	2	1	0	0	0	0	0	0	0	0	0	0	1	0	0	0	1
4haz	0	0	0	1	1	0	1	0	2	1	0	0	0	1	0	0	0	0	0	0	1	0	1	0	1	0
4hct	1	0	0	0	1	0	1	0	3	0	0	0	0	0	0	0	0	0	1	0	0	0	1	0	1	0
4hcu	1	0	1	0	1	0	1	0	4	0	0	0	0	0	0	0	0	0	1	0	0	0	1	0	1	0
4i24	1	0	1	0	1	0	1	0	4	0	0	0	0	0	0	0	0	0	1	0	0	0	1	0	1	0
4jj7	1	0	0	1	0	1	0	1	1	3	1	0	0	0	0	0	0	0	0	0	0	1	0	1	0	1
4jj8	0	0	0	0	0	1	0	1	0	2	0	0	0	0	0	0	0	0	0	0	0	0	0	1	0	1
4jje	0	0	0	0	0	1	0	1	0	2	0	0	0	0	0	0	0	0	0	0	0	0	0	1	0	1
4kty	0	1	0	1	0	1	0	1	0	4	0	0	0	0	0	0	0	0	0	1	0	1	0	1	0	1
4li5	0	0	0	1	1	0	1	0	2	1	0	0	0	1	0	0	0	0	0	0	1	0	1	0	1	0
4lqm	1	0	1	0	1	0	1	0	4	0	0	0	0	0	0	0	0	0	1	0	0	0	1	0	1	0
4lv6	0	0	1	0	1	0	0	0	2	0	0	0	0	0	0	0	0	0	0	0	0	0	1	0	0	0
4lyf	0	0	0	0	0	0	1	0	1	0	0	0	0	0	0	0	1	0	0	0	0	0	0	0	0	0
4lyj	0	0	0	0	1	0	0	1	1	1	0	0	0	0	1	0	0	1	0	0	0	0	0	0	0	0
4m1	1	0	1	0	1	0	1	0	4	0	0	0	0	0	0	0	0	0	1	0	0	0	1	0	1	0
4m1s	1	0	1	0	1	0	1	0	4	0	0	0	0	0	0	0	0	0	1	0	0	0	1	0	1	0
4m1t	0	1	1	0	1	0	1	0	3	1	0	1	0	0	0	0	0	0	0	0	0	0	1	0	1	0
4m1	0	1	0	1	0	1	0	1	0	4	0	0	0	0	0	0	0	0	0	1	0	1	0	1	0	1
4m2	1	0	0	0	1	0	0	1	2	1	0	0	0	0	0	0	0	1	1	0	0	0	1	0	0	0
4m2	0	0	0	1	0	1	0	1	0	3	0	0	0	0	0	0	0	0	0	0	0	1	0	1	0	1
4mz	1	0	0	1	0	1	1	0	2	2	0	0	0	0	0	0	0	0	1	0	1	1	0	1	1	0
4mz	0	0	1	0	1	0	1	0	3	0	0	0	0	0	0	0	0	0	0	0	0	0	1	0	1	0
4on	0	0	1	0	1	0	1	0	3	0	0	0	0	0	0	0	0	0	0	0	0	0	1	0	1	0
4pdz	0	0	0	1	0	1	0	1	0	3	0	0	0	0	0	0	0	0	0	0	0	1	0	1	0	1
4pe0	1	0	0	0	0	1	1	0	2	1	0	0	0	0	0	1	0	0	1	0	0	0	0	0	1	0
4pe1	1	0	0	1	0	1	0	0	1	2	1	0	0	0	0	0	0	0	0	0	0	1	0	1	0	0
4pe4	0	0	0	1	0	1	0	0	0	2	0	0	0	0	0	0	0	0	0	0	0	1	0	1	0	0
4pe7	1	0	0	1	0	1	0	1	1	3	1	0	0	0	0	0	0	0	0	0	0	1	0	1	0	1
4pi3	1	0	0	1	0	1	0	1	1	3	1	0	0	0	0	0	0	0	0	0	0	1	0	1	0	1
4pnc	1	0	0	1	1	0	1	0	3	1	0	0	0	1	0	0	0	0	1	0	1	0	1	0	1	0
4pok	0	0	0	1	0	1	0	0	0	2	0	0	0	0	0	0	0	0	0	0	0	1	0	1	0	0
4q8i	1	0	1	0	1	0	1	0	4	0	0	0	0	0	0	0	0	0	1	0	0	0	1	0	1	0

4qps	1	0	1	0	1	0	1	0	4	0	0	0	0	0	0	0	0	0	1	0	0	0	1	0	1	0
4qq5	1	0	1	0	0	1	1	0	3	1	0	0	0	0	0	1	0	0	1	0	0	0	0	0	1	0
4qqc	0	0	0	0	0	1	1	0	1	1	0	0	0	0	0	1	1	0	0	0	0	0	0	0	0	0
4rqx	1	0	1	0	0	1	0	1	2	2	0	0	0	0	0	0	0	0	1	0	0	0	0	1	0	1
4us1	1	0	0	1	0	0	0	0	1	1	1	0	0	1	0	0	0	0	0	0	0	0	0	0	0	0
4us2	1	0	1	0	1	0	0	0	3	0	0	0	0	0	0	0	0	0	1	0	0	0	1	0	0	0
4uuq	1	0	0	0	1	0	1	0	3	0	0	0	0	0	0	0	0	0	1	0	0	0	1	0	1	0
4wx	0	1	1	0	1	0	0	0	2	1	0	1	0	0	0	0	0	0	0	0	0	0	1	0	0	0
4x0t	1	0	1	0	0	1	1	0	3	1	0	0	0	0	0	1	0	0	1	0	0	0	0	0	1	0
4x6t	0	0	0	1	1	0	0	1	1	2	0	0	0	0	1	0	0	0	0	0	0	1	0	0	0	1
4xcu	0	0	0	1	1	0	1	0	2	1	0	0	0	1	0	0	0	0	0	0	1	0	1	0	1	0
4xz0	0	0	0	1	0	1	0	1	0	3	0	0	0	0	0	0	0	0	0	0	0	1	0	1	0	1
4xz1	0	0	0	1	0	1	0	1	0	3	0	0	0	0	0	0	0	0	0	0	0	1	0	1	0	1
4yhf	0	0	1	0	0	1	1	0	2	1	0	0	0	0	0	1	0	0	0	0	0	0	0	0	1	0
4ym	1	0	1	0	0	0	1	0	3	0	0	0	0	0	0	0	0	0	1	0	0	0	0	0	1	0
4yq	1	0	0	1	0	1	0	0	1	2	1	0	0	0	0	0	0	0	0	0	0	1	0	1	0	0
4yqu	1	0	0	1	1	0	1	0	3	1	0	0	0	1	0	0	0	0	1	0	1	0	1	0	1	0
4yqv	1	0	1	0	0	1	1	0	3	1	0	0	0	0	0	1	0	0	1	0	0	0	0	0	1	0
4yrs	0	0	0	0	1	0	1	0	2	0	0	0	0	0	0	0	0	0	0	0	0	0	1	0	1	0
4yv8	0	0	0	0	0	0	0	1	0	1	0	0	0	0	0	0	0	1	0	0	0	0	0	0	0	0
4z16	0	0	1	0	1	0	0	1	2	1	0	0	0	0	0	0	0	1	0	0	0	0	1	0	0	0
4zzm	1	0	1	0	1	0	1	0	4	0	0	0	0	0	0	0	0	0	1	0	0	0	1	0	1	0
4zzo	1	0	1	0	1	0	1	0	4	0	0	0	0	0	0	0	0	0	1	0	0	0	1	0	1	0
5acb	1	0	0	1	1	0	0	1	2	2	0	0	0	0	0	0	0	0	1	0	1	1	1	0	0	1
5azv	0	0	0	0	0	1	1	0	1	1	0	0	0	0	0	1	1	0	0	0	0	0	0	0	0	0
5c5o	1	0	0	0	0	1	0	1	1	2	1	0	0	0	0	0	0	0	0	0	0	0	0	1	0	1
5c91	1	0	0	1	1	0	1	0	3	1	0	0	0	1	0	0	0	0	1	0	1	0	1	0	1	0
5ced	1	0	0	1	0	1	0	1	1	3	1	0	0	0	0	0	0	0	0	0	0	1	0	1	0	1
5d11	0	0	0	0	0	0	0	0	0	0	0	0	0	0	0	0	0	0	0	0	0	0	0	0	0	0
5dad	1	0	1	0	1	0	0	1	3	1	0	0	0	0	0	0	0	1	1	0	0	0	1	0	0	0
5daf	0	0	0	1	1	0	0	1	1	2	0	0	0	0	1	0	0	0	0	0	0	1	0	0	0	1
5dv6	1	0	0	1	1	0	0	0	2	1	0	0	0	1	0	0	0	0	1	0	1	0	1	0	0	0
5dv8	0	0	0	0	1	0	1	0	2	0	0	0	0	0	0	0	0	0	0	0	0	0	1	0	1	0
5e7r	0	0	1	0	0	1	1	0	2	1	0	0	0	0	0	1	0	0	0	0	0	0	0	0	1	0
5e93	1	0	1	0	1	0	1	0	4	0	0	0	0	0	0	0	0	0	1	0	0	0	1	0	1	0
5ea9	0	0	1	0	0	1	1	0	2	1	0	0	0	0	0	1	0	0	0	0	0	0	0	0	1	0
5eph	1	0	1	0	0	1	0	0	2	1	0	0	0	0	0	1	0	0	1	0	0	0	0	0	0	0
5f02	0	1	1	0	1	0	0	1	2	2	0	0	0	0	0	0	0	0	0	1	0	0	1	0	0	1
5f2e	0	0	1	0	0	0	1	0	2	0	0	0	0	0	0	0	0	0	0	0	0	0	0	0	1	0
5fed	1	0	0	1	1	0	0	1	2	2	0	0	0	0	0	0	0	0	1	0	1	1	1	0	0	1
5fx5	1	0	1	0	1	0	1	0	4	0	0	0	0	0	0	0	0	0	1	0	0	0	1	0	1	0
5fx6	1	0	0	1	1	0	1	0	3	1	0	0	0	1	0	0	0	0	1	0	1	0	1	0	1	0
5git	0	1	0	1	0	0	0	1	0	3	0	0	0	0	0	0	0	0	0	1	0	1	0	0	0	1
5gnk	1	0	1	0	1	0	1	0	4	0	0	0	0	0	0	0	0	0	1	0	0	0	1	0	1	0
5hg5	1	0	1	0	1	0	0	1	3	1	0	0	0	0	0	0	0	1	1	0	0	0	1	0	0	0
5hg7	1	0	1	0	1	0	1	0	4	0	0	0	0	0	0	0	0	0	1	0	0	0	1	0	1	0
5hg8	1	0	1	0	0	0	1	0	3	0	0	0	0	0	0	0	0	0	1	0	0	0	0	0	1	0
5hg9	0	0	1	0	1	0	1	0	3	0	0	0	0	0	0	0	0	0	0	0	0	0	1	0	1	0
5hze	1	0	0	1	1	0	1	0	3	1	0	0	0	1	0	0	0	0	1	0	1	0	1	0	1	0
5iyt	0	1	0	1	0	1	0	1	0	4	0	0	0	0	0	0	0	0	0	1	0	1	0	1	0	1
5j7s	0	0	1	0	1	0	1	0	3	0	0	0	0	0	0	0	0	0	0	0	0	0	1	0	1	0
5j87	1	0	1	0	1	0	0	1	3	1	0	0	0	0	0	0	0	1	1	0	0	0	1	0	0	0
5j8i	1	0	0	1	1	0	0	1	2	2	0	0	0	0	0	0	0	0	1	0	1	1	1	0	0	1
5j9y	1	0	0	0	1	0	1	0	3	0	0	0	0	0	0	0	0	0	1	0	0	0	1	0	1	0
5jk3	1	0	0	0	0	1	0	1	1	2	1	0	0	0	0	0	0	0	0	0	0	0	0	1	0	1
5kre	1	0	1	0	1	0	1	0	4	0	0	0	0	0	0	0	0	0	1	0	0	0	1	0	1	0
5l6o	1	0	1	0	0	1	1	0	3	1	0	0	0	0	0	1	0	0	1	0	0	0	0	0	1	0
5l6p	1	0	0	0	0	1	1	0	2	1	0	0	0	0	0	1	0	0	1	0	0	0	0	0	1	0
5lbg	0	1	1	0	1	0	1	0	3	1	0	1	0	0	0	0	0	0	0	0	0	0	1	0	1	0
5lcj	0	1	0	1	0	1	0	1	0	4	0	0	0	0	0	0	0	0	0	1	0	1	0	1	0	1
5lck	0	0	1	0	1	0	1	0	3	0	0	0	0	0	0	0	0	0	0	0	0	0	1	0	1	0
5mae	0	0	0	1	0	1	1	0	1	2	0	0	0	0	0	0	1	0	0	0	0	1	0	1	0	0
5maj	1	0	0	1	1	0	1	0	3	1	0	0	0	1	0	0	0	0	1	0	1	0	1	0	1	0
5mjb	1	0	0	1	0	1	1	0	2	2	0	0	0	0	0	0	0	0	1	0	1	1	0	1	1	0
5mq	0	0	0	0	0	0	0	0	0	0	0	0	0	0	0	0	0	0	0	0	0	0	0	0	0	0
5n19	0	0	1	0	0	1	0	1	1	2	0	0	1	0	0	0	0	0	0	0	0	0	0	1	0	1
5o8u	0	0	1	0	1	0	1	0	3	0	0	0	0	0	0	0	0	0	0	0	0	0	1	0	1	0
5tdi	0	1	1	0	1	0	1	0	3	1	0	1	0	0	0	0	0	0	0	0	0	0	1	0	1	0
5th7	1	0	1	0	0	1	1	0	3	1	0	0	0	0	0	1	0	0	1	0	0	0	0	0	1	0
5toz	1	0	1	0	0	1	1	0	3	1	0	0	0	0	0	1	0	0	1	0	0	0	0	0	1	0
5ttu	1	0	1	0	1	0	1	0	4	0	0	0	0	0	0	0	0	0	1	0	0	0	1	0	1	0
5ttv	1	0	0	0	1	0	1	0	3	0	0	0	0	0	0	0	0	0	1	0	0	0	1	0	1	0
5ug8	0	0	1	0	1	0	1	0	3	0	0	0	0	0	0	0	0	0	0	0	0	0	1	0	1	0
5ug9	0	0	1	0	1	0	1	0	3	0	0	0	0	0	0	0	0	0	0	0	0	0	1	0	1	0
5ugc	0	0	0	1	1	0	1	0	2	1	0	0	0	1	0	0	0	0	0	0	1	0	1	0	1	0
5v6s	0	0	0	1	1	0	1	0	2	1	0	0	0	1	0	0	0	0	0	0	1	0	1	0	1	0
5v6v	1	0	1	0	0	1	0	1	2	2	0	0	0	0	0	0	0	0	1	0	0	0	0	1	0	1
5v71	0	0	0	1	1	0	0	0	1	1	0	0	0	1	1	0	0	0	0	0	0	0	0	0	0	0
5v9o	1	0	1	0	1	0	0	1	3	1	0	0	0	0	0	0	0	1	1	0	0	0	1	0	0	0
5vnd	1	0	0	1	1	0	1	0	3	1	0	0	0	1	0	0	0	0	1	0	1	0	1	0	1	0
5vqv	1	0	1	0	1	0	0	1	3	1	0	0	0	0	0	0	0	1	1	0	0	0	1	0	0	0
5vqx	1	0	1	0	1	0	1	0	4	0	0	0	0	0	0	0	0	0	1	0	0	0	1	0	1	0
5vqy	1	0	1	0	1	0	1	0	4	0	0	0	0	0	0	0	0	0	1	0	0	0	1	0	1	0
5w1	1	0	0	1	1	0	0	1	2	2	0	0	0	0	0	0	0	0	1	0	1	1	1	0	0	1
5WF	1	0	0	0	1	0	0	0	2	0	0	0	0	0	0	0	0	0	1	0	0	0	1	0	0	0
6ay2	1	0	0	1	0	0	0	1	1	2	1	0	0	0	0	0	0	0	0	0	0	1	0	0	0	1
6ffn	0	1	1	0	0	0	0	0	1	1	0	1	1	0	0	0	0	0	0	0	0	0	0	0	0	0
8est	1	0	1	0	0	0	0	1	2	1	0	0	0	0	0	0	0	1	1	0	0	0	0	0	0	0

Table S16 Kinase domain distribution in BCDE and their PDB id

Domain	Kinase	PDB ID
Remote-Cys	CDK12	5ACB
Beta4-4	PIK3Ca	3ZIM

Catalytic-3	FGFR1	5VND
Hinge	FRFG4	4XCU,4QQC,4QQ5
	PDPK1	3ORX,3ORZ,3OTU
	MAP3K7	4GS6,5J8I,5JK3,5E7R,5J7S
DFG-3	MAP2K1	5HZE
	MAPK1	4ZZO,4ZZM,5LCJ,5LCK
	EPHB1	5MJB
Extended front pocket	MAPK14	5O8U
	EGFR	3IKA, 3W2Q,4I24, 4LI5, 4LQM, 4G5J,5HG7,5HG5,5HG8,5HG9,5FED, 5UG9,5UG8,5GNK,5UGC,5J9Y
	JAK3	4Z16,4QPS,5TOZ,5TTU,5TTV,5WFJ
Front pocket	MAPK10	3V6R
	ITK	3T9T,4HCT,4HCU
	BTK	4YHF,5J87
	EPHB3	5L6O,5L6P
	ERBB4	2R4B
	RPS6KA3	4D9U
P-loop	ZAP70	4XZ0,4XZ1
	SRC	2HWP,2QLQ,2QQ7,3LOK,3SVV,5D11
